# Supplementary material for: Structure and regulation of the nuclear exosome targeting complex guides RNA substrates to the exosome
Source: Mol Cell. 2022 Jul 7;82(13):2505–2518.e7. doi: 10.1016/j.molcel.2022.04.011 (PMC9278407; doi:10.1016/j.molcel.2022.04.011)
Supplement: Document S1. Figures S1–S6 and Tables S1–S3 [file mmc1.pdf]

Molecular Cell, Volume *82*

Supplemental information

Structure and regulation of the nuclear exosome targeting complex guides RNA substrates to the exosome

Piotr Gerlach, William Garland, Mahesh Lingaraju, Anna Salerno-Kochan, Fabien Bonneau, Jérôme Basquin, Torben Heick Jensen, and Elena Conti

Figure S1. NEXT^{S} and NEXT^{L} cryo-EM sample preparation and data processing (related to Figure 1)

A) Size exclusion chromatography and SDS-PAGE of the NEXT^{S} sample used for cryo-EM structure determination

B) Representative cryo-EM micrograph collected on a FEI Titan Krios operated at 300 kV, equipped with a K3 Summit camera.

C) Representative 2D class averages of the selected NEXT^{S} particles. Box size (B) = 384 pix × 1.09 Å = 420 Å

D) NEXT^{S} Cryo-EM data processing scheme. The classification strategy on the bottom left resulted in a 3D reconstruction of the overall NEXT^{S} homodimer at 4.5 Å while the strategy on the bottom right resulted in a 4.0 Å focused reconstruction of the ZCCHC8 dimerization module interacting with two MTR4 KOW domains.

E) Size exclusion chromatography and SDS-PAGE of the NEXT^{L} sample used for cryo-EM structure determination

F) Representative cryo-EM micrograph collected on a FEI Titan Krios operated at 300 kV, equipped with a K3 Summit camera.

G) Representative 2D class averages of the selected NEXT^{L} complete homodimer particles (processing scheme not shown). Box size (B) = 300 pix × 0.8512 Å = 255 Å

H) Cryo-EM data processing scheme. All pre-processing and processing steps were done in Relion. The selection strategy depicted on the bottom left of the scheme led to the 6.8 Å reconstruction of a single NEXT protomer, revealing features like the ZCCHC8 zinc-finger domain and RBM7-binding module. The strategy depicted on the bottom right of the scheme led to the 3.4 Å reconstruction of a single MTR4.

I) Origin of structural elements used to build composite model of the NEXT homodimer. MTR4 DExH-box together with the ZCCHC8 CTD (Puno and Lima, 2018), were rigid-body fitted and refined within the 3.4 Å density of the focused MTR4 density. The MTR4 KOW and the closer pair of the stalk α-helices were rigid-body fitted and refined within the 4.0 Å density of the focused NEXT dimerization module. The ZCCHC8 dimerization module, both AIM and AIM+, and the C-terminal portion of the coiled-coil α1 helices were built de novo in the 4.0 Å focused density. The MTR4 N-terminus (residues 75-97), the entire coiled-coil α1 helices (residues 45-77), the ZCCHC8 *zinc*-finger followed by α3 helix (residues 220-260), and two

short ZCCHC8 fragments tethering the RBM7-binding module to the MTR4 RecA2 domain, were modelled with the AlphaFold and rigid body fitted in either the overall 4.5 Å NEXT^S density, or the 6.8 Å resolution density of the single NEXT^L protomer. The RBM7-binding module (Falk et al., 2016) was rigid-body fitted in the 6.8 Å resolution density of the single NEXT^L protomer. The gray, graduated triangle below depicts model confidence in area of the composite model – higher for the rigid-body fitted MTR4 fragments and de novo built ZCCHC8 dimerization module, and lower for the AlphaFold predictions and RBM7-binding module rigid-body fitted in low resolution map.

Figure S2. NEXT^S cryo-EM data quality and structural characteristics of the ZCCHC8 dimerization module (related to Figure 2)

A) Comparison of the MTR4 arch domain conformation. The inward arch conformation is revealed by the cryo-EM structure of the apo human MTR4 from the NEXT^S protomer (blue), the crystal structure of the apo human MTR4 (green) (PDB:6IEG) (Wang et al., 2019), and the crystal structure of the apo yeast Mtr4 in complex with RNA but without ATP (grey) (PDB:2XGJ) (Weir et al., 2010). The yeast MTR4 from the exosome-Mtr4-pre60S assembly (yellow) (PDB:6FSZ) (Schuller et al., 2018) displays the outward arch conformation.

B) and C) Local resolution analysis of the overall (B) and focused (C) NEXT^S complex reconstruction. Distribution of local resolution was estimated with RELION and colored accordingly.

D) and E) Spherical angular distribution of the NEXT^S complex particles used in the final 3D auto-refinement yielding the overall (D) and focused (E) reconstruction.

F) and G) The 3D Fourier Shell Correlation plots generated with the Salk Institute software (Tan et al., 2017). The red line represents the estimated global masked half-map FSC curve indicating an overall resolution for the overall NEXT^S reconstruction (F) and for the focused NEXT^S reconstruction (G), according to the gold standard FSC cut off of 0.143 (Rosenthal and Henderson, 2003).

H) and I) Model vs. map FSC plots for the real space refined model of the overall NEXT^S reconstruction (H) and for the focused NEXT^S reconstruction (I).

J) AlphaFold prediction of the ZCCHC8 N-terminal homodimerization region 1-260 shows overall structural similarity to the cryo-EM map interpretation performed in this study. The first ~40 residues in each protomer remain unstructured, as predicted by Falk et al., 2016, and are followed by the coiled-coil helices α1. In line with our findings, AlphaFold predicted a ZCCHC8 dimerization module composed of the central β-sheet with swapped β1 strands. Regions colored in red and orange correspond to the ZCCHC8 dimerization module built *de novo* in the 4.0 Å focused cryo-EM reconstruction of the $NEXT^{S}$. In the absence of the MTR4 KOW, ZCCHC8 residues 185-220 immediately following the ZCCHC8 AIM domain (highlighted with dotted circles), are predicted to extend the dimerization module through addition of two short anti-parallel β-strands. This is the major difference between the Alpha Fold model of ZCCHC8 in isolation and the experimentally derived cryo-EM model of MTR4-bound ZCCHC8. However, it is possible to envision that this region in ZCCHC8 may indeed fold against the ZCCHC8 dimerization module in the absence of MTR4 (as predicted by AlphaFold) and undergo a conformational change upon MTR4 binding (as visualized in the cryo-EM map for the complex).

K) Structural comparison of ZCCHC8 residues 177-220 (red) as shown by our experimental model (top panel) with the predicted AlphaFold model (bottom panel). In our model, the region assumes an extended conformation that wraps around the MTR4 KOW (indicated as a transparent blue surface). In the AlphaFold model, the two short anti-parallel β-strands (encircled residues 210-220) extend the ZCCHC8 dimerization module β-sheet in the absence of the MTR4 KOW.

L) AlphaFold prediction of the ZCCHC8 region 1-260, represented as ribbon and color-coded according to per-residue measure of local confidence called predicted local distance difference test (pLDDT). High confidence residues with pLDDT > 80, on a scale 0-100, are colored blue. Residues with pLDDT in the range 60-80 are colored cyan. Low confidence residues with pLDDT < 60 are colored orange.

M) Plot on the left shows per-residue measure of local confidence (pLDDT) for the AlphaFold prediction of the ZCCHC8 region 1-260, and corresponds with the residue colors in panel L. Plot on the right shows predicted aligned error (PAE). Dark green areas of the plot correspond with well folded fragments of the protein. Light green areas of the plot highlight low accuracy in predicted relative positions of folded domains.

N) Comparison of the AIMs domains from human ZCCHC8 (this study), NRDE2 (Wang et al., 2019), and nuclear VCP-like (NVL) (Lingaraju et al., 2019a) along with *S. cerevisiae* Nop53 (Falk et al., 2017b) and Air2 (Falk et al., 2014). Sequence alignment reveals the consensus sequence of the AIM to be X-F/W-X-L/I/V/T-D-X-X-G/P. The C-terminal glycine or proline residue allows the ZCCHC8 chain to bend away from the AIM-binding site.

O) Comparison of the KOW-interacting regions from ZCCHC8 (177-221) and NRDE2 (162-207), extending beyond the AIM. ZCCHC8 (red) and NRDE2 (green) share several conserved residues (depicted as sticks) that enable them to follow a similar binding pattern on the MTR4 KOW surface (grey). AIM (ZCCHC8 residues 177-184) and AIM+ (ZCCHC8 residues 185-220) are highlighted with black lines. ZCCHC8 and NRDE2 sequence alignment, at the bottom of the panel, shows conserved residues within their KOW-interacting regions.

Figure S3. Monomerized NEXT is sufficient to target some RNA substrates for degradation (related to Figure 3)

A) Analytical gel filtration comparison of the ZCCHC8(41-337)/RBM7(RRM) unit and the ZCCHC8(177-337)/RBM7(RRM) unit, resolved on the ÄKTAmicro equipped with Superdex 200 Increase 3.2/300 column. Retention volumes of molecular weight standards are highlighted for reference with thin dotted lines: ferritin (440 kDa), aldolase (158 kDa), conalbumin (75 kDa), ovalbumin (44 kDa), carbonic anhydrase (29 kDa), ribonuclease A (13.7 kDa).

B) RT-qPCR analysis of representative NEXT complex target transcripts (*proDNAJB4, proKLF6, proRBM39, proTTC32, 3'ext U11, 3'ext SNORD83a*). RNA was isolated from *ZCCHC8-3F-mAID* cell lines stably integrated with full length MYC-ZCCHC8$^{1-707}$, truncated MYC-ZCCHC8$^{177-707}$ or an empty vector (ev) as a negative control. Cells were either mock or treated with auxin (IAA) for 12 hours to deplete the endogenous ZCCHC8-3F-mAID protein. Results were normalized to *GAPDH* mRNA levels and plotted relative to *ZCCHC8-3F-mAID + ev - IAA control* samples. Columns represent the average values of technical triplicates per sample with error bars denoting the standard deviation. Individual data values from replicates are indicated as points.

Figure S4. ZCCHC8 linkers tether the ZCCHC8 ZnF and the RBM7 RRM domain at the RNA entry site within the MTR4 RecA2 domain (related to Figure 4)

A) Zoom-in onto the ZCCHC8 zinc-finger (ZnF) predicted with AlphaFold and rigid body fit in the 6.8 Å map of the single NEXT^{L} protomer. Conserved ZnF residues C229, C232, H237, and C242 are represented as sticks. The zinc atom was placed after aligning the structure of N-terminal zinc-finger domain from the HIV-1 nucleocapsid protein (PDB:1HVN) (South and Summers, 1993), found with the PDBeFOLD as the most similar to the ZCCHC8 zinc-finger. The AlphaFold-predicted α3 helix (residues 248-260) stabilizes the zinc-finger from the bottom. Linker connecting the AIM+ region with the zinc-finger is marked with a black arrowhead. Continuous density for the RNA spanning from the MTR4 channel, next to the zinc-finger, and beyond towards the RBM7 RRM is highlighted as a black dotted line. At the current resolution it was not possible to build in the nucleotides.

B) Zoom-in view of the tubular density connecting the ZCCHC8 α3 helix with the conserved motif located within the RecA2 cavity. The density is marked with a black arrowhead and the protein linker is depicted as red dashed line. The density is shown at lower map threshold (Chimera) compared to panels A and C, in order to emphasize better the linker connection.

C) Zoom-in view of the tubular density connecting the C-terminus of the ZCCHC8 Pro-rich region with the short β-strand aligned on the RecA2 domain β-sheet. The density is marked with a black arrowhead and the protein linker is depicted as red dashed line.

D) Zoom-in views comparison of ZCCHC8, NRDE2, and Air2 motifs bound within the MTR4 RecA2 domain pocket. A conserved acidic pocket on the RecA2 domain surface had previously been shown to bind positively-charged residues of human NRDE2 (Wang et al., 2019) and yeast Air2 (Falk et al., 2014). We speculate that a small additional density we observe within that pocket corresponds to a similar conserved motif Arg-Tyr-His (residues 273-275), preceding the Pro-rich domain of ZCCHC8.

E) Zoom-in views comparison of ZCCHC8, NRDE2, and Air2 motifs aligning as a β-strand on the existing MTR4 RecA2 domain β-sheet. A conserved edge of the

RecA2 β-sheet had previously been shown to interact with a β-strand of human NRDE2 (Wang et al., 2019) and yeast Trf4 (Falk et al., 2014). We speculate that a density we observe at that RecA2 β-sheet edge can be occupied by a predicted β-strand of ZCCHC8 (residues 334-337), following the Pro-rich domain of ZCCHC8.

F) Sequence alignment of ZCCHC8 showing the conserved motifs interacting with the MTR4 RecA2 domain.

Figure S5. ZCCHC8 CTD occludes the MTR4 RNA exit channel

(related to Figure 5)

A) Local resolution analysis of the focused MTR4 reconstruction from the $NEXT^L$ dataset. Distribution of local resolution was estimated with RELION and colored accordingly.

B) Spherical angular distribution of the $NEXT^L$ particles used in the final focused 3D auto-refinement of the MTR4

C) The 3D FSC plot generated with the Salk Institute software (Tan et al., 2017). The red line represents the estimated global masked half-map FSC curve indicating the resolution for MTR4 reconstruction, according to the gold standard FSC cut off of 0.143 (Rosenthal and Henderson, 2003).

D) Model vs. map FSC plot for the real space refined model of the focused MTR4 reconstruction.

E) Superposition of the MTR4-ZCCHC8 CTD X-ray structure (PDB:6C90)(Puno and Lima, 2018) with the MTR4-exosome structure (PDB:6D6R)(Weick et al., 2018)

F) The very C-terminal residues of the ZCCHC8 CTD (683-700) occupy the same surface cavity near the MTR4 ATPase site as the MPP6 N-terminal α-helix (5-21).

G) The N-terminal region of the ZCCHC8 CTD (residues 659-670) occupies the MTR4 surface used by the exosome cap protein RRP4 during MTR4-exosome interaction.

H) The interface between the ZCCHC8 CTD and the MTR4 RNA exit channel. Residues F673, E674, and E676 resemble RNA structure keeping the CTD locked in a position and blocking the RNA exit channel.

Figure S6. NEXT^{S}-EXO cryo-EM sample preparation and data processing (related to Figure 6)

A) Size exclusion chromatography and 15% SDS-PAGE of the NEXT^{S}-EXO sample used for cryo-EM structure determination. NEXT^{S} components are labeled in red.

B) Representative cryo-EM micrograph collected on a FEI Titan Krios operated at 300 kV, equipped with a K2 Summit camera.

C) Representative 2D classes from the initial selection of the NEXT^{S}-EXO particles. Box size (B) = 400 pix × 1.35 Å = 540 Å

D) Cryo-EM data processing scheme. 3D classification of particles showed one class (190,644 particles) corresponding to only the exosome core complex while another class (94,214 particles) showed additional density at the top of the exosome core. Particles from the latter class were re-extracted with a larger box size and re-centered on the NEXT^{S} ZCCHC8 dimerization module. Upon further rounds of 2D classification, we obtained a small class of particles corresponding to a NEXT^{S} homodimer bound to two exosomes (5,633 particles) and a larger class corresponding to a NEXT^{S} homodimer bound to a single EXO13 (11,273 particles). 3D classification of all particles used in the last 2D classification step led to a 9.5 Å 3D reconstruction that could be interpreted by fitting the known atomic models. Further 3D auto-refinement of that reconstruction, focused on MTR4-EXO9 reached 8.0 Å resolution.

E) NEXT-EXO model fitted in the 8.0 Å 3D auto-refinement. Region boxed with the black line is shown on the right of the panel, upon zooming-in and slicing the density in order to highlight the RNA substrate (red density and cartoon) – the RNA molecule from the superposed MTR4-exosome structure (PDB:6D6R) (Weick et al., 2018).

F) Gallery of the single representative extracted particles corresponding with a 2D class of the EXO-NEXT-EXO assembly (top row) and a 2D class of the NEXT-EXO assembly (bottom row). White “ruler” lines are highlighting positions of the NEXT and exosome complexes.

G) Mean pixel intensities quantified with ImageJ for the regions corresponding with the NEXT and exosome complexes within 2D classes from panel F. Similar amount of signal for both exosomes, as compared with the NEXT complex (top graph),

confirms that the EXO-NEXT-EXO 2D classes indeed represent an assembly with two exosomes, and do not originate from misclassification of the NEXT-EXO assembly. Mean pixel intensity of the background surrounding the particles was subtracted from each value before plotting.

H) Analytical gel filtration analysis of competitive binding between the ZCCHC8 CTD and the EXO9-MPP6-RRP6^{N}-RRP47 to the NEXT^{S} complex. 500 pmols equimolar amounts of the indicated recombinant protein samples were resolved on the Superdex 200 Increase 3.2/300 column and the peak fractions analyzed on a Coomassie stained 15% SDS PAGE. EXO9-MPP6-RRP6^{N}-RRP47 competes out the ZCCHC8 CTD for binding the NEXT^{S} complex (compare peaks 3.1 and 3.2 with peaks 5.1 and 5.2). Peaks 3.2 and 5.2 represent ZCCHC8 CTD not bound to the NEXT^{S}.

Figure S1

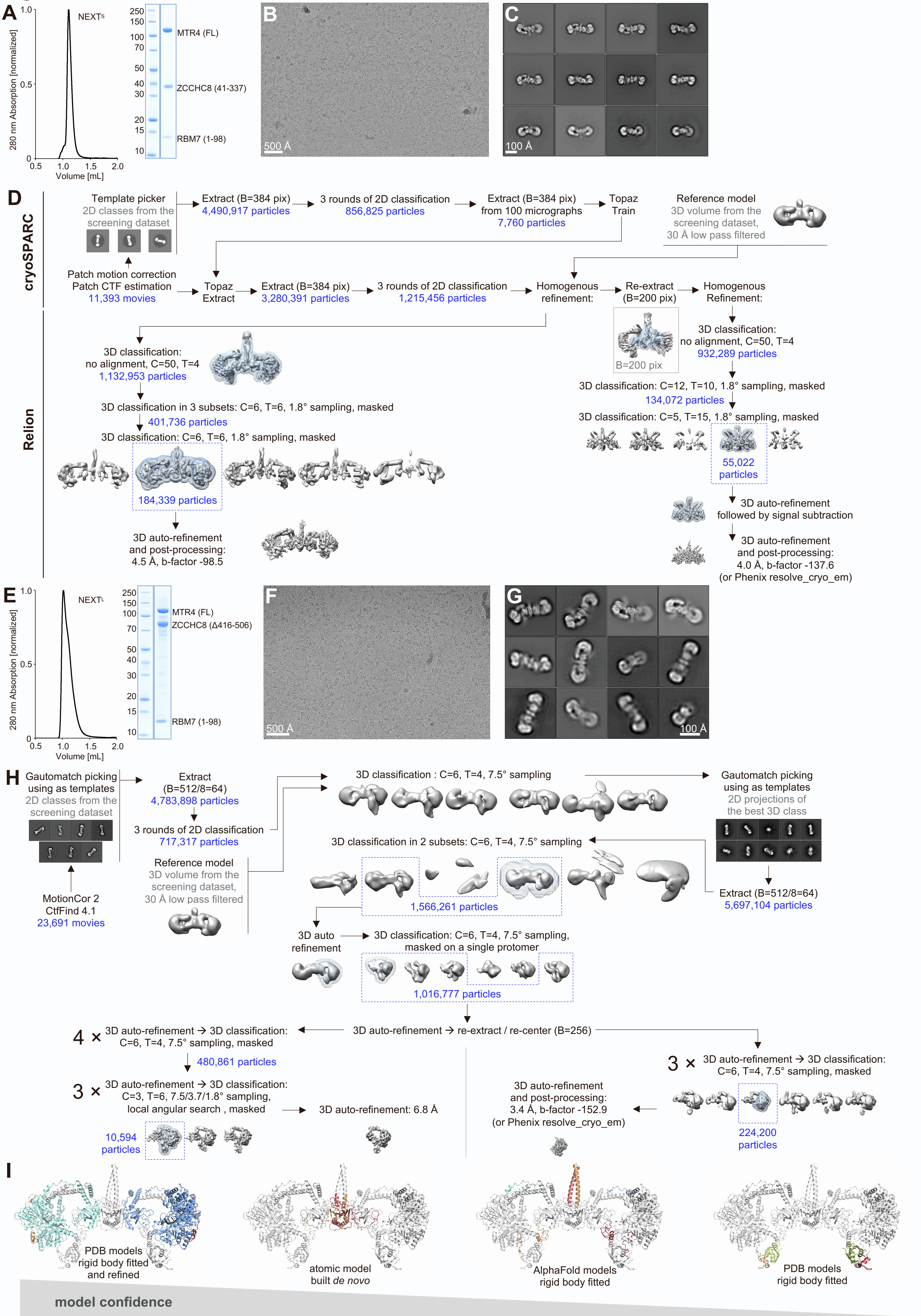
A
NEXTs
280 nm Absorption [normalized]
Volume [mL]
MTR4 (FL)
ZCCHC8 (41-337)
RBM7 (1-98)
B
500 Å
C
100 Å
D
cryoSPARC
Template picker
2D classes from the screening dataset
Extract (B=384 pix)
4,490,917 particles
3 rounds of 2D classification
856,825 particles
Extract (B=384 pix)
from 100 micrographs
7,760 particles
Topaz
Train
Reference model
3D volume from the screening dataset,
30 Å low pass filtered
Patch motion correction
Patch CTF estimation
11,393 movies
Topaz
Extract
Extract (B=384 pix)
3,280,391 particles
3 rounds of 2D classification
1,215,456 particles
Homogenous
refinement:
Re-extract
(B=200 pix)
Homogenous
Refinement:
B=200 pix
Relion
3D classification:
no alignment, C=50, T=4
1,132,953 particles
3D classification in 3 subsets: C=6, T=6, 1.8° sampling, masked
401,736 particles
3D classification: C=6, T=6, 1.8° sampling, masked
184,339 particles
3D auto-refinement
and post-processing:
4.5 Å, b-factor -98.5
3D classification:
no alignment, C=50, T=4
932,289 particles
3D classification: C=12, T=10, 1.8° sampling, masked
134,072 particles
3D classification: C=5, T=15, 1.8° sampling, masked
55,022
particles
3D auto-refinement
followed by signal subtraction
3D auto-refinement
and post-processing:
4.0 Å, b-factor -137.6
(or Phenix resolve_cryo_em)
E
NEXTL
MTR4 (FL)
ZCCHC8 (Δ416-506)
RBM7 (1-98)
280 nm Absorption [normalized]
Volume [mL]
F
500 Å
G
100 Å
H
Gautomatch picking
using as templates
2D classes from the screening dataset
MotionCor 2
CtfFind 4.1
23,691 movies
Extract
(B=512/8=64)
4,783,898 particles
3 rounds of 2D classification
717,317 particles
Reference model
3D volume from the screening dataset,
30 Å low pass filtered
3D classification : C=6, T=4, 7.5° sampling
Gautomatch picking
using as templates
2D projections of
the best 3D class
3D classification in 2 subsets: C=6, T=4, 7.5° sampling
1,566,261 particles
Extract (B=512/8=64)
5,697,104 particles
3D auto
refinement
3D classification: C=6, T=4, 7.5° sampling,
masked on a single protomer
1,016,777 particles
3D auto-refinement → re-extract / re-center (B=256)
4 × 3D auto-refinement → 3D classification:
C=6, T=4, 7.5° sampling, masked
480,861 particles
3 × 3D auto-refinement → 3D classification:
C=3, T=6, 7.5/3.7/1.8° sampling,
local angular search , masked
10,594
particles
3D auto-refinement: 6.8 Å
3 × 3D auto-refinement → 3D classification:
C=6, T=4, 7.5° sampling, masked
224,200
particles
3D auto-refinement
and post-processing:
3.4 Å, b-factor -152.9
(or Phenix resolve_cryo_em)
I
PDB models
rigid body fitted
and refined
atomic model
built de novo
AlphaFold models
rigid body fitted
PDB models
rigid body fitted
model confidence

Figure S2

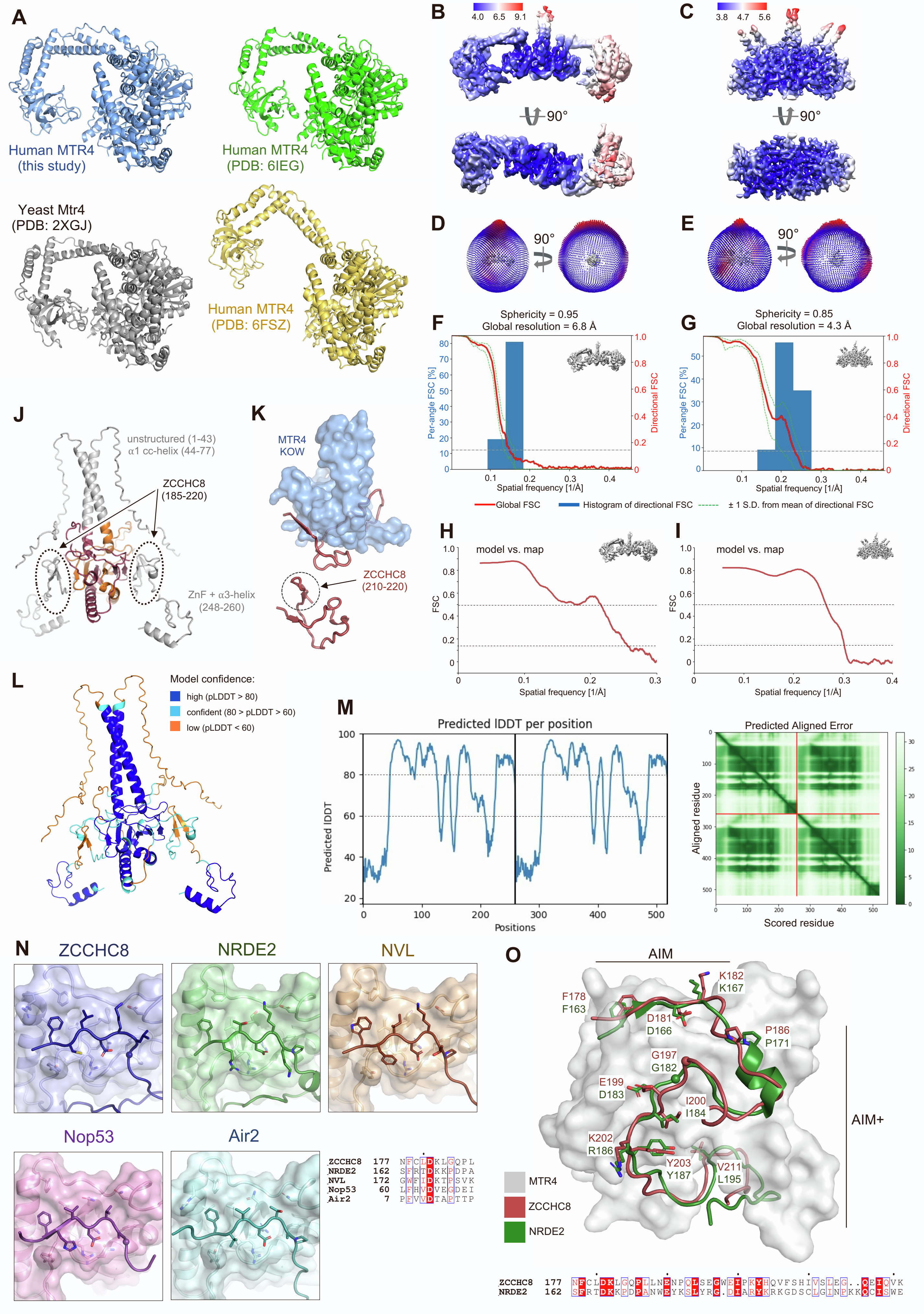

A
Human MTR4 (this study)
Human MTR4 (PDB: 6IEG)
Yeast Mtr4 (PDB: 2XGJ)
Human MTR4 (PDB: 6FSZ)
B
4.0 6.5 9.1
90°
C
3.8 4.7 5.6
90°
D
90°
E
90°
F
Sphericity = 0.95
Global resolution = 6.8 Å
Per-angle FSC [%]
Directional FSC
Spatial frequency [1/Å]
G
Sphericity = 0.85
Global resolution = 4.3 Å
Per-angle FSC [%]
Directional FSC
Spatial frequency [1/Å]
Global FSC
Histogram of directional FSC
± 1 S.D. from mean of directional FSC
H
model vs. map
FSC
Spatial frequency [1/Å]
I
model vs. map
FSC
Spatial frequency [1/Å]
J
unstructured (1-43)
α1 cc-helix (44-77)
ZCCHC8 (185-220)
ZnF + α3-helix (248-260)
K
MTR4 KOW
ZCCHC8 (210-220)
L
Model confidence:
high (pLDDT > 80)
confident (80 > pLDDT > 60)
low (pLDDT < 60)
M
Predicted lDDT per position
Predicted lDDT
Positions
Predicted Aligned Error
Aligned residue
Scored residue
N
ZCCHC8
NRDE2
NVL
Nop53
Air2
ZCCHC8 177 NFCLDKLGQPL
NRDE2 162 SFRTDKKPDPA
NVL 172 GWFIDKTPSVK
Nop53 60 LFHVDVEGDEI
Air2 7 PFVVDTAPTTP
O
AIM
AIM+
K182 K167
F178 F163
D181 D166
P186 P171
G197 G182
E199 D183
I200 I184
K202 R186
Y203 Y187
V211 L195
MTR4
ZCCHC8
NRDE2
ZCCHC8 177 NFCLDKLGQPLNENPQLSEGWEIPKYHQVFSHIVSLEG..QEIQVK
NRDE2 162 SFRTDKKPDPANWEYKSLYRG.DIARYKRKGDSCLGINPKKQCISWE

Figure S3

A

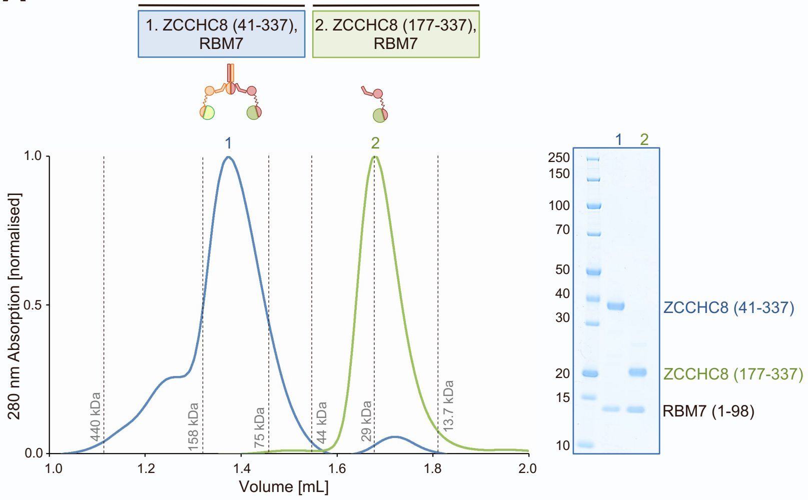

dimer
monomer
1. ZCCHC8 (41-337), RBM7
2. ZCCHC8 (177-337), RBM7
1
2
280 nm Absorption [normalised]
1.0
0.5
0.0
440 kDa
158 kDa
75 kDa
44 kDa
29 kDa
13.7 kDa
1.0
1.2
1.4
1.6
1.8
2.0
Volume [mL]
250
150
100
70
50
40
30
20
15
10
ZCCHC8 (41-337)
ZCCHC8 (177-337)
RBM7 (1-98)

B

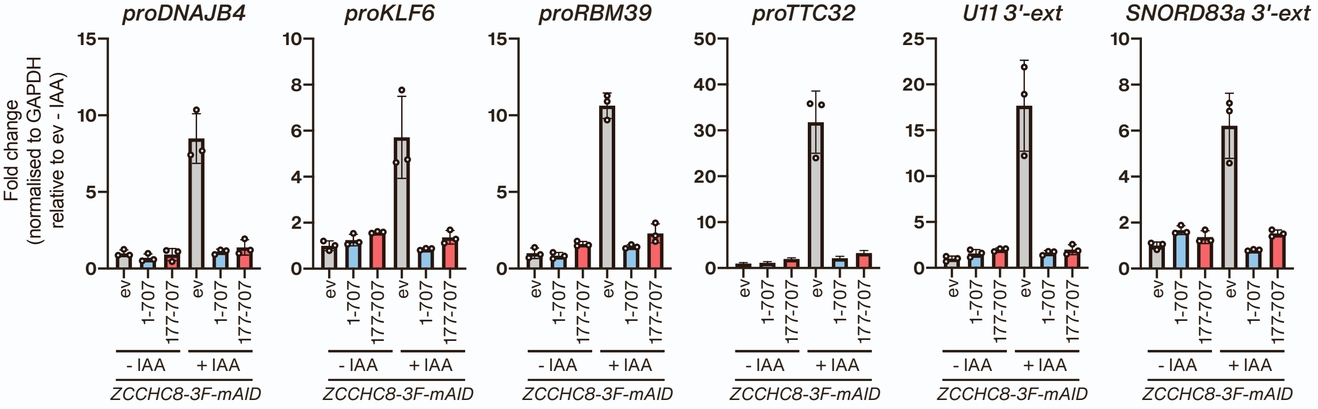

proDNAJB4
proKLF6
proRBM39
proTTC32
U11 3'-ext
SNORD83a 3'-ext
Fold change
(normalised to GAPDH
relative to ev - IAA)
ev
1-707
177-707
- IAA
+ IAA
ZCCHC8-3F-mAID

Figure S4

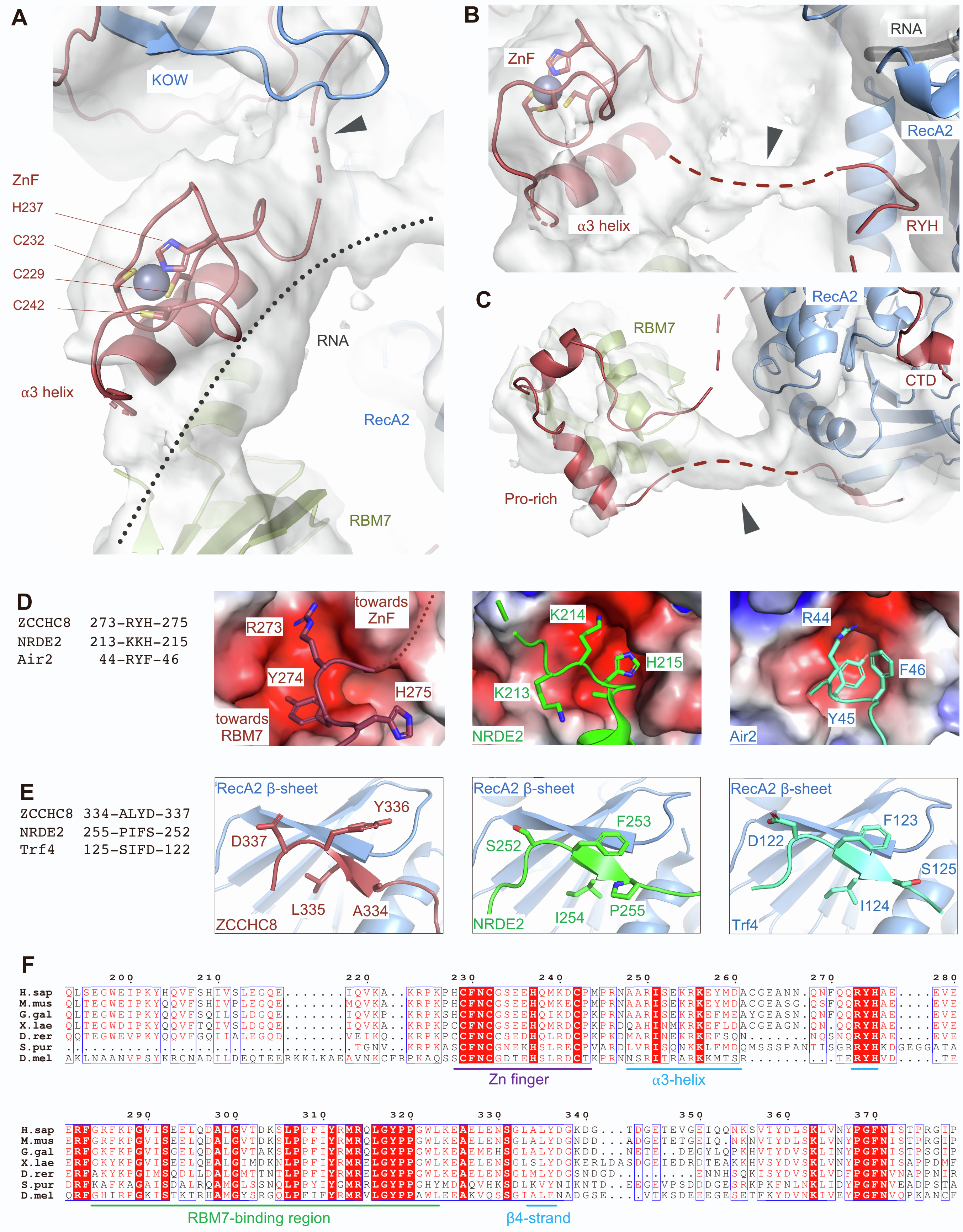
A
KOW
ZnF
H237
C232
C229
C242
α3 helix
RNA
RecA2
RBM7
B
ZnF
RNA
RecA2
α3 helix
RYH
C
RBM7
RecA2
CTD
Pro-rich
D
ZCCHC8 273-RYH-275
NRDE2 213-KKH-215
Air2 44-RYF-46
R273
towards ZnF
Y274
H275
towards RBM7
K214
H215
K213
NRDE2
R44
F46
Y45
Air2
E
ZCCHC8 334-ALYD-337
NRDE2 255-PIFS-252
Trf4 125-SIFD-122
RecA2 β-sheet
Y336
D337
L335
A334
ZCCHC8
F253
S252
I254
P255
NRDE2
F123
D122
S125
I124
Trf4
F
200 210 220 230 240 250 260 270 280
H.sap QLSEGWEIPKYHQVFSHIVSLEGQE.......IQVKA..KRPKPHCFNCGSEEHQMKDCPMPRNAARISEKRKEYMDACGEANN.QNFQQRYHAE....EVE
M.mus QLTEGWEIPKYQQVFSHIVPLEGQE.......MQVKA..KRPKPHCFNCGSEEHQMKECPMPRNAARISEKRKEYMDACGEASG.QSFQQRYHAE....EVE
G.gal QLTEGWEIPKYQQVFSQILSLDGQE.......IQVKP..KRPKSHCFNCGSEEHQIKDCPKPRNAARISEKRKEFMEAYGEASN.QNFQQRYHAE....EVE
X.lae QLTEGWDIPKYQQVFTQIVSLDGQE.......IQVKA..KRPKPCCFNCGSEEHQMRDCPKPRDQAHINMKRKEFLDACGEAGN.QN.QQRYHAE....EVE
D.rer QQTEGWEVPKYQQVFGQIIALEGQD.......VEIKQ..KRPKPCCFNCCSEDHQLRDCPKPKDMARINEKRKEFSQN.......NQSNQRYHAE....EVE
S.pur ....TGNV..KRPKASCFNCGNEKHSLRECPVARDLVRISQNKKLFMDQMSSSPANTISGRRYHKDGEGGATA
D.mel AKLNAANVPSYKRCNADILDEQTEERKKLKAEAVNKCFRPKAQSSCFNCGDTEHSLRDCTKPRNNSRITRARKKMTSR.........TERYHVD.....TE
Zn finger
α3-helix
290 300 310 320 330 340 350 360 370
H.sap ERFGRFKPGVISEELQDALGVTDKSLPPFIYRMRQLGYPPGWLKEAELENSGLALYDGKDG...TDGETEVGEIQQNKSVTYDLSKLVNYPGFNISTPRGIP
M.mus ERFGRFKPGVISEELQDALGVTDKSLPPFIYRMRQLGYPPGWLKEAELENSGLALYDGNDD...ADGETETGEIQ.NKNVTYDLSKLVNYPGFNISTPRGIP
G.gal ERFGKFKPGVISGELQDALGVTAKSLPPFIYRMRQLGYPPGWLKEAEMEHSGLALYDGKDD...NETE..DEGYLQPKHVTYDVSKLINYPGFNISTPSGIP
X.lae ERFGKYKPGVISEELQEALGIMDKNLPPFIYRMRELGYPPGWLKEAELENSGLSLYDGKERLDASDGEIEDRDTEAKKHVSYDVSKLVNYPGFNISAPPDMF
D.rer ERFAKYKPGIMSQDLLDALGMTTNTLPPFIYRMRELGYPPGWLKEAELENSGLMLYDGSNDGD.........ENNHSQKISYDVSKLVDFPGFNVNAPPNIR
S.pur DRFKAFKAGAISDALRQAMGLSSNQLPPYIYGMRRLGYPPGHYMDAQVHKSDLKVYNIKNTD..EEGEVPSDDDGESRKPKFNLNKLIDYPGFNVEADPSTA
D.mel QRFGHIRPGKISTKTRHAMGYSRGQLPFIFYRMRVLGYPPAWLEEAKVQSSGIALFNADGSE....VTKSDEEEGESETFKYDVNKIVEYPGFNVQPKANCF
RBM7-binding region
β4-strand

Figure S5

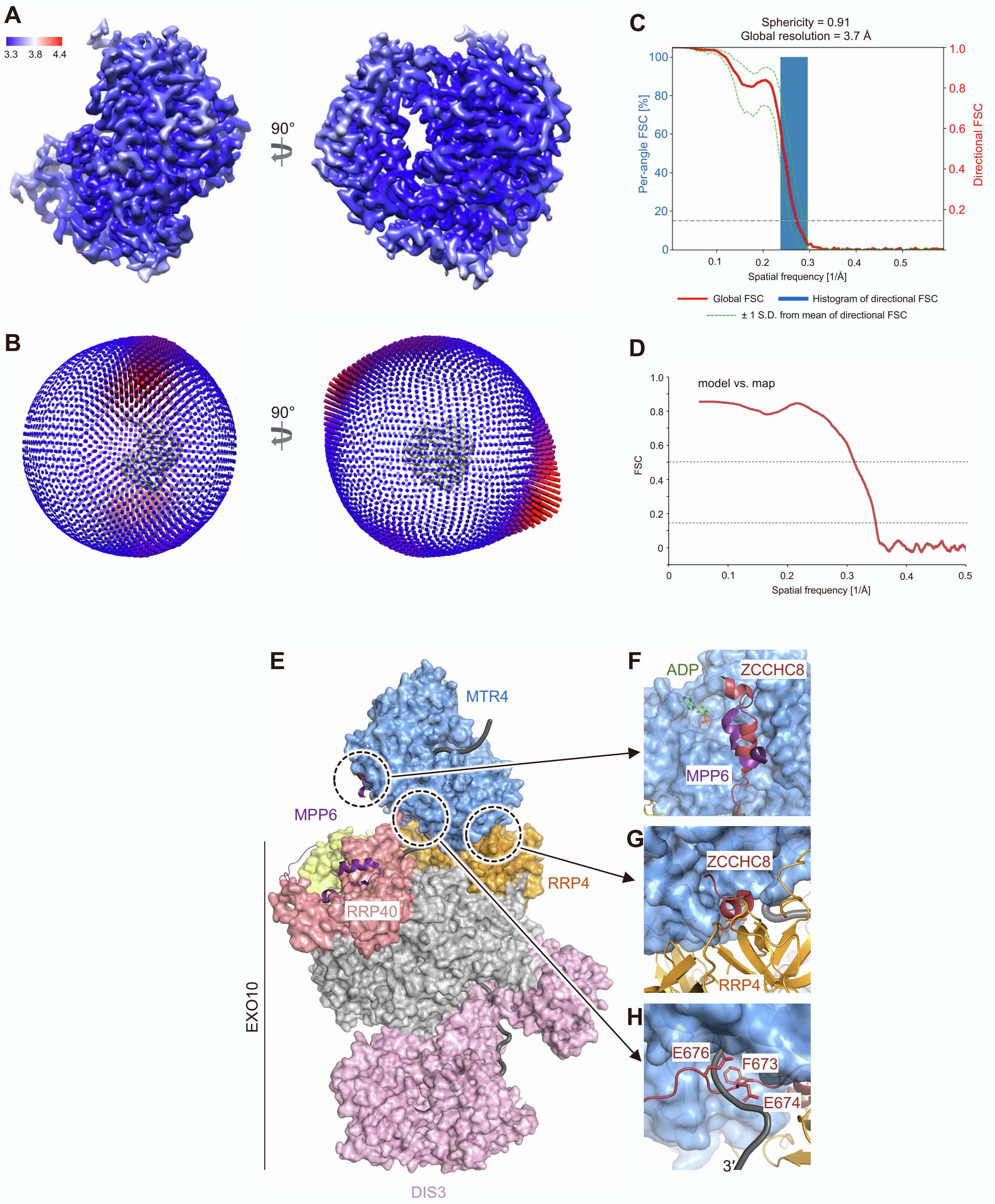
A
3.3 3.8 4.4
90°
B
90°
C
Sphericity = 0.91
Global resolution = 3.7 Å
Per-angle FSC [%]
Directional FSC
Spatial frequency [1/Å]
Global FSC
Histogram of directional FSC
± 1 S.D. from mean of directional FSC
D
model vs. map
FSC
Spatial frequency [1/Å]
E
MTR4
MPP6
RRP4
RRP40
EXO10
DIS3
F
ADP
ZCCHC8
MPP6
G
ZCCHC8
RRP4
H
E676
F673
E674
3'

Figure S6

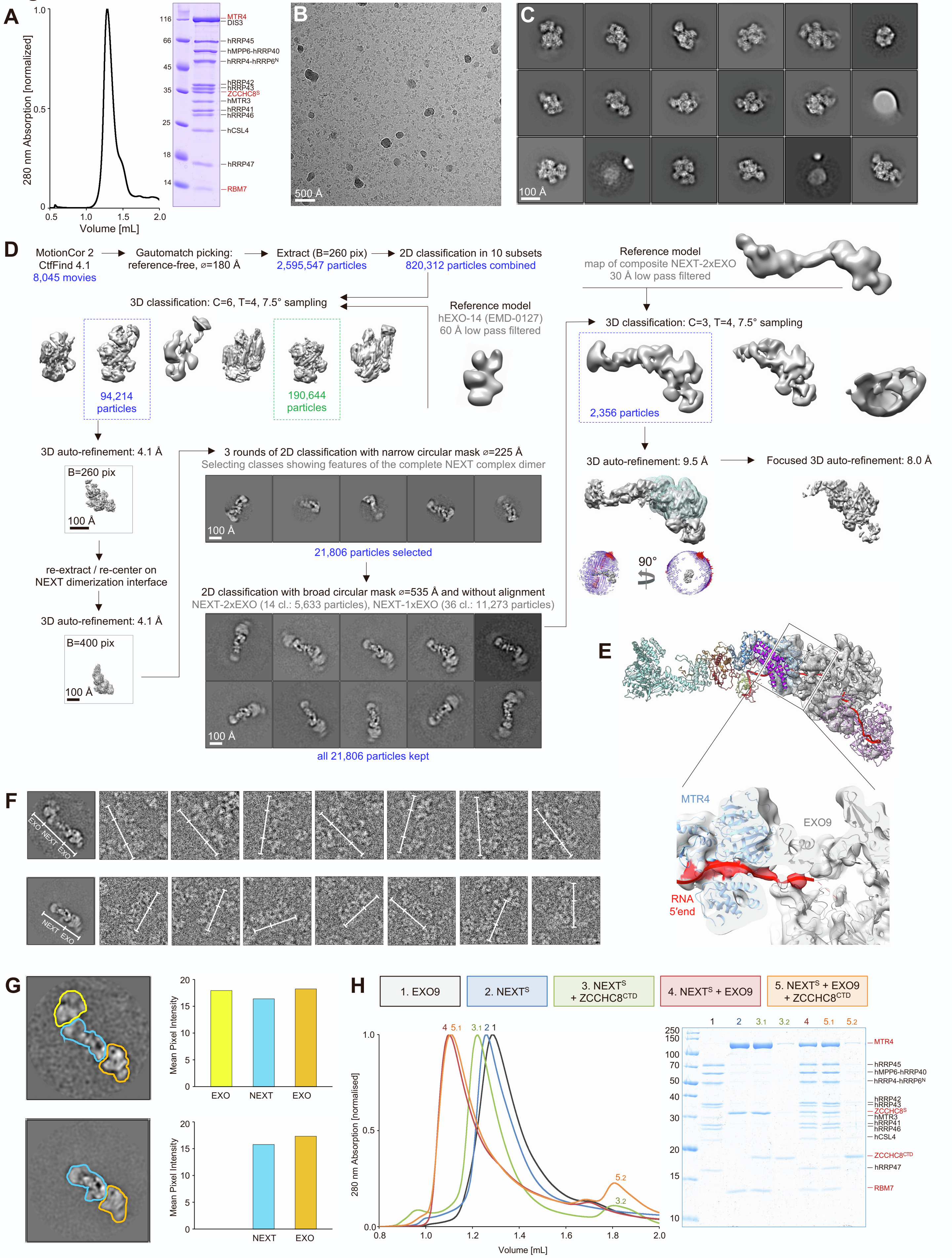
A
280 nm Absorption [normalized]
Volume [mL]
MTR4
DIS3
hRRP45
hMPP6-hRRP40
hRRP4-hRRP6N
hRRP42
hRRP43
ZCCHC8S
hMTR3
hRRP41
hRRP46
hCSL4
hRRP47
RBM7
B
500 Å
C
100 Å
D
MotionCor 2
CtfFind 4.1
8,045 movies
Gautomatch picking:
reference-free, ⌀=180 Å
Extract (B=260 pix)
2,595,547 particles
2D classification in 10 subsets
820,312 particles combined
3D classification: C=6, T=4, 7.5° sampling
Reference model
hEXO-14 (EMD-0127)
60 Å low pass filtered
94,214
particles
190,644
particles
3D auto-refinement: 4.1 Å
B=260 pix
re-extract / re-center on
NEXT dimerization interface
B=400 pix
3 rounds of 2D classification with narrow circular mask ⌀=225 Å
Selecting classes showing features of the complete NEXT complex dimer
21,806 particles selected
2D classification with broad circular mask ⌀=535 Å and without alignment
NEXT-2xEXO (14 cl.: 5,633 particles), NEXT-1xEXO (36 cl.: 11,273 particles)
all 21,806 particles kept
Reference model
map of composite NEXT-2xEXO
30 Å low pass filtered
3D classification: C=3, T=4, 7.5° sampling
2,356 particles
3D auto-refinement: 9.5 Å
Focused 3D auto-refinement: 8.0 Å
90°
E
MTR4
EXO9
RNA
5'end
F
EXO
NEXT
G
Mean Pixel Intensity
H
1. EXO9
2. NEXTS
3. NEXTS + ZCCHC8CTD
4. NEXTS + EXO9
5. NEXTS + EXO9 + ZCCHC8CTD
280 nm Absorption [normalised]
Volume [mL]
ZCCHC8CTD

Table S1. Cryo electron microscopy data collection summary, processing statistics and model quality indicators (related to Figures 1, 2 and 6)

Data collection	NEXT^{S}		NEXT^{L}		NEXT^{S} - EXO
Microscope	FEI Titan Krios GII				
Voltage (kV)	300				
Camera	Gatan K3				Gatan K2
Energy Filter	Gatan Quantum-LS (GIF)				
Pixel size (Å/pix)	1.094		0.851		1.350
Defocus range (µm)	0.5 – 2.3				
**3D reconstruction**	**NEXT^{S} overall reconstruction**	**NEXT^{S} dimerization module**	**NEXT^{L} single protomer**	**NEXT^{L} focused on MTR4**	**NEXT^{S} with nuclear exosome**
Number of movies	11,393		23,691		8,245
Number of frames/movie	31		40		40
Exposure time (sec)	4.65		6		10
Total exposure ($e^-/Å^2$)	64.85		68.20		46.86
Picked particle candidates	3,280,391		5,697,104		2,595,547
Final number of particles	184,339	55,022	10,594	224,200	2,356
Resolution (Å)[a]	4.5	4.0	6.8	3.4	9.5
Local resolution range (Å)	4.0 – 9.1	3.8 – 5.6	-	3.3 – 4.4	-
Sharpening B-factor	-98.5	-137.6	-210	-152.9	-
EMDB code	EMD-14510	EMD-14511	EMD-14514	EMD-14513	EMD-14515
**Refinement**					
PDB code	7Z4Y	7Z4Z		7Z52	
No atoms	16,762	4,828		5,911	
Residues (protein)	2,197	673		746	
Residues (RNA)	0	0		5	
CC_{box}, CC_{mask}, CC_{volume}[b]	0.69, 0.68, 0.68	0.57, 0.72, 0.69		0.65, 0.81, 0.77	
Resolution (Å)[b] FSC model vs. map (0 / 0.143 / 0.5)	3.4 / 3.9 / 5.6	3.2 / 3.3 / 3.8		2.5 / 2.9 / 3.2	
Bond lengths RMSD (Å)	0.003	0.002		0.004	
Bond angles RMSD (°)	0.761	0.700		0.601	
Ramachandran favored (%)	98.02	97.88		98.24	
Ramachandran allowed (%)	1.75	1.97		1.63	
Ramachandran outliers (%)	0.23	0.15		0.14	
MolProbity score	1.66	1.53		1.46	
Clash score	14.38	9.40		8.49	

[a]according to the Fourier Shell Correlation (FSC) cut-off criterion of 0.143 defined in (Rosenthal and Henderson, 2003)
[b]according to the model vs. map Correlation Coefficient definitions in (Afonine et al., 2018)

Table S2: sgRNA oligonucleotides (related to Figure 3)

NAME	FORWARD	REVERSE
ZCCHC8_gRNA	CACCGTAAGTCAAGCCATTATTCAG	AAACCTGAATAATGGCTTGACTTAC

Table S3: RTqPCR oligonucleotides (related to Figure 3)

NAME	FORWARD	REVERSE
proDNAJB4	TTTCTGGCGTTTCTGATTGA	ACCAAAACGCAGGTTGTTTA
proKLF6	AAGTTTTAGAGGGTCCGGCA	CTCTGCATAACCTTCACCG
proRBM39	AATAGATTTCCCTGTCATTTGGAGC	TTTCCAAGGTTGTTTCAAAGCTCG
proTTC32	GTCTGTTCCACGGTCCAAAC	ACAGCAGGCATGTAGGGTAG
U11 3'-ext	ACGCGTTTGGAGTAAGTGGT	GGTCACCTGCGGTTCATACA
SNORD83a 3'-ext	AGGGGAGACCTGTGGGTAAT	TGACCCCTTCCTGCTACTCA
GAPDH	GTCAGCCGCATCTTCTT	GCGCCCAATACGACCAAATC
