# Supplementary material for: Structure and regulation of the nuclear exosome targeting complex guides RNA substrates to the exosome
Source: Mol Cell. 2022 Jul 7;82(13):2505–2518.e7. doi: 10.1016/j.molcel.2022.04.011 (PMC9278407; doi:10.1016/j.molcel.2022.04.011)
Supplement: Document S2. Article plus supplemental information [file mmc2.pdf]

Molecular Cell

Article

Structure and regulation of the nuclear exosome targeting complex guides RNA substrates to the exosome

Graphical abstract

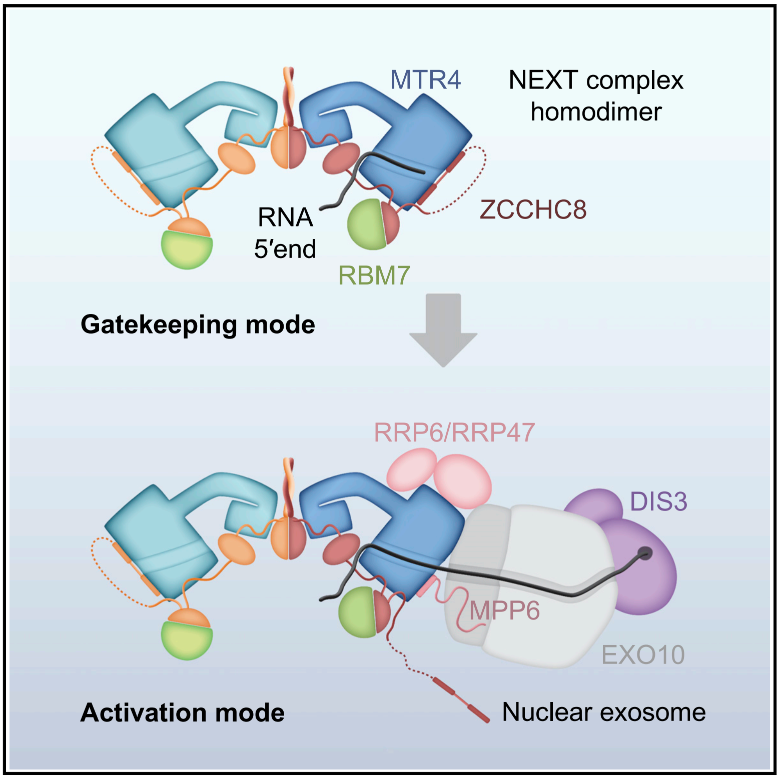

Highlights

- NEXT homodimerizes through two intertwined ZCCHC8 subunits
- ZCCHC8 binds MTR4 with both constitutive and regulatory interactions
- Stable MTR4 arch interactions orient the two helicases in opposite directions
- Regulatory interactions at the MTR4 helicase domain guide RNA to the exosome

Authors

Piotr Gerlach, William Garland, Mahesh Lingaraju, ..., Jérôme Basquin, Torben Heick Jensen, Elena Conti

Correspondence

p.gerlach@imol.edu.pl (P.G.), conti@biochem.mpg.de (E.C.)

In brief

Gerlach et al. show that the human NEXT complex homodimerizes *in vivo* and *in vitro* via two intertwined ZCCHC8 subunits. The ZCCHC8 C terminus restricts the movement of the RNA substrate and is displaced to allow the RNA 3′ end to access the human nuclear exosome for degradation.

Gerlach et al., 2022, Molecular Cell *82*, 2505–2518
July 7, 2022 © 2022 The Authors. Published by Elsevier Inc.
https://doi.org/10.1016/j.molcel.2022.04.011

CellPress

Molecular Cell

CellPress
OPEN ACCESS

Article

Structure and regulation of the nuclear exosome targeting complex guides RNA substrates to the exosome

Piotr Gerlach,[1,3,*] William Garland,[2] Mahesh Lingaraju,[1] Anna Salerno-Kochan,[1] Fabien Bonneau,[1] Jérôme Basquin,[1] Torben Heick Jensen,[2] and Elena Conti[1,4,*]

[1]Department of Structural Cell Biology, Max Planck Institute of Biochemistry, Am Klopferspitz 18, Martinsried, Munich, Germany
[2]Department of Molecular Biology and Genetics, Aarhus University, Aarhus, Denmark
[3]Present address: ReMedy International Research Agenda Unit, IMol Polish Academy of Sciences, Warsaw, Poland
[4]Lead contact
*Correspondence: p.gerlach@imol.edu.pl (P.G.), conti@biochem.mpg.de (E.C.)
https://doi.org/10.1016/j.molcel.2022.04.011

SUMMARY

In mammalian cells, spurious transcription results in a vast repertoire of unproductive non-coding RNAs, whose deleterious accumulation is prevented by rapid decay. The nuclear exosome targeting (NEXT) complex plays a central role in directing non-functional transcripts to exosome-mediated degradation, but the structural and molecular mechanisms remain enigmatic. Here, we elucidated the architecture of the human NEXT complex, showing that it exists as a dimer of MTR4-ZCCHC8-RBM7 heterotrimers. Dimerization preconfigures the major MTR4-binding region of ZCCHC8 and arranges the two MTR4 helicases opposite to each other, with each protomer able to function on many types of RNAs. In the inactive state of the complex, the 3′ end of an RNA substrate is enclosed in the MTR4 helicase channel by a ZCCHC8 C-terminal gatekeeping domain. The architecture of a NEXT-exosome assembly points to the molecular and regulatory mechanisms with which the NEXT complex guides RNA substrates to the exosome.

INTRODUCTION

The RNA exosome is a central player in RNA metabolism. This multiprotein complex is the main 3′→5′ ribonuclease in eukaryotic cells and is known to act on essentially all major types of nuclear and cytoplasmic RNAs (Mitchell et al., 1997; and reviewed in Chlebowski et al., 2013). In the vast majority of cases, the exosome progressively erodes the body of RNA substrates until degradation is complete, thereby eliminating defective transcripts and regulating transcript levels in RNA quality-control and RNA turnover pathways (reviewed in Ogami et al., 2018; Schmid and Jensen, 2018; Kilchert et al., 2016). The exosome can also participate in the RNA maturation process by partially trimming and processing the 3′ ends of a subset of RNA precursors (reviewed in Lingaraju et al., 2019b; Zinder and Lima, 2017). These cellular functions are conserved, although the number and variety of exosome substrates have increased throughout evolution. Correspondingly, the molecular architecture of the exosome is to a large extent conserved from lower to higher eukaryotes, but with an increased diversification and complexity in the case of the human complex.

Biochemical and structural studies on the yeast and human exosome core complexes have revealed similar mechanistic principles. The basal core of the 10 subunit exosome (EXO10) is formed by a catalytically inactive RNA-binding cage (EXO9) that channels the substrate to the 3′→5′ exoribonuclease subunit (Rrp44 in yeast and DIS3/DIS3L in the human nucleus/cytoplasm) (Weick et al., 2018; Gerlach et al., 2018; Makino et al., 2013; Tomecki et al., 2010; Dziembowski et al., 2007; Liu et al., 2006). EXO10 lacks substrate specificity but functions together with compartment-specific cofactors that contribute to the recognition and delivery of RNA substrates for degradation (reviewed in Schmid and Jensen, 2019; Butler and Mitchell, 2010). In the nuclear compartment, three cofactors (RRP6, RRP47, and MPP6) are tethered to EXO10 and form a lid near the entry to the RNA-binding cage (Falk et al., 2017a; Wasmuth et al., 2017; Zinder and Lima, 2017; Zinder et al., 2016; Makino et al., 2015; Wasmuth and Lima, 2012). These nuclear cofactors together recruit MTR4, a processive 3′→5′ RNA helicase (Falk et al., 2017a; Wasmuth et al., 2017; Schuch et al., 2014). In an active state, the complex adopts a channeling configuration, as MTR4 displaces the lid on top of EXO10 and threads RNA into the exosome degradation cage (Gerlach et al., 2018; Schuller et al., 2018; Weick et al., 2018).

In addition to its direct function in RNA channeling, MTR4 also orchestrates the outermost and diverse layers of nuclear exosome architecture by interacting with a variety of adaptor proteins (reviewed in Olsen and Johnson, 2021; Schmid and Jensen, 2019). The first adaptor complex to be identified was

This is an open access article under the CC BY license (http://creativecommons.org/licenses/by/4.0/).

the *S. cerevisiae* Trf4-Air2-Mtr4 polyadenylation (TRAMP) complex, which is responsible for substrate oligoadenylation prior to decay (LaCava et al., 2005; Vanácová et al., 2005; Wyers et al., 2005). More recently, the ribosomal biogenesis factor Nop53 was identified as an important adaptor for processing the 5.8S ribosomal RNA during the maturation of the large ribosomal subunit (Thoms et al., 2015). The mode of interaction with which Mtr4 recruits these adaptors varies: Trf4-Air2 binds the helicase domain (Falk et al., 2014) while Nop53 binds the so-called arch domain of Mtr4 (Falk et al., 2017b). In addition to the orthologues of the Trf4-Air2 and Nop53p, more MTR4-containing complexes have been identified in human cells (reviewed in Schmid and Jensen, 2019). Among these, the zinc-finger protein ZCCHC8 and the RNA-recognition motif (RRM)-containing protein RBM7 were discovered as prominent MTR4-interacting factors in a protein-protein interaction profiling study (Lubas et al., 2011). The resulting MTR4-ZCCHC8-RBM7 trimer, coined the nuclear exosome targeting (NEXT) complex, is now known to target a variety of non-coding RNAs to the nuclear exosome, including promoter upstream transcripts (PROMPTs) (Lubas et al., 2011), enhancer RNAs (eRNAs) (Meola et al., 2016), the 3′ extended products of sn(o)RNAs (Hrossova et al., 2015; Lubas et al., 2015), telomerase RNA (Tseng et al., 2015), and ncRNAs involved in the antibacterial immune response (Imamura et al., 2018). Overall, the NEXT complex has emerged as a major exosome adaptor for the degradation of non-functional transcripts arising from spurious transcription (Garland and Jensen, 2020).

The cellular functions of NEXT impact organismal physiology. In *Zcchc8* knockout mouse models, heterozygotes present defects in telomerase RNA metabolism, while homozygotes develop fatal neurodevelopmental pathologies linked to global dysregulation of the brain transcriptome (Gable et al., 2019). These findings relate back to disease mutations found in the human population. A heterozygous loss-of-function mutation in ZCCHC8 has been identified as the cause of a syndrome related to telomerase RNA maturation (Gable et al., 2019). In addition, a homozygous mutation in the NEXT RBM7 subunit has been linked to clinical defects in motor neurons and the cerebellum (Giunta et al., 2016). Despite the important roles of the nuclear exosome and NEXT for cellular function and organismal physiology, their underlying molecular mechanisms remain unclear. Biochemical and structural studies have investigated selected protein-protein interactions in the NEXT complex (Lingaraju et al., 2019a; Puno and Lima, 2018; Falk et al., 2016), but the overall architecture and the principles with which it guides substrates to exosome-mediated degradation are unknown.

In this work, we therefore set out to obtain molecular insights into these questions by combining cryo-EM analyses with biochemical studies and cell-based assays. In terms of architecture, we found that the biological unit of NEXT consists of two copies of each MTR4 and RBM7, assembling symmetrically around a highly intertwined ZCCHC8 homodimer, with an arrangement conducive for targeting different RNA substrates. In terms of its connections to the RNA decay machinery, we found that NEXT contains a gatekeeping feature that regulates both the exit of the RNA substrates from this helicase-containing complex and the entry into the ribonuclease-containing exosome.

RESULTS AND DISCUSSION

The NEXT complex has a homodimeric structure

The subunits of the NEXT complex have a multidomain organization comprised of folded domains and unstructured regions (Figure 1A). Previous biochemical studies performed by us and others have shown that the removal of unstructured regions from RBM7 and ZCCHC8 improved the homogeneity of recombinant samples (Lingaraju et al., 2019a; Puno and Lima, 2018; Falk et al., 2016). These findings guided our decision to structurally characterize two versions of NEXT using single-particle cryo-EM (Figure S1). The smaller version of NEXT (defined as NEXT^S) consisted of full-length MTR4, a truncated mutant of RBM7 containing the RRM domain (residues 1–98, for simplicity referred to as RBM7; Falk et al., 2016), and a truncated mutant of ZCCHC8 in which only the minimal MTR4- and RBM7-binding regions remained (residues 41–337, referred to as ZCCHC8^S; Falk et al., 2016) (Figures S1A–S1D). The larger version of the NEXT complex (defined as NEXT^L) contained full-length MTR4, RBM7 as defined above, and a deletion mutant of ZCCHC8 lacking an unstructured segment in the C-terminalhalf of the protein but still including the C-terminal domain (Puno and Lima, 2018) (Δ416-506 deletion, referred to as ZCCHC8^L) (Figures S1E–S1H).

The NEXT^S and NEXT^L complexes were both incubated with the ATP analog AMPPNP and with poly-uridine-rich RNAs longer than 20 nucleotides to accommodate the established RNA-binding properties of NEXT (Puno and Lima, 2018). Cryo-EM structural analyses of these complexes yielded 3D reconstructions at various degrees of resolution (Figures S1D and S1H) that allowed the tracing of portions of polypeptide chains *de novo* or the fitting of high-resolution information from previous experimental crystal structures (Puno and Lima, 2018; Falk et al., 2016) or from artificial intelligence (AI) predictions by AlphaFold (Jumper et al., 2021; Tunyasuvunakool et al., 2021). In general, the NEXT^S reconstruction allowed us to obtain a model of the inner core of the complex, and the NEXT^L reconstruction was used to obtain a model of the outer regions of the complex and of the ribonucleotide chain. The cryo-EM, X-ray crystallography, and AI-based information on both reconstructions were integrated to obtain a composite model of the NEXT homodimer (Figures 1B and S1I).

As this composite model represents the most complete view of the NEXT complex that we have achieved in this work, we will first orient the reader by presenting the overall architecture before proceeding, in subsequent sections, to discuss the structural and biochemical data leading to this model. Briefly, NEXT forms a homodimer built around the N-terminal region of two ZCCHC8 protomers, which intertwine to form a coiled coil and a globular dimerization module. The ZCCHC8 dimerization module interacts in a symmetric manner with two MTR4 protomers via their arch domains, in turn positioning the DExH-box helicase domains in opposing orientations with respect to each other. Altogether, this arrangement constitutes the inner core of the complex, which is flanked by the more flexible domains of ZCCHC8 and RBM7 that are known to contribute to RNA-binding and/or ATPase activity (Puno and Lima, 2018; Falk et al., 2016). For simplicity of representation, the composite model in Figure 1B shows these additional domains arranged in a symmetric manner around the DExH-box domain. Nevertheless,

CellPress
OPEN ACCESS

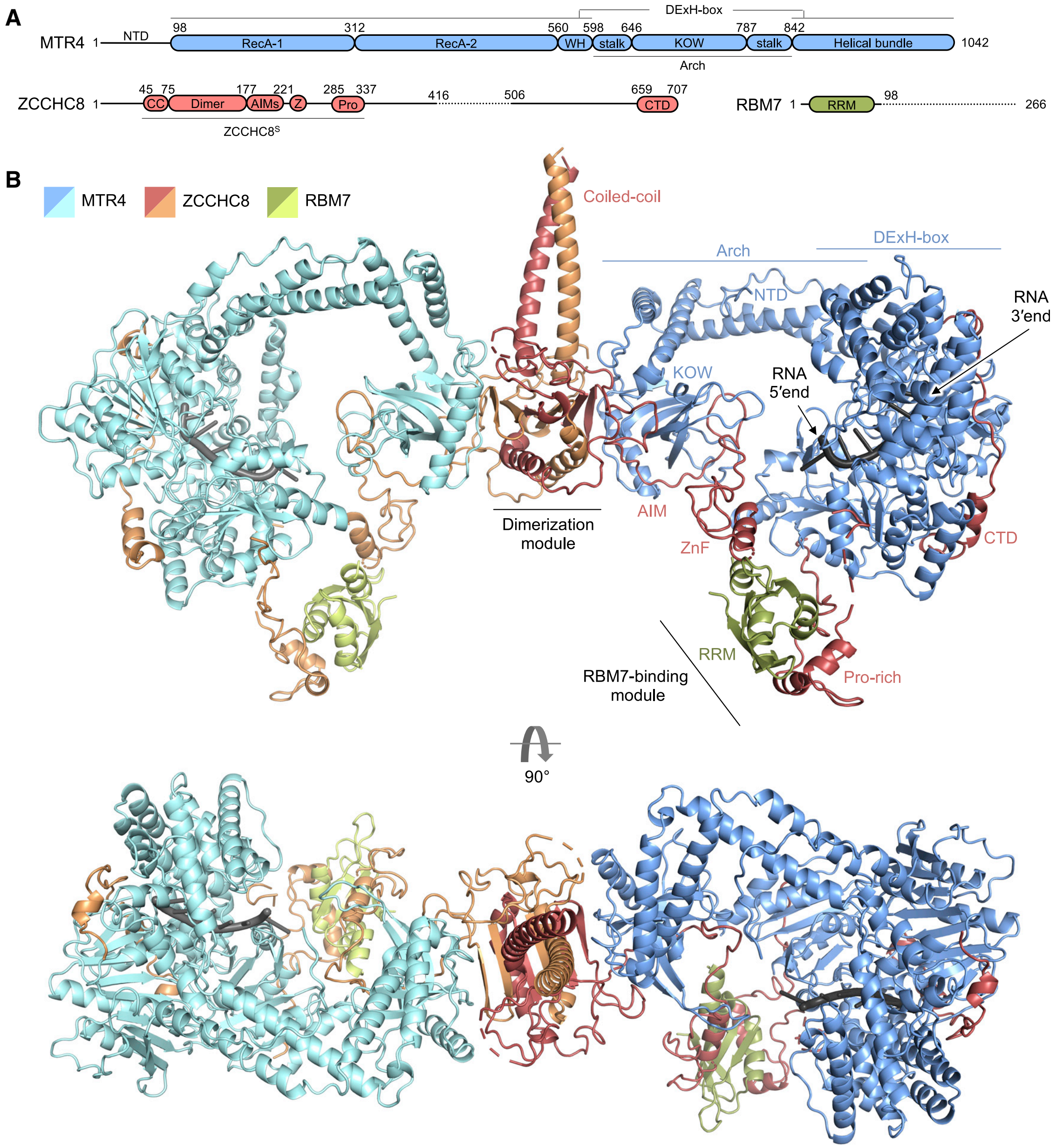

Figure 1. Composite model of the NEXT homodimer structured core

(A) Domain organization of the NEXT complex subunits MTR4, ZCCHC8, and RBM7. Domain boundaries correspond to the structural data. Dotted lines correspond to unstructured regions that were deleted in the NEXT^L sample. Smaller ZCCHC8^S construct used in the NEXT^S reconstruction is highlighted. Abbreviations: WH, winged helix; KOW, Kyrpides-Ouzounis-Woese; NTD, N-terminal domain; CC, coiled coil; Z, Zinc finger; AIMs, arch-interacting motifs; Pro, Pro-rich domain; CTD, C-terminal domain; RRM, RNA-recognition motif.

(B) Composite structural model of the NEXT homodimer obtained from interpreting cryo-EM reconstructions of the NEXT^S and NEXT^L samples with *de novo* model building and with rigid-body fitting of available crystal structures or AlphaFold predictions. Labels are indicated only for one protomer.

due to the inherent flexibility in this portion of the complex, the details of this arrangement must be viewed with caution.

The inner core of NEXT contains two MTR4 helicases in a head-to-head configuration

The cryo-EM analysis of the NEXT^S complex resulted in a reconstruction with a global resolution of 4.5 Å (Figures 2A and S1A–S1D). The map showed ordered density for two MTR4 protomers (referred to as MTR4$_A$ and MTR4$_B$) facing each other in a head-to-head configuration. This density was separately fitted with the atomic models of the MTR4 DExH-box domain and arch domain, as the latter has different conformations in the available crystal structures (Lingaraju et al., 2019a; Wang et al., 2019; Puno and Lima, 2018) (Figure 2A).

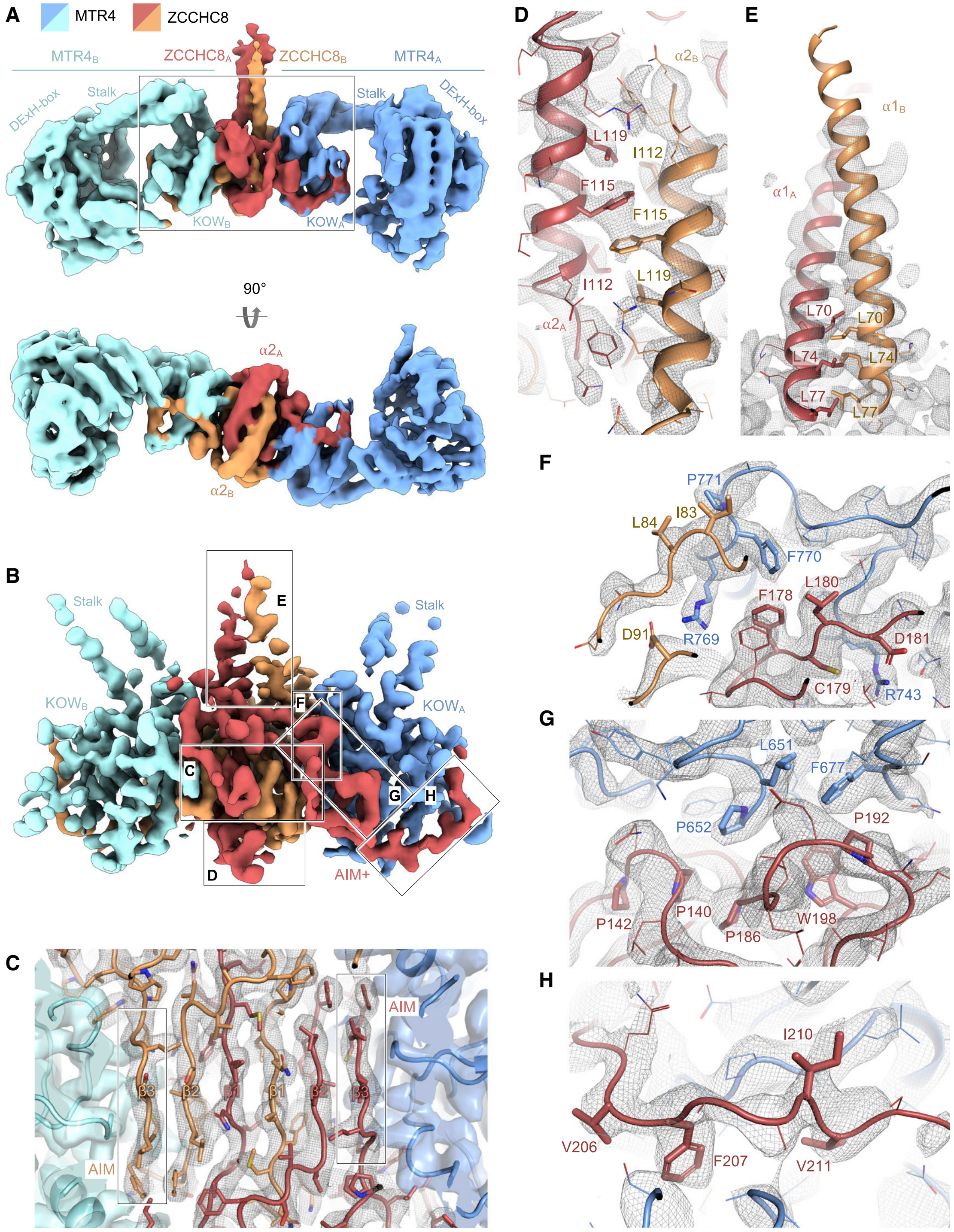

Figure 2. Interactions at the ZCCHC8 dimerization and MTR4-binding interfaces

(A) Single-particle cryo-EM reconstruction of NEXT^S at a global resolution of 4.5 Å, low-pass filtered to 5 Å, shown in two views, with density colored according to Figure 1B. The two protomers of ZCCHC8 and MTR4 are labeled as A and B. The box indicates the area used for the focused refinement shown in (B).

(B) 4.0 Å resolution cryo-EM reconstruction after focused refinement at the dimerization module sandwiched between MTR4 KOW domains, followed by density modification (Phenix). The boxes highlight the regions in the zoom-in views of (C)–(H) where the ZCCHC8 model could be built *de novo* (with [C], [D], and [F] shown after an ~90° rotation).

(C) Zoom-in of the reconstruction at the dimerization module showing the density at the β-sheet with its domain-swapping topology.

(legend continued on next page)

CellPress
OPEN ACCESS

The DExH-box domain has the characteristic architecture, with two RecA folds (RecA1 and RecA2) juxtaposed to a helical domain formed by a winged helix and a helical bundle. The arch domain is inserted in this helical domain and forms a protrusion with a helical stalk ending in a globular Kyrpides-Ouzounis-Woese (KOW) domain (Figures 1B and 2A) (Olsen and Johnson, 2021). Previous studies have shown that the MTR4 arch domain can adopt inward or outward conformations (reviewed in Olsen and Johnson, 2021). In our reconstruction of NEXT^S, the MTR4 arch domain of both protomers is arranged in an inward conformation, similar to that observed in apo human MTR4 (Wang et al., 2019) and apo yeast Mtr4 (Weir et al., 2010) (Figure S2A). In this conformation, the stalk of the arch is bent inward toward the DExH-box while the KOW domain is close to RecA2 and the entry of the helicase channel (Lingaraju et al., 2019a; Wang et al., 2019; Weir et al., 2010; Jackson et al., 2010).

The N-terminal region of ZCCHC8 mediates homodimerization and MTR4-binding

The KOW domains of the two MTR4 helicases sandwich a globular density that is, in turn, connected to an elongated protrusion (Figure 2A). In biochemical assays, the KOW domain of MTR4 has been shown to bind an N-terminal region of ZCCHC8 (Lingaraju et al., 2019a). In the absence of an experimental structure or a reliable prediction for this region of ZCCHC8, we focused the cryo-EM processing to increase the local resolution in this area of the complex (box in Figure 2A). Three-dimensional classification followed by 3D refinement and density modification (Terwilliger et al., 2020) led to a reconstruction at a nominal resolution of 4.0 Å, but with a map quality sufficient to trace the ZCCHC8 globular module *de novo* (Figures 2B, S1D, and S2B–S22I). This module is formed by the interaction of two ZCCHC8 protomers (referred to as ZCCHC8$_A$ and ZCCHC8$_B$), involving their respective dimerization segments (residues 78–198). The dimerization segments of ZCCHC8 intertwine to form a central six-stranded β-sheet characterized by domain swapping: the first β-strands are exchanged between the two ZCCHC8 protomers, resulting in a $\beta3_A$-$\beta2_A$-$\beta1_B$-$\beta1_A$-$\beta2_B$-$\beta3_B$ topology of secondary structure elements (Figure 2C). Beneath the β-sheet, two α helices from the ZCCHC8 protomers ($\alpha2_A$ and $\alpha2_B$), positioned below the corresponding β-strands ($\beta1_A$ and $\beta1_B$), pack against each other in an antiparallel fashion with conserved hydrophobic interactions (Phe115 and Leu119) (Figure 2D).

Above the β-sheet, the helices at the N terminus of the dimerization segments of the two protomers ($\alpha1_A$ and $\alpha1_B$) protrude to form a parallel coiled-coil oriented in a roughly perpendicular fashion with respect to the ZCCHC8 dimerization module. The focused map showed the hydrophobic interactions at the end of the $\alpha1_A$ and $\alpha1_B$ helices (Figure 2E). In turn, this allowed us to fix the register and fit the rest of the coiled coil (modeled using AlphaFold; Jumper et al., 2021; Tunyasuvunakool et al., 2021) in the density of the original 4.5 Å resolution map of NEXT^S (Figures 2A and 2E).

ZCCHC8 and the MTR4 KOW domain engage with extended interaction surfaces

The N-terminal region of ZCCHC8 displays an approximate two-fold rotational symmetry, with a two-fold axis set along the coiled coil and through the middle of the globular dimerization module (Figures 1B and 2A). The ZCCHC8 homodimer indeed binds the two MTR4 KOW domains in a symmetric manner; therefore, only one of the protomers will be described below. The MTR4 KOW domain has a well-characterized fold, with a β-barrel containing two long loops and a prominent α helix (Jackson et al., 2010; Wang et al., 2019; Weir et al., 2010). The KOW$_A$ domain interacts extensively with ZCCHC8$_A$ and also in part with ZCCHC8$_B$ (Figure 2B). A major interaction is centered at the canonical arch-interacting motif (AIM) of ZCCHC8$_A$ (residues 178–184), consistent with biochemical mapping experiments (Lingaraju et al., 2019a). This AIM contains a cysteine residue (Cys179, hence referred to as C-AIM in Lingaraju et al., 2019a) that we found is involved in hydrophobic interactions within the ZCCHC8 dimerization module. The ZCCHC8 AIM is indeed embedded in strand β3 of the dimerization module (Figure 2C) and, as such, it appears to be pre-configured in the β-strand conformation with which canonical AIMs are known to recognize the MTR4 KOW domain (Figure S2N) (Falk et al., 2017b). In addition, the N terminus of the ZCCHC8$_A$ AIM binds a loop of KOW$_A$ via hydrophobic contacts (MTR4$_A$ Phe770 contacting ZCCHC8$_A$ Phe178 and Ile180), in turn positioning this loop to interact with ZCCHC8$_B$ (Figure 2F).

At the C terminus of the ZCCHC8$_A$ AIM, the polypeptide chain makes a 90° bend (residues 185–199). This region is stabilized by a striking array of intramolecular proline-aromatic interactions (Pro142-Pro140-Pro186-Trp198-Pro192; Figure 2G), resulting in a "bulge" that contacts a hydrophobic patch on KOW$_A$ (MTR4$_A$ Pro652, Leu651, and Phe677). After the bulge, ZCCHC8$_A$ (residues 200–220) continues to wrap around the KOW$_A$ domain with several hydrophobic interactions (Figure 2H), following a similar binding path to that of the MTR4-binding protein NRDE2 (Figure S2O) (Wang et al., 2019). Interestingly, ZCCHC8 and NRDE2 share similar residues within this MTR4-binding region (Figure S2O). Thus, ZCCHC8 and NRDE2 appear to have an arch-interacting domain that is 35 residues longer than other known MTR4-binding proteins (Figure S2N). We will refer to this additional arch-interacting region as AIM+ (ZCCHC8 residues 185–220) (Figures 2B and S2O). The structural analysis explains the effect of mutations that have been previously shown to impair NEXT complex formation (Lingaraju et al., 2019a) and also rationalizes the impact of a disease-associated mutation, P186L (Gable et al., 2019), that we predict would destabilize the proline-aromatic array at the "bulge" in the AIM+ region of ZCCHC8.

Monomerized NEXT is sufficient to target some RNAs for degradation

To dissect the contribution of the different structural features observed in the NEXT^S cryo-EM reconstruction, we engineered

(D) Zoom-in of the interaction between antiparallel helices α2 at the bottom of the dimerization module.
(E) Zoom-in of the interaction between helices α1 forming the N-terminal coiled coil. Lower map threshold was applied (PyMOL) compared with other panels, to better visualize density for the helices.
(F–H) Zoom-in view at different sites of ZCCHC8-MTR4 KOW interactions: at the classical AIM (F), at the bulge (G), and at the AIM+ (H).

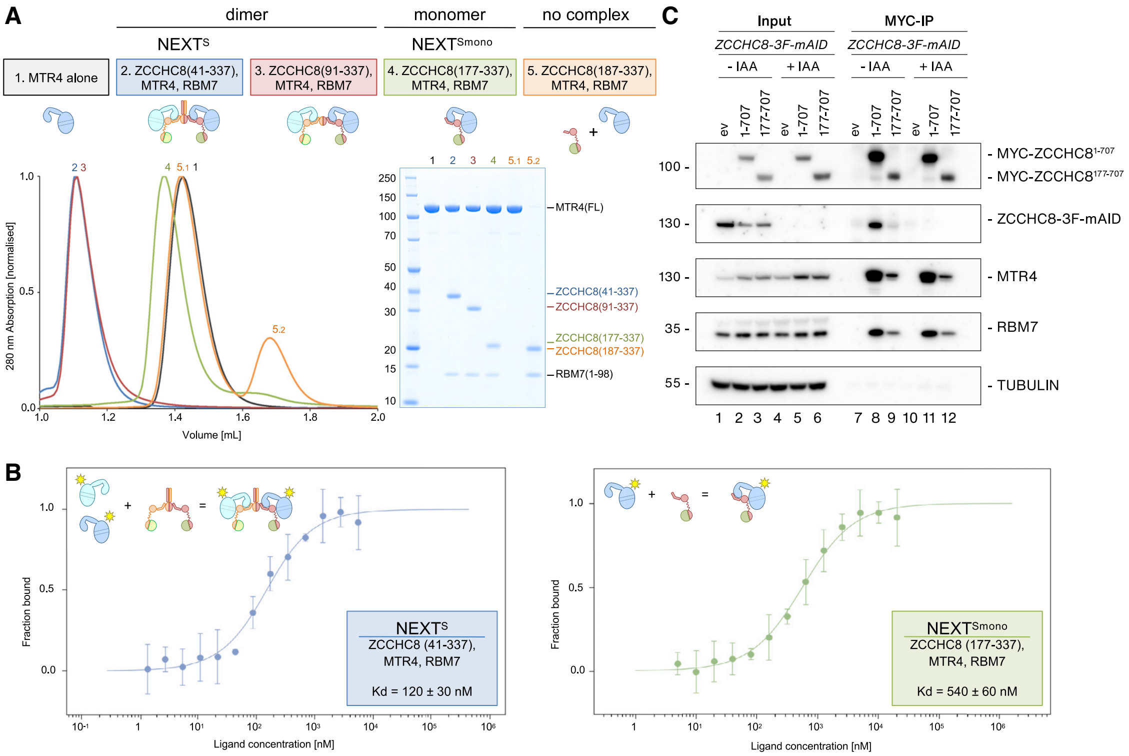

Figure 3. Monomeric form of ZCCHC8 supports MTR4-RBM7 association

(A) Biochemical analysis to identify the major determinants of ZCCHC8 dimerization and MTR4-binding properties *in vitro*. The recombinant protein samples indicated at the top were subjected to size-exclusion chromatography experiments (analytical S200i, bottom left) and the peak fractions analyzed on a Coomassie-stained 4%–12% bis-Tris SDS-PAGE (bottom right). Deletion of the N-terminal 176 residues resulted in a monomeric NEXT mutant.

(B) Biophysical analysis to quantify the contribution of ZCCHC8 dimerization on MTR4-binding. The microscale thermophoresis experiment was carried out by keeping eYFP-tagged MTR4 at a fixed concentration (50 nM) and adding increasing amounts of dimeric or monomeric ZCCHC8 and RBM7. Titrations were performed in triplicate and error bars represent standard deviation. Fitting of the experimental data curve and estimation of the dissociation constant (K_D) was done with the MO software (NanoTemper technologies).

(C) Cell-based analysis to assess the presence of ZCCHC8 oligomerization *in vivo* and its effect on MTR4-RBM7 binding. Co-immunoprecipitation experiments were carried out with exogenous MYC-tagged versions of ZCCHC8 (ev, empty vector) to assess the interaction with endogenous ZCCHC8-3F-mAID in the presence or absence of auxin (IAA) treatment. The experiment showed that full-length ZCCHC8 (residues 1–707) can interact with endogenous ZCCHC8-3F-mAID, while a mutant lacking the N-terminal 176 residues fails to dimerize but can still co-precipitate MTR4 and RBM7.

a series of structure-based mutants of ZCCHC8 and evaluated their interaction properties with recombinant MTR4 and RBM7 by size-exclusion chromatography (Figure 3A). As a control, the NEXT^S homodimer eluted at a significantly earlier time point than MTR4 in isolation (Figure 3A; compare samples 1 and 2). A truncated version of the complex reconstituted with ZCCHC8 residues 91–337 (designed to remove the coiled coil) eluted at the same volume as NEXT^S (Figure 3A; compare samples 2 and 3). In contrast, an increasingly truncated version of the complex containing ZCCHC8 residues 177–337 (designed to remove most of the dimerization module, except for the AIM at β3) behaved as a monomeric mutant form of the complex (referred to as NEXT^{Smono}), eluting after NEXT^S and just before MTR4 (Figure 3A; compare samples 4, 2, and 1). Even when ZCCHC8 was further truncated to include only residues 187–337 (i.e., the AIM was removed), it still bound RBM7 but failed to bind MTR4 (Figure 3A; sample 5). These results allowed us to draw two conclusions. First, while the globular ZCCHC8 dimerization module is required for NEXT oligomerization, the coiled coil is dispensable. Second, while the ZCCHC8 AIM is essential to establish binding with MTR4, the AIM+ is less critical. Of note, the presence of MTR4 did not influence the oligomerization properties of ZCCHC8; ZCCHC8 41–337 behaved as a dimer and ZCCHC8 177–337 as a monomer in size-exclusion chromatography experiments when RBM7 was present and MTR4 was absent (Figure S3A). Conversely, we measured a small, albeit reproducible, difference in the affinities with which dimeric and monomeric ZCCHC8-RBM7 units interact with MTR4, with the dimer showing a stronger affinity (Figure 3B).

To probe for NEXT dimerization capabilities *in vivo*, we tagged endogenous ZCCHC8 loci with 3xFLAG and mini auxin-inducible degradation (mAID) epitopes (Natsume and Kanemaki, 2017; Nishimura et al., 2009) (ZCCHC8-3F-mAID) in OsTIR1-expressing HeLa cells using CRISPR-Cas9. Additionally, we used the piggyBac transposon system (Ding et al., 2005) to integrate MYC-tagged ZCCHC8$^{1–707}$ and ZCCHC8$^{177–707}$ cDNA

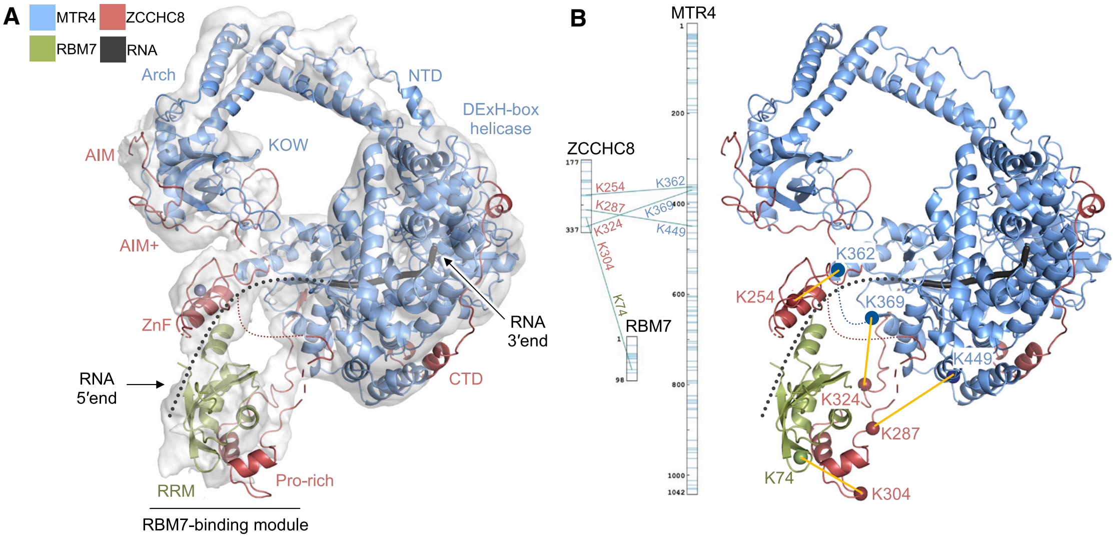

Figure 4. The RNA-binding domains of ZCCHC8 and RBM7 are adjacent to the MTR4 helicase domain

(A) Single-particle cryo-EM reconstruction focused on a single NEXT^L protomer at a global resolution of ~7 Å. Cryo-EM density is depicted as a gray transparent surface and the composite model is shown as a ribbon. The model of the KOW domain with the ZCCHC8 AIM and AIM+ regions is from the *de novo* tracing from the NEXT^S reconstruction (Figure 2). The model of the MTR4 DExH-box domain bound to the ZCCHC8 CTD (PDB: 6C90) (Puno and Lima, 2018) could be placed as a rigid body in the density. The model of the ZCCHC8 zinc-finger from AlphaFold and the crystal structure of the RBM7-binding module (Falk et al., 2016) (PDB: 5LXR) could be tentatively placed in the density as rigid bodies with an orientation fulfilling the connecting density features and the mass spectrometry cross-linking data (B). Other small density features at the MTR4 arch and DExH-box domains could be interpreted by predictions of the corresponding regions with AlphaFold (see also Figure S4).

(B) Mass spectrometry analysis of the BS3-cross-linked monomeric mutant NEXT^S (i.e., containing ZCCHC8^S, residues 177–337; Figure 3) in the presence of the uridine-rich RNA and AMPPNP. Identified Lys-Lys cross-links are highlighted on the diagram and cartoon representation of the complex.

constructs; the latter introduced the N-terminal monomerizing truncation. Pull-down experiments were carried out by immunoprecipitating MYC-tagged ZCCHC8$^{1–707}$ and ZCCHC8$^{177–707}$ proteins in either mock cells or upon treatment with auxin (indole-3-acetic acid, IAA) to conditionally deplete endogenous ZCCHC8-3F-mAID. Despite differences in pull-down efficiencies, both MYC-tagged ZCCHC8$^{1–707}$ and ZCCHC8$^{177–707}$ proteins relatively co-immunoprecipitated MTR4 as well as RBM7 in both −IAA and +IAA conditions (Figure 3C; compare lanes 11 and 12), suggesting that a mutant monomeric NEXT complex could be formed. However, only MYC-tagged ZCCHC8$^{1–707}$, not the truncated ZCCHC8$^{177–707}$ mutant, co-immunoprecipitated endogenous ZCCHC8-3F-mAID, demonstrating that ZCCHC8 also dimerizes *in vivo* and that this is mediated by the ZCCHC8 N-terminal region (Figure 3C; compare lanes 8 and 9). We took advantage of this conditional system to interrogate any function of such a complex by analyzing levels of known NEXT targets, including PROMPTs, 3′ extended U11 snRNA, and 3′ extended intronic-hosted SNORD83a snoRNA. Upon depletion of ZCCHC8-3F-mAID in control cells complemented with an empty vector (ev), all tested NEXT targets were considerably upregulated (Figure S3B). Conversely, when the same cells were complemented with either ZCCHC8$^{1–707}$ or ZCCHC8$^{177–707}$, NEXT target levels equaled those in cultures where ZCCHC8-3F-mAID was not depleted (−IAA) (Figure S3B). With the caveat that the exogenous variants of NEXT are somewhat overexpressed in these experiments, we conclude that NEXT dimerization is not required for the decay of the tested RNAs.

RNA-binding domains in ZCCHC8 and RBM7 flank the RNA-entry channel of MTR4

The cryo-EM analysis of NEXT^S allowed visualization of the interaction between the ZCCHC8 N-terminal dimerization module and the MTR4 KOW domain but failed to reveal other features of the complex (e.g., the RBM7 binding site), most likely due to their inherent flexibility (Figure 2A). Upon analyzing the cryo-EM data collected on NEXT^L, we noticed that the 2D classes showed additional densities in proximity to an MTR4 helicase (Figure S1G). Focusing the data processing on the NEXT^L protomer with these additional densities, we obtained a cryo-EM reconstruction that refined to a global resolution of ~7 Å (Figures 4A and S1E–S1H). In this reconstruction, a small globular density appeared below the MTR4 KOW domain and a larger globular density at the side of the MTR4 DExH-box domain. From their position, size, and connectivity to other parts of the map (see below), we interpreted the smaller density as corresponding to the zinc-finger domain of ZCCHC8 (residues 228–243) (modeled from a high-confidence prediction by AlphaFold; Tunyasuvunakool et al., 2021) and the larger density as corresponding to the RBM7-binding module (known from crystallographic studies to consist of RBM7 and the Pro-rich domain of ZCCHC8, residues 285–324; Falk et al., 2016) (Figure 4A).

Since it was not possible to precisely orient the zinc-finger domain and the RBM7-binding module in the 7 Å resolution density, we introduced additional restraints using cross-linking mass spectrometry experiments. To simplify the read-out

expected from these types of experiments, we used the monomeric NEXT^{Smono} complex. When the sample was incubated with RNA/AMPPNP, we measured a distinct set of cross links that we then used to orient the ZCCHC8 zinc-finger domain and the RBM7-binding module as rigid bodies in the cryo-EM density (Figure 4B). This interpretation was independently supported by the presence of additional density features. In particular, the map showed a density accounting for the short linker sequence between the AIM+ and the zinc-finger domain (Figure S4A), another density pointing from the zinc-finger domain toward the conserved acidic pocket of the MTR4 RecA2 domain (Figure S4B), and a tubular density re-connecting the ZCCHC8 Pro-rich domain again to the side of the MTR4 RecA2 domain (Figure S4C). In addition, guided by corresponding features of the human NRDE2 (Wang et al., 2019) and the yeast Trf4 and Air2 (Falk et al., 2014), we proposed how to fit two short motifs of the ZCCHC8 protein within these densities (Figures S4D–S4E). Although this interpretation remains tentative, as the resolution of the map is not sufficient to visualize individual residues, it is consistent with the available information and allows us to propose a comprehensive model for the architecture of this portion of the complex. In this model, the RNA-binding β-sheet surface of the RBM7 RRM domain is exposed to solvent, angled toward the ZCCHC8 zinc-finger domain, and lies adjacent to the RNA-entry site of the DExH-box domain. Together, these small globular domains would thus be appropriately positioned to direct an incoming RNA substrate toward the RNA channel of MTR4.

The ZCCHC8 C-terminal domain forms a lid below the RNA exit channel

The 7 Å resolution cryo-EM map of the NEXT^{L} sample also revealed additional density features near and/or within the expected RNA-binding site of the DExH-box domain. To better resolve this portion of the complex, we focused the cryo-EM processing solely on the MTR4 DExH-box domain, allowing us to obtain a reconstruction with a local resolution of 3.4 Å (Figures S1H and S5A–S5D). The resulting cryo-EM reconstruction, inspected at low map threshold, showed density at the bottom of the DExH-box domain consistent with the presence of the ZCCHC8 C-terminal domain (CTD, residues 659–700) as observed in the corresponding crystal structure (Puno and Lima, 2018) (Figure 5A). Briefly, the ZCCHC8 CTD binds along the bottom of the DExH-box domain, starting from RecA1 and ending at RecA2, in the proximity of AMPPNP (Figures 5A and 5B). No density was visible for the region directly upstream of the ZCCHC8 CTD (residues 339–415 and 507–658 in the ZCCHC8^{L} construct), which is indeed predicted to be mostly unstructured. However, additional density was present inside the helicase channel of MTR4. The MTR4 helicase channel is known to bind RNA in a defined polarity, with the 5′ and 3′ ends near the top and bottom surfaces, respectively, of the DExH-box domain (Gerlach et al., 2018; Weick et al., 2018; Weir et al., 2010). Starting from the top surface of the DExH-box domain, the cryo-EM map showed well defined density for the first four nucleotides and less resolved density for the fifth one. The last nucleotide is 14 Å away from the end of the helicase channel, where the ZCCHC8 CTD resides (Figure 5C).

The structural analysis suggested that the ZCCHC8 CTD encloses the RNA by gating the exit of the RNA channel—similar to a gatekeeping feature we recently identified in the cytoplasmic cofactor of the exosome (Kögel et al., 2022). To validate this observation, we carried out RNase protection assays using established protocols for characterizing the RNA-binding properties of exosome complexes (Gerlach et al., 2018; Bonneau et al., 2009). We added a radioactively body-labeled RNA and ADP-BeFx to MTR4 in isolation or in complex with different versions of ZCCHC8-RBM7 and incubated the samples with benzonase. Analysis of the RNA-protected fragments on denaturing polyacrylamide gel electrophoresis demonstrated different RNA-binding footprints. As expected, no accumulated fragments were observed in the case of MTR4 in isolation (Figure 5D, lane 1). In contrast, the NEXT^{L} complex gave rise to a defined protection pattern with the accumulation of RNA fragments centered at ∼18 nucleotides (Figure 5D, lane 2), in agreement with the RNA-binding properties of NEXT, as determined by cross-linking assays (Puno and Lima, 2018). The NEXT^{S} complex yielded a less defined protection pattern with a longer and rather smeared ladder of RNA fragments (Figure 5D, lane 3). A similar footprint to that in NEXT^{S} was observed for NEXT^{Smono} (Figure 5D, lane 4), suggesting that dimerization does not impact the RNA-binding properties of the individual protomers. The NEXT^{S} and NEXT^{Smono} protection patterns can be explained by the lack of precision of benzonase endonuclease activity at both ends of the RNA, which is exposed on either side of the helicase channel in the absence of the ZCCHC8 CTD. Thus, the result is a broader peak distribution of RNA fragment sizes as compared with NEXT^{L}. Importantly, the addition of separately purified ZCCHC8 CTD *in trans* to both NEXT^{S} and NEXT^{Smono} recapitulated the compact footprint of NEXT^{L}, supporting the notion that the ZCCHC8 CTD restricts benzonase access to RNA substrates by gating one side of the RNA-binding path (scheme in Figure 5D). With hindsight, the ability of the ZCCHC8 CTD to lock the RNA substrate in the helicase channel rationalizes why we could observe density for RBM7 in the NEXT^{L} reconstruction but not in in the NEXT^{S} reconstruction, as the positioning of RBM7 near the MTR4 DExH-box domain is likely stabilized by RNA binding.

Cryo-EM structure of a NEXT-exosome assembly

Superpositions with MTR4-exosome complexes (Gerlach et al., 2018; Weick et al., 2018) suggested that, in the gating position, the ZCCHC8 CTD would sterically clash against the EXO10 cap protein RRP4 and the exosome nuclear factor MPP6 (Figures S5E–S5H). Thus, a prediction from the structural analysis is that the ZCCHC8 CTD would have to be removed from its gating position for the RNA 3′ end to exit the helicase channel and enter the exosome core complex. To validate this prediction, we carried out competition experiments with recombinant proteins. We purified a human nuclear exosome complex, using a strategy that we previously developed to determine how it recruits MTR4 in a stable RNA-channeling conformation (Gerlach et al., 2018). Briefly, we covalently linked two nuclear cofactors (MPP6 and the N-terminal domain of RRP6, RRP6^{N}) to two cap proteins of EXO9 and included the nuclear cofactor RRP47 to obtain a stable EXO9-MPP6-RRP6^{N}-RRP47 complex (Figure S6H). As previously, the

CellPress
OPEN ACCESS

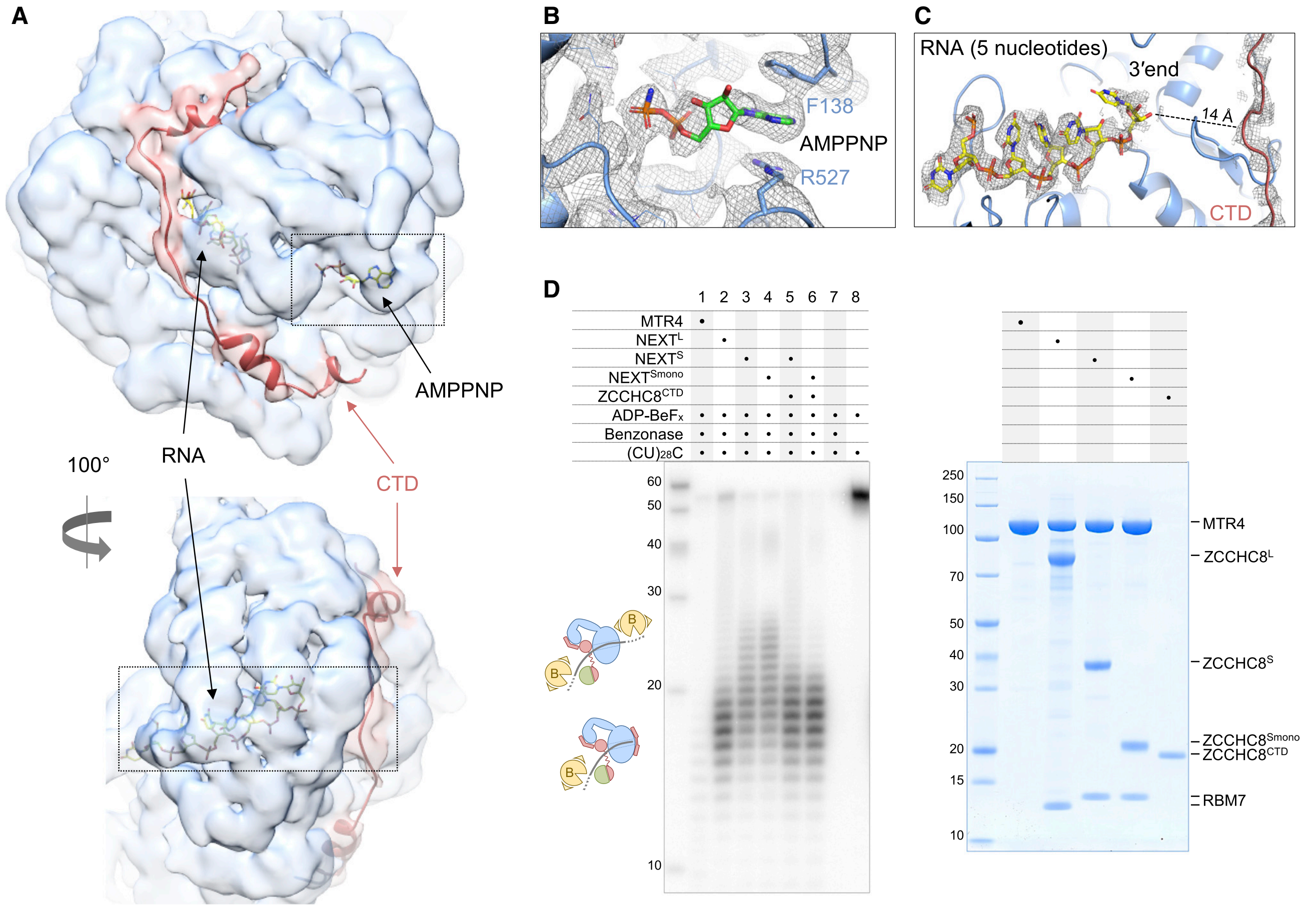

Figure 5. RNA is enclosed in the MTR4 helicase channel when NEXT is not active

(A) Cryo-EM reconstruction from the NEXT^L sample focused at the DExH-box helicase domain of MTR4. In the two views, the cryo-EM map is shown as a transparent surface at a low map threshold (Chimera) to highlight the density features at the bottom of the DExH-box domain, that could be interpreted by rigid-body fitting the corresponding crystal structure bound to the ZCCHC8 CTD (PDB: 6C90) (Puno and Lima, 2018).

(B) Zoom-in view of the density of the ATP analog, AMPPNP, bound in the MTR4 ATPase site. The same cryo-EM reconstruction as above (reaching a nominal resolution of 3.4 Å) represented as mesh at a higher map threshold (PyMOL) to reveal nucleotide and amino acid details.

(C) Zoom-in view of the density in the RNA-binding channel of MTR4. Meshed experimental density, at the same map threshold as in (B), surrounds 5 ribonucleotides (yellow sticks) and ZCCHC8 CTD (red ribbon).

(D) RNase protection assay showing the RNA fragments obtained upon RNase treatment of the ^{32}P body-labeled (CU)$_{28}$C 57-mer RNA in the presence of the indicated protein complexes. After incubation with benzonase, the reactions' products were analyzed by electrophoresis on a 12% acrylamide and 7 M urea gel, followed by phosphorimaging. Right side of the panel shows a Coomassie-stained 4%–12% bis-Tris SDS-PAGE with complexes used in the assay. The cartoon schematics depict how benzonase, an endonuclease, might access the RNA in our hypothesized model, resulting in a short or long footprint.

catalytic domain of RRP6 was omitted from the complex because it competes with MTR4 for the EXO9-binding site (Gerlach et al., 2018). We incubated this nuclear exosome complex with the NEXT^S homodimer in the absence or presence of ZCCHC8 CTD *in trans*. Size-exclusion chromatography assays showed that the ZCCHC8 CTD was, indeed, largely competed out upon the binding of NEXT^S to the nuclear exosome, even in the absence of RNA (Figure S6H).

To study the interaction of NEXT with the nuclear exosome in the context of RNA, we incubated an EXO10-MPP6-RRP6^N-RRP47 complex (EXO13) with NEXT^S, a 60-nucleotide uridine-rich RNA and AMPPNP (Figure 6A). Upon cryo-EM data collection and processing, we identified particles representing homodimeric NEXT complexes engaged with one or two exosomes (Figures 6B and S6B–S6G). Further processing allowed us to obtain a ~10 Å resolution reconstruction corresponding to a NEXT^S homodimer and one exosome complex (Figures 6C and S6D). Density corresponding to the dimerization module of ZCCHC8 bound by two MTR4 helicases was fitted with the corresponding atomic model from the NEXT^S cryo-EM reconstruction. In one of the NEXT^S protomers, the bottom of the DExH-box domain of MTR4$_A$ presented a large additional density that was interpreted by fitting the exosome core. Upon superposing the MTR4$_A$ subunit of NEXT^S with the corresponding subunit of the EXO13-MTR4 structure (Gerlach et al., 2018; Weick et al., 2018), the EXO13 coordinates fitted in the remaining density with essentially no manual intervention, both for the exosome core and for the binding of RRP6^N-RRP47 at the stalk of

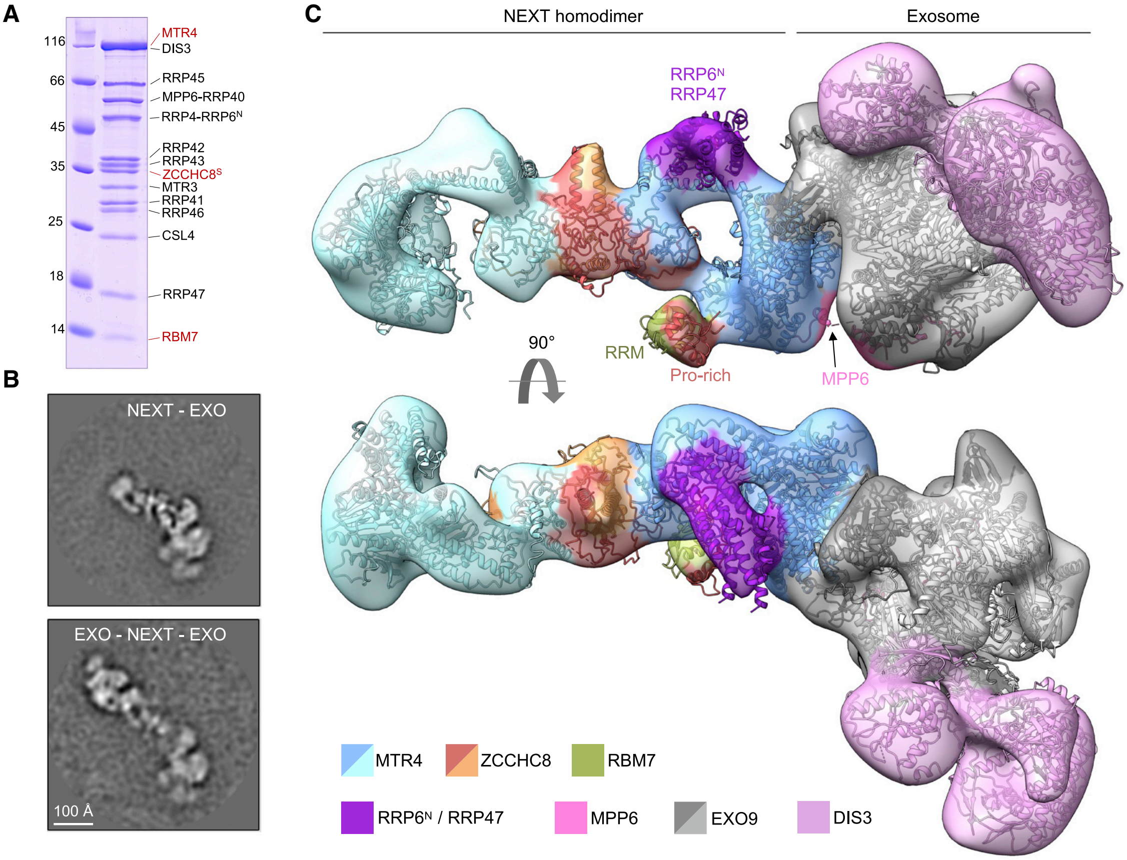

Figure 6. Cryo-EM reconstruction of a NEXT-exosome assembly

(A) Coomassie-stained 15% SDS-PAGE gel with peak fraction from size-exclusion chromatography of a reconstituted NEXT^S-exosome complex. The three NEXT^S complex components are labeled in red. Two of the nuclear exosome cofactors, MPP6 and RRP6^N, are fused to the EXO9 subunits RRP40 and RRP4, respectively (Gerlach et al., 2018).

(B) Representative 2D classes of the dimeric NEXT^S complex interacting with either one or two exosome complexes.

(C) Best 3D class representing the NEXT^S dimer bound to a single exosome. The cryo-EM map is shown as a transparent surface, colored according to the protein models rigid-body fitted in the density. The RBM7-binding module was fitted as in Figure 4A and the RRP6^N/RRP47 module was fitted based on Gerlach et al. (2018) and Schuller et al. (2018).

the MTR4 arch domain (Gerlach et al., 2018; Schuller et al., 2018). A small additional density was also present at the side of the MTR4$_A$ DExH-box domain that was interpreted by fitting the RBM7-binding module, as observed in the NEXT^L cryo-EM reconstruction. In addition, a density corresponding with the RNA substrate passing through both MTR4 and the exosome channel was visible, although the resolution was too low to discern individual ribonucleotides (Figure S6E). Altogether, the structural snapshot we obtained shows that a nuclear exosome can bind to a protomer of the NEXT complex.

Conclusions

The NEXT complex is organized around the ZCCHC8 subunit, which has both scaffolding and regulatory functions. In addition to enhancing the helicase activity of the complex (Puno and Lima, 2018), the ZCCHC8 CTD also gates the exit of the RNA 3′ end. Based on our structural and biochemical data, we propose a stepwise regulatory mechanism to explain this enigmatic function. In this model, an RNA substrate recognized by the NEXT complex would be encapsulated in the RNA channel of MTR4 (i.e., gatekeeping mode). Upon ATP binding, the ZCCHC8 CTD would promote the first rounds of ATP hydrolysis until the RNA 3′ end reaches the end of the helicase channel (i.e., activation mode). The exit of the RNA 3′ end from the helicase channel would be coupled with the displacement of the ZCCHC8 CTD by the incoming nuclear exosome. The active MTR4 subunit of NEXT would then be able to dock onto a nuclear exosome in a channeling configuration, threading the RNA 3′ end to the degradation machinery.

Each protomer in the NEXT homodimer is, in principle, capable of recruiting a nuclear exosome complex. Since the two MTR4 protomers are kept in opposite orientations upon binding to the homodimerization module of ZCCHC8, and since RNA binding in the DExH-box domain has a defined polarity, the interacting RNA molecules would in turn need to have an opposite orientation. Thus, the topology supports a model

CellPress
OPEN ACCESS

whereby each NEXT protomer can, in principle, independently recruit a substrate to the exosome, rationalizing the functional data that a monomeric NEXT mutant can be functional *in vivo*, at least on the substrates and in the conditions that we have tested (Figure S3B). There may be various benefits ingrained in this dimeric architecture. First, domain-swapping topologies are known to contribute to increased protein stability (MacKinnon and Wodak, 2015). Second, the dimerization module provides a platform for folding the major MTR4-binding region (AIM) in an appropriate conformation, possibly increasing the chances of ZCCHC8 to effectively compete against other MTR4-binding proteins. Third, RBM7 is known to accumulate upon loading of newly synthesized RNAs (Lubas et al., 2015) and thus these RNPs may bind with higher avidity by a dimeric ZCCHC8-MTR4 complex that intrinsically contains two RBM7-binding sites. The elaborate interaction networks of the NEXT-exosome assemblies go even beyond the 19- or 32-subunit complexes (containing either one or two EXO13) that we studied in this manuscript: the NEXT complex engages in higher-order assemblies by binding the ZC3H18 protein, which in turn interacts with the cap-binding complex and with histones (Winczura et al., 2018). Delineating the architecture of these higher-order assemblies will be required to ultimately understand the molecular mechanisms with which different classes of non-coding RNAs are recognized and targeted to exosome-mediated degradation.

Limitations of the study

A limitation of this study is that the individual portions of the cryo-EM maps do not reach atomic resolution and different reconstructions were combined to obtain a composite view of the complex. This limitation is likely a result of the flexibility of NEXT, but we deliberately chose nucleic acid substrates for use in this study that would render as close to a physiological situation as possible. Another potential limitation lies in the use of AlphaFold predictions in the cryo-EM interpretation of protein-protein complexes. Since AI-based approaches in this context have only been recently developed, their usage may still be optimized. Finally, a limitation in the cell-based experiments is that there may be other RNA substrates or conditions in which the importance of the NEXT complex dimerization manifests.

STAR★METHODS

Detailed methods are provided in the online version of this paper and include the following:

- KEY RESOURCES TABLE
- RESOURCE AVAILABILITY
  - Lead contact
  - Materials availability
  - Data and code availability
- EXPERIMENTAL MODEL AND SUBJECT DETAILS
- METHOD DETAILS
  - Protein expression and purification
  - Analytical gel filtration
  - Microscale thermophoresis
  - Mass spectrometry analysis of the BS3-crosslinked sample
  - RNase protection
  - Cryo-EM grid preparation
  - Cryo-EM data collection and processing
  - AlphaFold structure prediction
  - Cell culture and cell line generation
- CDNA CLONING AND EXOGENOUS EXPRESSION OF ZCCHC8
  - Western blotting analysis
  - Immunoprecipitation experiments
  - RNA isolation and RT-qPCR analysis
- QUANTIFICATION AND STATISTICAL ANALYSIS

SUPPLEMENTAL INFORMATION

Supplemental information can be found online at https://doi.org/10.1016/j.molcel.2022.04.011.

ACKNOWLEDGMENTS

We are grateful to Daniel Bollschweiler and Tillman Schäfer at the Max Planck Institute of Biochemistry (MPIB) cryo-EM facility for help with EM data collection and the MPIB core facilities for support. We thank Ingmar Schäfer, Christian Benda, and J. Rajan Prabu for maintenance of computational infrastructure for EM data processing; Courtney Long for help with the preparation of the manuscript and members of the lab for discussions; and Nadia Laurs Schmidt and Dorthe Caroline Riishøj from the Jensen lab for excellent technical assistance. This work was supported by funding from the Max Planck Society, the Novo Nordisk Foundation (ExoAdapt Grant 31199), the European Research Council (EXORICO ERC Advanced Grant 740329), and the German Research Foundation (DFG SFB 1035, GRK 1721, SFB/TRR 237) to E.C. and the Independent Research Fund Denmark – Medical Research Council (8020-00068B and 1030-00020B) and the Novo Nordisk Foundation (ExoAdapt grant 31199) to T.H.J.

AUTHOR CONTRIBUTIONS

E.C., T.H.J., and P.G. initiated the project; P.G. designed and performed the structural biology experiments; P.G. and J.B. built and refined atomic models; W.G. performed the cell-based experiments; P.G., A.S.-K., and F.B. performed the biochemical experiments; M.L. performed the biophysical experiments; and E.C., P.G., W.G., and T.H.J. wrote the manuscript.

DECLARATION OF INTERESTS

E.C. is a member of the Molecular Cell advisory board.

Received: November 10, 2021
Revised: March 7, 2022
Accepted: April 8, 2022
Published: June 9, 2022

SUPPORTING CITATIONS

The following references appear in the supplemental information: Rosenthal and Henderson (2003); South and Summers (1993); and Tan et al. (2017).

REFERENCES

Afonine, P.V., Klaholz, B.P., Moriarty, N.W., Poon, B.K., Sobolev, O.V., Terwilliger, T.C., Adams, P.D., and Urzhumtsev, A. (2018). New tools for the analysis and validation of cryo-EM maps and atomic models. Acta Crystallogr. D Struct. Biol. *74*, 814–840. https://doi.org/10.1107/S2059798318009324.

Bepler, T., Morin, A., Rapp, M., Brasch, J., Shapiro, L., Noble, A.J., and Berger, B. (2019). Positive-unlabeled convolutional neural networks for particle picking in cryo-electron micrographs. Nat. Methods *16*, 1153–1160. https://doi.org/10.1038/s41592-019-0575-8.

Biyani, N., Righetto, R.D., McLeod, R., Caujolle-Bert, D., Castano-Diez, D., Goldie, K.N., and Stahlberg, H. (2017). Focus: the interface between data collection and data processing in cryo-EM. J. Struct. Biol. *198*, 124–133. https://doi.org/10.1016/j.jsb.2017.03.007.

Bonneau, F., Basquin, J., Ebert, J., Lorentzen, E., and Conti, E. (2009). The yeast exosome functions as a macromolecular cage to channel RNA substrates for degradation. Cell *139*, 547–559. https://doi.org/10.1016/j.cell.2009.08.042.

Butler, J.S., and Mitchell, P. (2010). Rrp6, rrp47 and cofactors of the nuclear exosome. Adv. Exp. Med. Biol. *702*, 91–104. https://doi.org/10.1007/978-1-4419-7841-7_8.

Chlebowski, A., Lubas, M., Jensen, T.H., and Dziembowski, A. (2013). RNA decay machines: the exosome. Biochim. Biophys. Acta *1829*, 552–560. https://doi.org/10.1016/j.bbagrm.2013.01.006.

Ding, S., Wu, X., Li, G., Han, M., Zhuang, Y., and Xu, T. (2005). Efficient transposition of the piggyBac (PB) transposon in mammalian cells and mice. Cell *122*, 473–483. https://doi.org/10.1016/j.cell.2005.07.013.

Dziembowski, A., Lorentzen, E., Conti, E., and Séraphin, B. (2007). A single subunit, Dis3, is essentially responsible for yeast exosome core activity. Nat. Struct. Mol. Biol. *14*, 15–22. https://doi.org/10.1038/nsmb1184.

Emsley, P., Lohkamp, B., Scott, W.G., and Cowtan, K. (2010). Features and development of coot. Acta Crystallogr. D Biol. Crystallogr. *66*, 486–501. https://doi.org/10.1107/S0907444910007493.

Falk, S., Bonneau, F., Ebert, J., Kögel, A., and Conti, E. (2017a). Mpp6 incorporation in the nuclear exosome contributes to RNA channeling through the Mtr4 helicase. Cell Rep. *20*, 2279–2286. https://doi.org/10.1016/j.celrep.2017.08.033.

Falk, S., Finogenova, K., Melko, M., Benda, C., Lykke-Andersen, S., Jensen, T.H., and Conti, E. (2016). Structure of the RBM7-ZCCHC8 core of the NEXT complex reveals connections to splicing factors. Nat. Commun. *7*, 13573. https://doi.org/10.1038/ncomms13573.

Falk, S., Tants, J.-N., Basquin, J., Thoms, M., Hurt, E., Sattler, M., and Conti, E. (2017b). Structural insights into the interaction of the nuclear exosome helicase Mtr4 with the pre-ribosomal protein Nop53. RNA *23*, 1780–1787. https://doi.org/10.1261/rna.062901.117.

Falk, S., Weir, J.R., Hentschel, J., Reichelt, P., Bonneau, F., and Conti, E. (2014). The molecular architecture of the TRAMP complex reveals the organization and interplay of its two catalytic activities. Mol. Cell *55*, 856–867. https://doi.org/10.1016/j.molcel.2014.07.020.

Gable, D.L., Gaysinskaya, V., Atik, C.C., Talbot, C.C., Kang, B., Stanley, S.E., Pugh, E.W., Amat-Codina, N., Schenk, K.M., Arcasoy, M.O., et al. (2019). ZCCHC8, the nuclear exosome targeting component, is mutated in familial pulmonary fibrosis and is required for telomerase RNA maturation. Genes Dev. *33*, 1381–1396. https://doi.org/10.1101/gad.326785.119.

Garland, W., and Jensen, T.H. (2020). Nuclear sorting of RNA. Wiley Interdiscip. Rev. RNA *11*, e1572. https://doi.org/10.1002/wrna.1572.

Gerlach, P., Schuller, J.M., Bonneau, F., Basquin, J., Reichelt, P., Falk, S., and Conti, E. (2018). Distinct and evolutionary conserved structural features of the human nuclear exosome complex. eLife *7*, e38686. https://doi.org/10.7554/eLife.38686.

Giunta, M., Edvardson, S., Xu, Y., Schuelke, M., Gomez-Duran, A., Boczonadi, V., Elpeleg, O., Müller, J.S., and Horvath, R. (2016). Altered RNA metabolism due to a homozygous RBM7 mutation in a patient with spinal motor neuropathy. Hum. Mol. Genet. *25*, 2985–2996. https://doi.org/10.1093/hmg/ddw149.

Goddard, T.D., Huang, C.C., Meng, E.C., Pettersen, E.F., Couch, G.S., Morris, J.H., and Ferrin, T.E. (2018). UCSF ChimeraX: meeting modern challenges in visualization and analysis. Protein Sci. *27*, 14–25. https://doi.org/10.1002/pro.3235.

Hrossova, D., Sikorsky, T., Potesil, D., Bartosovic, M., Pasulka, J., Zdrahal, Z., Stefl, R., and Vanácová, S. (2015). RBM7 subunit of the NEXT complex binds U-rich sequences and targets 3′-end extended forms of snRNAs. Nucleic Acids Res. *43*, 4236–4248. https://doi.org/10.1093/nar/gkv240.

Imamura, K., Takaya, A., Ishida, Y.-I., Fukuoka, Y., Taya, T., Nakaki, R., Kakeda, M., Imamachi, N., Sato, A., Yamada, T., et al. (2018). Diminished nuclear RNA decay upon Salmonella infection upregulates antibacterial noncoding RNAs. EMBO J. *37*, e97723. https://doi.org/10.15252/embj.201797723.

Jackson, R.N., Klauer, A.A., Hintze, B.J., Robinson, H., van Hoof, A., and Johnson, S.J. (2010). The crystal structure of Mtr4 reveals a novel arch domain required for rRNA processing. EMBO J. *29*, 2205–2216. https://doi.org/10.1038/emboj.2010.107.

Jumper, J., Evans, R., Pritzel, A., Green, T., Figurnov, M., Ronneberger, O., Tunyasuvunakool, K., Bates, R., Žídek, A., Potapenko, A., et al. (2021). Highly accurate protein structure prediction with AlphaFold. Nature *596*, 583–589. https://doi.org/10.1038/s41586-021-03819-2.

Kilchert, C., Wittmann, S., and Vasiljeva, L. (2016). The regulation and functions of the nuclear RNA exosome complex. Nat. Rev. Mol. Cell Biol. *17*, 227–239. https://doi.org/10.1038/nrm.2015.15.

Kögel, A., Keidel, A., Bonneau, F., Schäfer, I.B., and Conti, E. (2022). The human SKI complex regulates channeling of ribosome-bound RNA to the exosome via an intrinsic gatekeeping mechanism. Mol. Cell *82*, 756–769.e8. https://doi.org/10.1016/j.molcel.2022.01.009.

LaCava, J., Houseley, J., Saveanu, C., Petfalski, E., Thompson, E., Jacquier, A., and Tollervey, D. (2005). RNA degradation by the exosome is promoted by a nuclear polyadenylation complex. Cell *121*, 713–724. https://doi.org/10.1016/j.cell.2005.04.029.

Langer, L.M., Bonneau, F., Gat, Y., and Conti, E. (2021). Cryo-EM reconstructions of inhibitor-bound SMG1 kinase reveal an autoinhibitory state dependent on SMG8. eLife *10*, e72353. https://doi.org/10.7554/eLife.72353.

Lingaraju, M., Johnsen, D., Schlundt, A., Langer, L.M., Basquin, J., Sattler, M., Heick Jensen, T., Falk, S., and Conti, E. (2019a). The MTR4 helicase recruits nuclear adaptors of the human RNA exosome using distinct arch-interacting motifs. Nat. Commun. *10*, 3393. https://doi.org/10.1038/s41467-019-11339-x.

Lingaraju, M., Schuller, J.M., Falk, S., Gerlach, P., Bonneau, F., Basquin, J., Benda, C., and Conti, E. (2019b). To process or to decay: a mechanistic view of the nuclear RNA exosome. Cold Spring Harb. Symp. Quant. Biol. *84*, 155–163. https://doi.org/10.1101/sqb.2019.84.040295.

Liu, Q., Greimann, J.C., and Lima, C.D. (2006). Reconstitution, activities, and structure of the eukaryotic RNA exosome. Cell *127*, 1223–1237. https://doi.org/10.1016/j.cell.2006.10.037.

Lubas, M., Andersen, P.R., Schein, A., Dziembowski, A., Kudla, G., and Jensen, T.H. (2015). The human nuclear exosome targeting complex is loaded onto newly synthesized RNA to direct early ribonucleolysis. Cell Rep. *10*, 178–192. https://doi.org/10.1016/j.celrep.2014.12.026.

Lubas, M., Christensen, M.S., Kristiansen, M.S., Domanski, M., Falkenby, L.G., Lykke-Andersen, S., Andersen, J.S., Dziembowski, A., and Jensen, T.H. (2011). Interaction profiling identifies the human nuclear exosome targeting complex. Mol. Cell *43*, 624–637. https://doi.org/10.1016/j.molcel.2011.06.028.

MacKinnon, S.S., and Wodak, S.J. (2015). Landscape of intertwined associations in multi-domain homo-oligomeric proteins. J. Mol. Biol. *427*, 350–370. https://doi.org/10.1016/j.jmb.2014.11.003.

Makino, D.L., Baumgärtner, M., and Conti, E. (2013). Crystal structure of an RNA-bound 11-subunit eukaryotic exosome complex. Nature *495*, 70–75. https://doi.org/10.1038/nature11870.

Makino, D.L., Schuch, B., Stegmann, E., Baumgärtner, M., Basquin, C., and Conti, E. (2015). RNA degradation paths in a 12-subunit nuclear exosome complex. Nature *524*, 54–58. https://doi.org/10.1038/nature14865.

Meola, N., Domanski, M., Karadoulama, E., Chen, Y., Gentil, C., Pultz, D., Vitting-Seerup, K., Lykke-Andersen, S., Andersen, J.S., Sandelin, A., and

Jensen, T.H. (2016). Identification of a nuclear exosome decay pathway for processed transcripts. Mol. Cell *64*, 520–533. https://doi.org/10.1016/j.molcel.2016.09.025.

Mirdita, M., Schütze, K., Moriwaki, Y., Heo, L., Ovchinnikov, S., and Steinegger, M. (2021). ColabFold—making protein folding accessible to all. bioRxiv. https://doi.org/10.1101/2021.08.15.456425.

Mitchell, P., Petfalski, E., Shevchenko, A., Mann, M., and Tollervey, D. (1997). The exosome: a conserved eukaryotic RNA processing complex containing multiple $3' \rightarrow 5'$ exoribonucleases. Cell *91*, 457–466. https://doi.org/10.1016/S0092-8674(00)80432-8.

Natsume, T., and Kanemaki, M.T. (2017). Conditional degrons for controlling protein expression at the protein level. Annu. Rev. Genet. *51*, 83–102. https://doi.org/10.1146/annurev-genet-120116-024656.

Nishimura, K., Fukagawa, T., Takisawa, H., Kakimoto, T., and Kanemaki, M. (2009). An auxin-based degron system for the rapid depletion of proteins in nonplant cells. Nat. Methods *6*, 917–922. https://doi.org/10.1038/nmeth.1401.

Ogami, K., Chen, Y., and Manley, J.L. (2018). RNA surveillance by the nuclear RNA exosome: mechanisms and significance. Noncoding RNA *4*, 8. https://doi.org/10.3390/ncrna4010008.

Olsen, K.J., and Johnson, S.J. (2021). Mtr4 RNA helicase structures and interactions. Biol. Chem. *402*, 605–616. https://doi.org/10.1515/hsz-2020-0329.

Pettersen, E.F., Goddard, T.D., Huang, C.C., Couch, G.S., Greenblatt, D.M., Meng, E.C., and Ferrin, T.E. (2004). UCSF Chimera—a visualization system for exploratory research and analysis. J. Comput. Chem. *25*, 1605–1612. https://doi.org/10.1002/jcc.20084.

Punjani, A., Rubinstein, J.L., Fleet, D.J., and Brubaker, M.A. (2017). cryoSPARC: algorithms for rapid unsupervised cryo-EM structure determination. Nat. Methods *14*, 290–296. https://doi.org/10.1038/nmeth.4169.

Puno, M.R., and Lima, C.D. (2018). Structural basis for MTR4-ZCCHC8 interactions that stimulate the MTR4 helicase in the nuclear exosome-targeting complex. Proc. Natl. Acad. Sci. USA *115*. E5506–E5515. https://doi.org/10.1073/pnas.1803530115.

Ran, F.A., Hsu, P.D., Wright, J., Agarwala, V., Scott, D.A., and Zhang, F. (2013). Genome engineering using the CRISPR-Cas9 system. Nat. Protoc. *8*, 2281–2308. https://doi.org/10.1038/nprot.2013.143.

Rohou, A., and Grigorieff, N. (2015). CTFFIND4: fast and accurate defocus estimation from electron micrographs. J. Struct. Biol. *192*, 216–221. https://doi.org/10.1016/j.jsb.2015.08.008.

Rosenthal, P.B., and Henderson, R. (2003). Optimal determination of particle orientation, absolute hand, and contrast loss in single-particle electron cryomicroscopy. J. Mol. Biol. *333*, 721–745. https://doi.org/10.1016/j.jmb.2003.07.013.

Schmid, M., and Jensen, T.H. (2018). Controlling nuclear RNA levels. Nat. Rev. Genet. *19*, 518–529. https://doi.org/10.1038/s41576-018-0013-2.

Schmid, M., and Jensen, T.H. (2019). The nuclear RNA exosome and its cofactors. Adv. Exp. Med. Biol. *1203*, 113–132. https://doi.org/10.1007/978-3-030-31434-7_4.

Schneider, C.A., Rasband, W.S., and Eliceiri, K.W. (2012). NIH Image to ImageJ: 25 years of image analysis. Nat. Methods *9*, 671–675. https://doi.org/10.1038/nmeth.2089.

Schorb, M., Haberbosch, I., Hagen, W.J.H., Schwab, Y., and Mastronarde, D.N. (2019). Software tools for automated transmission electron microscopy. Nat. Methods *16*, 471–477. https://doi.org/10.1038/s41592-019-0396-9.

Schuch, B., Feigenbutz, M., Makino, D.L., Falk, S., Basquin, C., Mitchell, P., and Conti, E. (2014). The exosome-binding factors Rrp6 and Rrp47 form a composite surface for recruiting the Mtr4 helicase. EMBO J. *33*, 2829–2846. https://doi.org/10.15252/embj.201488757.

Schuller, J.M., Falk, S., Fromm, L., Hurt, E., and Conti, E. (2018). Structure of the nuclear exosome captured on a maturing preribosome. Science *360*, 219–222. https://doi.org/10.1126/science.aar5428.

South, T.L., and Summers, M.F. (1993). Zinc- and sequence-dependent binding to nucleic acids by the N-terminal zinc finger of the HIV-1 nucleocapsid protein: NMR structure of the complex with the Psi-site analog, dACGCC. Protein Sci. *2*, 3–19. https://doi.org/10.1002/pro.5560020102.

Tan, Y.Z., Baldwin, P.R., Davis, J.H., Williamson, J.R., Potter, C.S., Carragher, B., and Lyumkis, D. (2017). Addressing preferred specimen orientation in single-particle cryo-EM through tilting. Nat. Methods *14*, 793–796. https://doi.org/10.1038/nmeth.4347.

Terwilliger, T.C., Ludtke, S.J., Read, R.J., Adams, P.D., and Afonine, P.V. (2020). Improvement of cryo-EM maps by density modification. Nat. Methods *17*, 923–927. https://doi.org/10.1038/s41592-020-0914-9.

Thoms, M., Thomson, E., Baßler, J., Gnädig, M., Griesel, S., and Hurt, E. (2015). The exosome is recruited to RNA substrates through specific adaptor proteins. Cell *162*, 1029–1038. https://doi.org/10.1016/j.cell.2015.07.060.

Tomecki, R., Kristiansen, M.S., Lykke-Andersen, S., Chlebowski, A., Larsen, K.M., Szczesny, R.J., Drazkowska, K., Pastula, A., Andersen, J.S., Stepien, P.P., et al. (2010). The human core exosome interacts with differentially localized processive RNaseS: hDIS3 and hDIS3L. EMBO J. *29*, 2342–2357. https://doi.org/10.1038/emboj.2010.121.

Tseng, C.-K., Wang, H.-F., Burns, A.M., Schroeder, M.R., Gaspari, M., and Baumann, P. (2015). Human telomerase RNA processing and quality control. Cell Rep. *13*, 2232–2243. https://doi.org/10.1016/j.celrep.2015.10.075.

Tunyasuvunakool, K., Adler, J., Wu, Z., Green, T., Zielinski, M., Žídek, A., Bridgland, A., Cowie, A., Meyer, C., Laydon, A., et al. (2021). Highly accurate protein structure prediction for the human proteome. Nature *596*, 590–596. https://doi.org/10.1038/s41586-021-03828-1.

Vanácová, S., Wolf, J., Martin, G., Blank, D., Dettwiler, S., Friedlein, A., Langen, H., Keith, G., and Keller, W. (2005). A new yeast poly(A) polymerase complex involved in RNA quality control. PLoS Biol. *3*, e189. https://doi.org/10.1371/journal.pbio.0030189.

Wang, J., Chen, J., Wu, G., Zhang, H., Du, X., Chen, S., Zhang, L., Wang, K., Fan, J., Gao, S., et al. (2019). NRDE2 negatively regulates exosome functions by inhibiting MTR4 recruitment and exosome interaction. Genes Dev. *33*, 536–549. https://doi.org/10.1101/gad.322602.118.

Wasmuth, E.V., and Lima, C.D. (2012). Exo- and endoribonucleolytic activities of yeast cytoplasmic and nuclear RNA exosomes are dependent on the noncatalytic core and central channel. Mol. Cell *48*, 133–144. https://doi.org/10.1016/j.molcel.2012.07.012.

Wasmuth, E.V., Zinder, J.C., Zattas, D., Das, M., and Lima, C.D. (2017). Structure and reconstitution of yeast Mpp6-nuclear exosome complexes reveals that Mpp6 stimulates RNA decay and recruits the Mtr4 helicase. eLife *6*, e29062. https://doi.org/10.7554/eLife.29062.

Weick, E.-M., Puno, M.R., Januszyk, K., Zinder, J.C., DiMattia, M.A., and Lima, C.D. (2018). Helicase-dependent RNA decay illuminated by a cryo-EM structure of a human nuclear RNA exosome-MTR4 complex. Cell *173*, 1663–1677.e21. https://doi.org/10.1016/j.cell.2018.05.041.

Weir, J.R., Bonneau, F., Hentschel, J., and Conti, E. (2010). Structural analysis reveals the characteristic features of Mtr4, a DExH helicase involved in nuclear RNA processing and surveillance. Proc. Natl. Acad. Sci. USA *107*, 12139–12144. https://doi.org/10.1073/pnas.1004953107.

Winczura, K., Schmid, M., Iasillo, C., Molloy, K.R., Harder, L.M., Andersen, J.S., LaCava, J., and Jensen, T.H. (2018). Characterizing ZC3H18, a multidomain protein at the interface of RNA production and destruction decisions. Cell Rep. *22*, 44–58. https://doi.org/10.1016/j.celrep.2017.12.037.

Wyers, F., Rougemaille, M., Badis, G., Rousselle, J.-C., Dufour, M.-E., Boulay, J., Régnault, B., Devaux, F., Namane, A., Séraphin, B., et al. (2005). Cryptic Pol II transcripts are degraded by a nuclear quality control pathway involving a new poly(A) polymerase. Cell *121*, 725–737. https://doi.org/10.1016/j.cell.2005.04.030.

Zhang, K. (2016). Gctf: real-time CTF determination and correction. J. Struct. Biol. *193*, 1–12. https://doi.org/10.1016/j.jsb.2015.11.003.

Zheng, S.Q., Palovcak, E., Armache, J.-P., Verba, K.A., Cheng, Y., and Agard, D.A. (2017). MotionCor2: anisotropic correction of beam-induced motion for improved cryo-electron microscopy. Nat. Methods *14*, 331–332. https://doi.org/10.1038/nmeth.4193.

Zinder, J.C., and Lima, C.D. (2017). Targeting RNA for processing or destruction by the eukaryotic RNA exosome and its cofactors. Genes Dev. *31*, 88–100. https://doi.org/10.1101/gad.294769.116.

Zinder, J.C., Wasmuth, E.V., and Lima, C.D. (2016). Nuclear RNA exosome at 3.1 Å reveals substrate specificities, RNA paths, and allosteric inhibition of Rrp44/Dis3. Mol. Cell *64*, 734–745. https://doi.org/10.1016/j.molcel.2016.09.038.

Zivanov, J., Nakane, T., Forsberg, B.O., Kimanius, D., Hagen, W.J., Lindahl, E., and Scheres, S.H. (2018). New tools for automated high-resolution cryo-EM structure determination in RELION-3. eLife *7*, e42166. https://doi.org/10.7554/eLife.42166.

STAR★METHODS

KEY RESOURCES TABLE

REAGENT or RESOURCE	SOURCE	IDENTIFIER
**Antibodies**		
Mouse monoclonal anti-FLAG M2	Sigma-Aldrich	Cat# F1804; RRID: AB_262044
Rabbit polyclonal anti-MTR4 (SKIV2L2)	Abcam	Cat# ab70551; RRID: AB_1270701
Rabbit monoclonal anti-MYC	Cell Signaling	Cat# 2278; RRID: AB_490778
Mouse monoclonal anti-MYC	Abcam	Cat# ab32; RRID:AB_303599
Rabbit polyclonal anti-RBM7	Sigma-Aldrich	Cat# HPA013993; RRID:AB_1856137
Rabbit polyclonal anti-ALPHA-TUBULIN	Rockland	Cat# 600-401-880, RRID:AB_2137000
**Bacterial and virus strains**		
BL21 Star (DE3) pRARE *E.coli* strain	EMBL Heidelberg Core Facility	N/A
**Chemicals, peptides, and recombinant proteins**		
H. sapiens MTR4	Lingaraju et al. (2019a); this paper	N/A
H. sapiens ZCCHC8	Falk et al. (2016), Puno and Lima (2018); this paper	N/A
H. sapiens RBM7	Falk et al. (2016); this paper	N/A
H. sapiens EXO10-MPP6-RRP6N-RRP47	Gerlach et al. (2018)	N/A
T4 DNA polymerase	NEB	NEB M0203L
T1 RNase	Thermo Fisher	EN0541
Benzonase	Merck	71206
3C Prescission protease	MPIB core facility	N/A
Sup2 SUMO protease	MPIB core facility	N/A
ATP, CTP, GTP, UTP	Jena Bioscience	NU-1010 – 1013
UTP [α-^{32}P]	Hartmann Analytic	FP-210
Indole-3-acetic acid sodium salt (IAA)	Sigma-Aldrich	I5148-10G
Trizol	Thermo Fisher	15596026
phenol:chloroform:isoamyl alcohol (25:24:1)	Thermo Fisher	15593031
n-octyl-ß-D-glucoside	Sigma-Aldrich	850511P
AMPPNP	Jena Bioscience	NU-407-10
BS_3 (bis(sulfosuccinimidyl)suberate)	Thermo Fisher	A39266
**Critical commercial assays**		
Phusion Flash High-Fidelity PCR Master Mix	Thermo Fisher	F548S
QIA prep Spin Miniprep Kit	Qiagen	27104
Wizard SV Gel and PCR Clean-up system	Promega	A9282
TURBO DNase kit	Thermo Fisher	AM2238
SuperScript III Reverse Transcriptase	Thermo Fisher	1808044
Platinum SYBR Green qPCR SuperMix	Thermo Fisher	11733046
Ribolock RNase Inhibitor	Thermo Fisher	EO0381
RiboCop rRNA Depletion kit	Lexogen GmbH	037.96
Protein G Dynabeads	Thermo Fisher	10009D
Lipofectamine 3000 Transfection Reagent	Thermo Fisher	L300001
Viafect Transfection Reagent	Promega	E4981
NEBuilder HiFi DNA Assembly cloning kit	NEB	E5520S
GeneJET PCR Purification Kit	Thermo Fisher	K0701

(*Continued on next page*)

Continued		
REAGENT or RESOURCE	SOURCE	IDENTIFIER
Deposited data		
NEXT^S overall reconstruction	This paper	EMDB: 14510<br>PDB: 7Z4Y
NEXT^S dimerization module	This paper	EMDB: 14511<br>PDB: 7Z4Z
NEXT^L single protomer	This paper	EMDB: 14514
NEXT^L focused on MTR4	This paper	EMDB: 14513<br>PDB: 7Z52
NEXT^S with nuclear exosome	This paper	EMDB: 14515
Raw and analyzed data	This paper	Mendeley doi: https://doi.org/10.17632/m5pg349jth.1
Experimental models: Cell lines		
HeLa:TIR1	Prof. Edouard Bertrand	N/A
HeLa:TIR1 *ZCCHC8-3F-mAID*	This study	N/A
HeLa:TIR1 *ZCCHC8-3F-mAID* MYC-ZCCHC8$^{1-707}$	This study	N/A
HeLa:TIR1 *ZCCHC8-3F-mAID* MYC-ZCCHC8$^{177-707}$	This study	N/A
Oligonucleotides		
sgRNA oligonucleotides	See Table S2	N/A
RT-qPCR oligonucleotides	See Table S3	N/A
RNA U_{20}	ELLA Biotech	N/A
RNA CUACCCCGAGAGGGGUAG-U_{60}	ELLA Biotech	N/A
Recombinant DNA		
pGCT[hZCCHC8-3F-mAID] HYG	This study	N/A
pGCT[hZCCHC8-3F-mAID] NEO	This study	N/A
pBAC[MYC-hZCCHC8_1-707] BLAST	This study	N/A
pBAC[MYC-hZCCHC8_177-707] BLAST	This study	N/A
pEC-Kan-His-GST-3C-hMTR4(FL)	This study	N/A
pEC-Kan-His-GST-3C-hZCCHC8(41-337)	Falk et al. (2016)	N/A
pEC-Kan-His-GST-3C-hZCCHC8(177-337)	This study	N/A
pEC-Kan-His-GST-3C-hZCCHC8(187-337)	This study	N/A
pEC-Kan-His-GST-3C-hZCCHC8(659-707)-TRX	This study	N/A
pEC-Strep-His-Ztag-3C-hRBM7(1-98)	Falk et al. (2016)	N/A
pEC-Kan-His-SUMO-hZCCHC8(Δ416-506)	Adapted from Puno and Lima (2018)	N/A
pEC-Amp-His-SUMO-hRBM7(1-98)	This study	N/A
Software and algorithms		
ImageJ (v1.51)	Schneider et al. (2012)	https://imagej.nih.gov/ij/
AriaMx (v1.71)	Agilent	https://www.agilent.com/
Graphpad Prism (9.0.0)	Graphpad	https://www.graphpad.com/scientific-software/prism/
AlphaFold	Jumper et al. (2021)	https://alphafold.ebi.ac.uk/
AlphaFold_advanced Google Colab notebook	Mirdita et al. (2021)	https://colab.research.google.com/github/sokrypton/ColabFold/blob/main/beta/AlphaFold2_advanced.ipynb
SerialEM	Schorb et al. (2019)	https://bio3d.colorado.edu/SerialEM/
Focus	Biyani et al. (2017)	https://www.focus-em.org
MotionCor2	Zheng et al. (2017)	https://msg.ucsf.edu/em/ software/motioncor2.html

(*Continued on next page*)

Continued

REAGENT or RESOURCE	SOURCE	IDENTIFIER
GCTF	Zhang (2016)	https://www2.mrc-lmb.cam.ac.uk/research/locally-developed-software/zhang-software/#gctf
CtfFind4.1	Rohou and Grigorieff (2015)	https://grigoriefflab.umassmed.edu/ctffind4
Gautomatch	N/A	https://www2.mrc-lmb.cam.ac.uk/research/locally-developed-software/zhang-software/#gauto
RELION 3.0 and 3.1	Zivanov et al. (2018)	https://github.com/3dem/relion
cryoSPARC v2	Punjani et al. (2017)	https://cryosparc.com/
Topaz	Bepler et al. (2019)	https://topaz.csail.mit.edu
PHENIX 1.19.2	Afonine et al. (2018)	https://www.phenix-online.org/
COOT 0.8.9.2 EL	Emsley et al. (2010)	https://www2.mrc-lmb.cam.ac.uk/personal/pemsley/coot/
PyMOL 2.3.5	PyMOL Molecular Graphics System, Schrodinger LLC	https://pymol.org/2/
Chimera	Pettersen et al. (2004)	https://www.cgl.ucsf.edu/chimera/
ChimeraX	Goddard et al. (2018)	https://www.cgl.ucsf.edu/chimerax/
Other		
NuPAGE 4-12% Bis-Tris protein gels	Thermo Fisher	NP0321BOX
HIS-select nickel affinity resin	Sigma-Aldrich	P6611-100ML
HiTrap Heparin HP column 1 mL and 5 mL	Cytiva	17040601, 17040703
Superdex 200 Increase 10/300 column	Cytiva	28990944
Superdex 200 Increase 3.2/300 column	Cytiva	28990946
Superose 6 Increase 3.2/300 column	Cytiva	29091598
Amicon Ultra MWCO50	Merck	UFC9050
Quantifoil R2/1, Cu 200 mesh	Quantifoil	N1-C15nCu20-01

RESOURCE AVAILABILITY

Lead contact

Further information and requests for resources and reagents should be directed to and will be fulfilled by the Lead Contact, Elena Conti conti@biochem.mpg.de.

Materials availability

This study did not generate new unique reagents.

Data and code availability

- Cryo-EM density maps and atomic models have been deposited in the Electron Microscopy Data Bank and the Protein Data Bank, respectively, under the accession numbers: EMDB: 14510 and PDB: 7Z4Y (human NEXT dimer – overall reconstruction of the core complex), EMDB: 14511 and PDB: 7Z4Z (human NEXT dimer – focused reconstruction of the dimerization module), EMDB: 14513 and PDB: 7Z52 (human NEXT dimer – focused reconstruction of the single MTR4), EMDB: 14514 (human NEXT dimer – sinlge protomer at low resolution), EMDB: 14515 (human NEXT dimer in complex with the nuclear RNA exosome). Data are available at time of publication. Unprocessed and uncompressed imaging data is available at Mendeley Data: https://doi.org/10.17632/m5pg349jth.1.
- This paper does not report original code.
- Any additional information required to reanalyze the data reported in this work/paper is available from the lead contact upon request.

EXPERIMENTAL MODEL AND SUBJECT DETAILS

Bacterial cells in this study were used for protein production for *in vitro* experiments and structural analysis. Human cell lines were used for immunoprecipitation of protein complexes and for *in vivo* assays. None of the cells in this study were used as experimental

models in the typical sense. Handling of both bacterial and eukaryotic cells is described in detail in the STAR Methods section. All cell types used are listed in the key resources table.

METHOD DETAILS

Protein expression and purification

All proteins were recombinantly expressed in BL21 Star (DE3) pRARE *E. coli* cells grown at 37°C in TB media up to OD_{600} 1.0–1.5, and induced with 0.5 mM IPTG for overnight expression at 18°C. The full-length MTR4 (UniProtKB: P42285) was expressed with an N-terminal 6xHis-GST-3C tag. The RBM7 (UniProtKB: Q9Y580) construct encompassing the RRM domain (1-98) was expressed with either an N-terminal 6xHis-Z-3C tag or an N-terminal 6xHis-SUMO tag. The ZCCHC8 (UniProtKB: Q6NZY4) constructs were expressed with either an N-terminal 6xHis-GST-3C tag or an N-terminal 6xHis-SUMO tag. While MTR4 was expressed and purified separately, all combinations of the RBM7-ZCCHC8 dimer units were co-expressed and co-purified. Bacteria were lysed by sonication in 20 mM Hepes-NaOH pH=7.5, 500 mM NaCl, 20 mM imidazole, 5 mM β-mercaptoethanol, 0.5 mM AEBSF, and 15 U/ml benzonase (Merck). Cleared lysate was loaded on the HIS-select nickel affinity resin (Sigma-Aldrich), washed with the chaperone wash buffer (lysis buffer supplemented with 1M NaCl, 10 mM MgSO4, 50 mM KCl, and 2 mM ATP), and eluted with 300 mM imidazole. During overnight dialysis at 4°C in the buffer containing 20 mM Hepes-NaOH pH=7.5, 150 mM NaCl, 20 mM imidazole, and 5 mM β-mercaptoethanol, affinity tags were cleaved by the 3C protease or Sup2 SUMO protease. Following the second nickel affinity step which removed cleaved tags and proteases, protein samples were subjected to the HiTrap Heparin HP column (GE Healthcare) and eluted with NaCl gradient. Subsequently, pre-purified MTR4 was mixed with various RBM7-ZCCHC8 dimers, concentrated, and resolved by size exclusion chromatography on a Superdex 200 Increase 10/300 column (GE Healthcare) pre-equilibrated with 20 mM Hepes-NaOH (pH 7.5), 150 mM NaCl, 2 mM DTT.

The human nuclear exosome complex EXO10-MPP6-RRP6^{N}-RRP47, containing fusion constructs MPP6(FL)-RRP40(FL) and RRP4(FL)-RRP6(1-160) co-expressed with RRP47(FL), was prepared as described previously (Gerlach et al., 2018).

Analytical gel filtration

Variants of the NEXT complexes composed of full the length MTR4, RBM7(RRM) domain, and various N-terminally truncated ZCCHC8 constructs terminating at residue 337, were analyzed over a Superdex 200 Increase 3.2/300 analytical gel filtration column (GE Healthcare), pre-equilibrated with 20 mM Hepes-NaOH (pH 7.5), 150 mM NaCl, 2 mM DTT. In each case 30 μL containing 300 pmol of the pre-assembled NEXT complexes were injected resulting in 2-3 μM concentration in the peak fractions of resolved complexes. Absorption readings at 280 nm were normalized and plotted overlayed to compare retention times using the single MTR4 run as reference.

Microscale thermophoresis

The microscale thermophoresis measurements were performed on a NanoTemper Monolith NT.115 machine. Before the measurements, all samples were dialyzed against a buffer containing in 50 mM HEPES-NaOH (pH 7.5), 150 mM NaCl, 2 mM DTT, 10% (v/v) glycerol, and 0.1% Pluronic F-127. The eYFP-(GS)3-MTR4(75-1042) at fixed concentration of 50 nM was incubated for 15 min at room temperature with increasing concentrations of either monomeric ZCCHC8(177-337)/RBM7(RRM), or dimeric ZCCHC8(41-337)/RBM7(RRM). Concentration of the dimeric ZCCHC8/RBM7 unit was calculated using its monomeric molecular weight, in order to have comparable concentrations of AIMs per volume per concentration step in both experiments. Thermophoresis was measured with MST power of 20%, LED power of 20%, and standard parameters on a NanoTemper Monolith NT.115 machine. The binding isotherm and the dissociation constant (Kd) were calculated using MO software (NanoTemper technologies). Titrations were performed in triplicates to estimate the standard deviations.

Mass spectrometry analysis of the BS3-crosslinked sample

20 uL of the mutant NEXT^{S} sample (i.e. containing ZCCHC8^{S}, residues 177-337) at 1ug/uL was supplemented with 1:1 molar excess of the RNA-U_{20} and 2 mM AMPPNP. Sample was crosslinked with 0.05 mM BS3 for 30 min at RT and quenched with 50 mM TRIS pH 8.0. Subsequently, samples were digested with trypsin and the resulting crosslinked peptides analyzed at the MPIB mass spectrometry facility, following the protocol described in (Langer et al., 2021).

RNase protection

Body-labeled RNAs were generated by in vitro transcription with T7 RNA polymerase in presence of [α-^{32}P] UTP (Perkin-Elmer) and RNase T1 (ThermoFisher), to remove leading guanosines, followed by denaturing gel purification. Templates were obtained by annealing of two DNA oligonucleotides containing the T7 promoter sequence. The final sequence for the 57-mer was (CU)28C. Proteins (5 pmol each) were mixed with 2.5 pmol ^{32}P body-labeled RNA to a final 10 μl reaction volume in 50 mM HEPES-NaOH (pH 7.5), 50 mM NaCl, 5 mM magnesium diacetate, 10% (w/v) glycerol, 0.1% (w/v) NP40, and 1 mM DTT. After incubation for 45 min at 4°C, reactions mixtures were treated with 375 U benzonase (Merck), for 20 min at 25°C. Protected RNA fragments were then extracted twice with phenol:chloroform:isoamyl alcohol (25:24:1, v/v, Invitrogen), precipitated with ethanol, separated on 12% (w/v) and 7 M urea denaturing PAGE, and visualized by phosphorimaging (Cytiva).

Cryo-EM grid preparation

To prepare the optimized grids used for collection of the NEXT^S dataset, the protein complex at 2.5 mg/ml = 15 μM in a buffer containing 20 mM HEPES-NaOH (pH 7.5), 150 mM NaCl, 2 mM DTT, 2 mM $MgCl_2$, and 2 mM AMPPNP, was mixed with 1.2 molar excess of the RNA-U_{20}. Following 30 min incubation at room temperature, the NEXT^S-RNA sample was crosslinked with 1 mM bis-sulfosuccinimidyl suberate (BS3) for 30 min, quenched with 10 mM TRIS-HCl (pH 8.0), and supplemented with 0.02% octyl-β-glucoside (βOG) and 5% glycerol.

To prepare the optimized grids used for collection of the NEXT^L dataset, the protein complex was first resolved on the Superdex 200 Increase 3.2/300 analytical gel filtration column (GE Healthcare) in a buffer containing 20 mM HEPES-NaOH (pH 7.5), 50 mM NaCl, and 2 mM DTT. The shoulder fraction right after the peak, at 0.4 mg/ml = 2 μM, was supplemented with 2 mM $MgCl_2$, and 2 mM AMPPNP, and mixed with equimolar amount of the RNA 5′-hairpin-U_{60} (CUACCCCGAGAGGGGUAG-U_{60}). Following 30 min incubation at room temperature, the NEXT^L-RNA sample was crosslinked with 0.03% glutaraldehyde (GA) for 10 min, quenched with 10 mM TRIS-HCl (pH 8.0), and supplemented with 0.02% βOG.

To prepare the optimized grids used for collection of the NEXT^S-EXO dataset, equimolar amounts of the NEXT^S sample and the EXO10-MPP6-RRP6^N-RRP47 sample were mixed in a buffer containing 20 mM HEPES-NaOH (pH 7.5), 150 mM NaCl, 2 mM DTT, 2 mM $MgCl_2$, and 1 mM ADP. Protein sample was subsequently supplemented with 1.2 molar excess of the RNA 5′-hairpin-U_{60} (CUACCCCGAGAGGGGUAG-U_{60}). Following 30 min incubation at room temperature, the NEXT^S-EXO-RNA sample was crosslinked with 1.5 mM BS3 for 1 hour, quenched with 20 mM $(NH_4)_2CO_3$, and resolved on the Superose 6 Increase 3.2/300 analytical gel filtration column (GE Healthcare). The peak fraction at 0.4 mg/ml was supplemented with 0.04% βOG and used for grid preparation.

In all three cases, 4 μl of the sample was applied onto Quantifoil R2/1, Cu 200 mesh grid, glow-discharged for 20-30 sec with the GloQube glow-discharger. Excess of the sample was blotted away at 95% humidity and 4°C, using the FEI Vitrobot Mark IV set to blot force 4, blot time 3.5 sec, and plunged-frozen in the ethane-propane cooled with liquid nitrogen.

Cryo-EM data collection and processing

High resolution cryo-EM movies for the NEXT^S dataset and the NEXT^L dataset were collected on a Titan Krios microscope (TFS) operating at 300 kV, equipped with a post-column Gatan imaging energy filter (GIF) with the energy slit width set to 20 eV, and a Gatan K3 camera operated in counting mode. SerialEM was used for data acquisition (Schorb et al., 2019). To increase throughput, the software's coma-free "Multiple Record" function was employed. Using image shift, two images per hole in an array of nine holes (3x3 pattern) were recorded within each preset stage position. Coma-free alignment for these beam settings was previously calibrated on a standard cross grating. FOCUS software was used to pre-select recorded movies during acquisition, allowing to pass only those with CTF max resolution < 5 Å (Biyani et al., 2017). High resolution cryo-EM movies for the NEXT^S-EXO dataset were collected on a Titan Krios microscope (TFS) operating at 300 kV, equipped with a post-column Gatan imaging energy filter (GIF) with the energy slit width set to 20 eV, and a Gatan K2 camera operated in counting mode. In this case the SerialEM multiple record routine and the FOCUS pre-selection were not applied. Both cryoSPARC v2 (Punjani et al., 2017) and Relion 3.1 (Zivanov et al., 2018) were used for data processing. Despite dimeric nature of the NEXT complex, imposing the C2 symmetry resulted in much worse 3D reconstructions. Therefore, wherever it was necessary, the C1 symmetry was imposed.

For the NEXT^S dataset, 11,393 movies were recorded at 81,000 × magnification, corresponding to a calibrated pixel size of 1.094 Å. Total exposure of 64.85 $e^-/Å^2$ in 4.65 sec was fractionated over 31 frames, with applied defocus ranging from -0.5 to -2.3 μm in 0.3 μm steps. All frames were included in movie drift correction and dose weighted with "patch motion correction" followed by "patch CTF estimation", both done in cryoSPARC. Initially, particles were automatically picked using 2D templates from a screening dataset. Best template picking output was achieved by setting particle diameter to 250 Å, and distance between the particles 0.7 × 250 Å = 175 Å. Picked particles were extracted from aligned micrographs using a box size of 384 pixels. Selected 2D classes after three rounds of classification were used for the Topaz wrapper (Bepler et al., 2019) to improve the picking accuracy. Topaz was trained on a subset of 7760 selected particles from a random set of 100 micrographs, expecting 400 particles per micrograph. 3,280,391 topaz-picked particles were extracted using same box size as above. Subsequently, three rounds of 2D classifications were used to exclude bad quality and off-centered particles, resulting in 1,215,456 particles suitable for further image processing. Particles were then 3D refined in a homogenous refinement step, using as reference model the 30 Å low pass filtered 3D volume from a screening dataset. Refined particles were re-extracted with a box size of 200 pixels, tightened around the NEXT dimerization interface, and submitted for another round of homogenous refinement. Particles from both refinements were then exported to Relion for further processing. Particle stacks mrc files were renamed into mrcs and the cs files were converted into the star files using the pyem module and the csparc2star.py command.

Particles within the 384-pixel box were used to obtain an overall NEXT dimer reconstruction. Following 3D classification into 50 classes, without image alignment, 1,132,953 particles were split into three subsets and subjected to 3D classification into six classes, applying regularization parameter T=6 and initial angular sampling of 1.8°. 401,736 selected and combined particles were again 3D classified, using the same parameters and narrowed down the selection to 189,339 particles. Following the 3D auto-refinement and post-processing (b-factor -98.5), the final overall NEXT dimer reconstruction (EMD-14510) reached an overall resolution of 4.5 Å with local resolution ranging from 4.0 Å to 9.1 Å as estimated by Relion.

Particles within the 200-pixel box were used to obtain high resolution map of the NEXT dimerization interface. Following 3D classification into 50 classes, without image alignment, 932,289 particles were subjected to 3D classification into twelve classes,

applying regularization parameter T=10 and initial angular sampling of 1.8°. 134,072 selected particles were refined and 3D classified into five classes, applying regularization parameter T=15 and initial angular sampling of 1.8°. Final set of 55,022 selected particles was refined and subjected to signal subtraction in order to maintain only the signal corresponding with minimal dimerization unit composed of MTR4 Arch/KOW domains and ZCCHC8 N-termini. The last round of 3D auto-refinement and post-processing (b-factor -137.6) led to the final focused NEXT dimerization interface reconstruction (EMD-14511) at an overall resolution of 4.0 Å with local resolution ranging from 3.8 Å to 5.6 Å as estimated by Relion. Alternatively, to the Relion post-processing routine, the refined map was improved by Phenix resolve_cryo_em density modification (Terwilliger et al., 2020) to facilitate model building.

For the $NEXT^L$ dataset, 23,691 movies were recorded at 105,000 × magnification, corresponding to a calibrated pixel size of 0.8512 Å. Total exposure of 68.2 $e^-/Å^2$ in 6 sec was fractionated over 40 frames, with applied defocus ranging from -0.5 to -2.3 μm in 0.3 μm steps. All frames were included in movie drift correction and dose weighting using the Relion implementation of MotionCor2 (Zheng et al., 2017), followed by CTF estimation done with the Relion wrapper for CtfFind4.1 (Rohou and Grigorieff, 2015). Template-based particle picking, using 2D classes from a screening dataset, was performed with Gautomatch. Picked particles were extracted from aligned micrographs using a box size of 512 pixels down-sampled to 64 pixels (6.8096 Å/pix). Selected 2D classes after three rounds of classification were subjected to a single round of 3D classification into six classes, using as reference model the 30 Å low pass filtered 3D volume from a screening dataset, applying regularization parameter T=4 and initial angular sampling of 7.5°. 2D projections of the best 3D class were used to redo the template-based picking and improve the Euler angles coverage. 5,697,104 picked particles were extracted as above, down-sampling the 512-pixel box size to 64 pixels, and were 3D classified in two subsets, using parameters described above. 1,566,261 combined and refined particles were subjected to similarly set up 3D classification but applying a mask covering one half of the NEXT complex dimer. Four out of six classes were selected based on the presence of a feature between the MTR4 KOW domain and MTR4 Rec-A core which, as we hypothesized, could correspond with the RBM7-RRM domain. 1,016,777 combined particles were re-centered while re-extracting into twice smaller 256-pixel box size. Processing scheme diverged at this point to achieve to different goals: a) to determine the highest possible resolution reconstruction of the MTR4 core to reveal the RNA substrate and ZCCHC8-CTD bound to it (Figure S1H, right side of the panel); and b) to narrow down selection of particles containing the RBM7-RRM feature, and to determine the reconstruction revealing how the ZCCHC8 scaffolds the NEXT complex (Figure S1H, left side of the panel). In both cases particles were subjected into three rounds refinement followed by 3D classification into six classes. On the way the original 0.8512 Å/pix sampling was restored, keeping the 256-pixel box size. High resolution MTR4 core structure reconstruction was determined with 224,200 particles and reached an overall resolution of 3.4 Å. During determination of the structure containing density for RBM7-RRM, a significant difference was the application of local angular search during the last round of 3D classification. Single 3D class with the best pronounced feature of interest was additionally subjected to two rounds of refinement followed by 3D classification into three classes, keeping the local angular search, and applying regularization parameter T=6 and initial angular sampling of 3.7° and 1.8°. The final reconstruction used to analyze position of the RBM7-RRM and scaffolding of the ZCCHC8 was determined with 10,594 particles and reached an overall resolution of 6.8 Å.

For the $NEXT^S$-EXO dataset, 8,245 movies were recorded at 105,000 × magnification, corresponding to 1.35 Å/pix at the specimen level. Total exposure of 46.8 $e^-/Å^2$ in 6 sec was fractionated over 40 frames, with applied defocus ranging from -0.5 to -3.5 μm in 0.5 μm steps. The dose-fractionated movies were gain normalized, aligned and dose-weighted using MotionCor2 (Zheng et al., 2017), followed by CTF estimation done with the Relion wrapper for GCTF (Zhang, 2016). 2,595,547 particles were reference-free picked using Gautomatch and extracted using box size of 260 pixels, from 8,045 aligned micrographs that passed the 4.2 CTF max resolution cutoff. After a single round of 2D classification performed in 10 subsets, 820,312 selected particles were 3D classified into six classes, using as reference model the 30 Å low pass filtered human EXO14 map (EMD-0127 from Gerlach et al., 2018). There was a single good quality class corresponding with the exosome complex alone, and another one corresponding with the NEXT-EXO assembly, although revealing density for a single MTR4 only and with poor density for the remaining parts of the NEXT complex. 94,214 particles from this latter class were refined, re-extracted using box size of 400 pixels re-centering on the NEXT dimerization interface, and refined again. The rational was to position the NEXT complex in the center of the box while leaving enough space to accommodate two exosomes, potentially bound on both sides. Applying a narrow circular mask of 225 Å during consecutive three rounds of 2D classification led to selection of 21,806 particles containing the previously mis-aligned NEXT complex dimer. The fourth round of 2D classification was performed without alignment, applying a broad circular mask of 535 Å to reveal the exosomes attached to the NEXT complex. There were 14 classes of the EXO-NEXT-EXO (5,633 particles) and 36 classes of the NEXT-EXO (11,273). All 21,806 particles were then 3D classified into three classes using as reference the 30 Å low pass filtered map of the composite EXO-NEXT-EXO model. One of the classes, with 2,356 particles, revealed a complete NEXT complex dimer attached to a single exosome. This class was 3D refined to reach the nominal resolution of 9.5 Å. Further 3D focused refinement, with the mask covering MTR4-EXO9, improved the nominal resolution to 8.0 Å allowing for better visualization of the RNA substrate in the channel.

AlphaFold structure prediction

Open access AlphaFold software (Jumper et al., 2021; Tunyasuvunakool et al., 2021) was used to model several missing regions of the NEXT complex. Structure predictions were done for the 1-260 region of ZCCHC8 protein (containing the unstructured N-terminal region (1-43), the coiled-coil α1 helices (44-77), the dimerization module, the AIM and AIM+ regions up to residue 220, and the zinc-finger followed by the α3 helix up to residue 260), two short ZCCHC8 elements tethering the RBM7-binding module to the MTR4 RecA2 domain, and the MTR4 N-terminal region (75-97). AlphaFold was executed online via the Google Colab notebook

AlphaFold2_advanced (Mirdita et al., 2021). The ZCCHC8 region 1-260 was submitted as homodimer (homooligomer = 2). MMseqs2 approach was used for the multiple sequence alignment (msa_method = mmseqs2), starting with an initial search against the UniRef30 (a clustered version of the UniRef100), followed by an iterative search against the BFD/MGnify or ColabFold databases. AlphaFold2_advanced notebook does not use templates.

Cell culture and cell line generation

HeLa cell lines expressing *Oryza sativa* TIR1 (OsTIR1) were kindly provided by Prof. Edouard Bertrand. Cells were cultured in Dulbecco's modified Eagle's medium (DMEM, Gibco) supplemented with 10% fetal bovine serum (FBS) and 1% penicillin/streptomycin at 37°C, 5% CO_2.

CRISPR/Cas9 mediated genomic knock-in of C-terminal 3xFLAG-mini-AID (3F-mAID) tags were carried out using homology dependent repair (HDR) donor vectors (pGCT). Plasmids were generated containing ZCCHC8 specific 5′ and 3′ homology arms (HA) ~500 bp amplified from wildtype HeLa genomic DNA and cloned into pGCT donor vectors to comprise of 5' homology arm-[3xFLAG]-mAID-P2A-[HYG/NEO]-3′ homology arm. Single guide RNA (sgRNA) targeting the 3′ UTR of the ZCCHC8 locus was cloned into the pSpCas9(BB) vector (pX330, Addgene plasmid ID: #42230) as previously described (Ran et al., 2013). sgRNA sequences are included in Table S2. For knock-ins, $5x10^5$ OsTIR1 cells were co-transfected in 6-well plates using Lipofectamine 3000 (Thermo) with two pGCT donor vectors harbouring distinct selection markers along a sgRNA/Cas9 vector in a 1:1:1 ratio. 48 hours following transfection, cells were put under selection with 100 μg/ml Hygromycin (Invitrogen) and 700 μg/ml G418 (Gibco). Cells that survived selection were seeded at single cell density into 96 well plates and single colonies were expanded and screened by genotyping PCR and validated by western blotting.

For AID-mediated protein depletion, 750 μM indole-3-acetic acid sodium salt (IAA, Sigma-Aldrich) was added to the culture medium for the indicated time periods.

cDNA CLONING AND EXOGENOUS EXPRESSION OF ZCCHC8

ZCCHC8 cDNA constructs were cloned, using a full-length cDNA plasmid as a template, into a piggyBAC (pB) vector containing an N-terminal MYC tag and blastidicin (BSD) selection marker using NEBbuilder HiFi DNA assembly (NEB). *ZCCHC8-3F-mAID* cells were transfected with pB-ZCCHC8-BSD vectors along with a piggyBAC transposase expressing vector (pBase) in a 1:1 ratio using Viafect (Promega). Cell pools were selected with BSD for ~ 5-7 days or until negative control cells no longer survived. Expression of constructs were validated by western blotting analysis using MYC antibodies.

Western blotting analysis

Whole cell protein lysates for cell line validations were prepared using RSB100 (10 mM Tris-HCl pH7.5, 100 mM NaCl, 2.5 mM $MgCl_2$, 0.5% v/v NP-40, 0.5% v/v Triton X-100) freshly supplemented with protease inhibitors (Roche). Samples were denatured by the addition of NuPAGE Loading Buffer (Invitogen) and NuPAGE Sample Reducing Agent (Invitrogen) before boiling at 95°C for 10 minutes. SDS PAGE was carried out on either NuPAGE 4-12% Bis-Tris or 3-8% Tris-Acetate gels (Invitrogen). Western blotting analysis was carried out according to standard protocols with the antibodies listed in key resource table and HRP conjugated secondary antibodies (Agilent). Bands were visualized by Super Signal West Fempto chemiluminescent ECL (Thermo) and captured using an ImageQuant 800 imaging system (GE Healthcare). Images were processed using ImageJ (v1.51) (Schneider et al., 2012).

Immunoprecipitation experiments

Immunoprecipitations were carried out using whole cell extract from ~ $2x10^7$ cells per pulldown. Lysates were prepared in HT150 extraction buffer (20 mM HEPES pH 7.4, 150 mM NaCl, 0.5% v/v Triton X-100) freshly supplemented with protease inhibitors. Lysates were sheared by sonication (3x 5 s, amplitude 2) and cleared by centrifugation at 18,000 rcf for 20 minutes. Clarified lysates were incubated with 1 μg MYC antibody overnight at 4°C with Protein G Dynabeads (Thermo). Beads were washed 3 times with HT150 extraction buffer, transferring beads to a fresh tube on the final wash. Proteins were eluted by boiling in 1X NuPAGE loading buffer (Invitrogen) for 5 minutes. Supernatants were mixed with 10X Reducing Agent (Invitrogen) and denatured for a further 5 minutes at 95°C before proceeding with western blotting analysis.

RNA isolation and RT-qPCR analysis

Total RNA was isolated by TRIzol extraction (Thermo) using the standard procedure. Isolated RNA was treated with TURBO DNase (Invitrogen) following the manufacturer's instructions followed by cDNA preparation from 1 μg RNA using Superscript III reverse transcriptase (Invitrogen) and a mix of 80 pmol random primers and 20 pmol dT20 primers. qPCR was performed using Platinum SYBR Green using an AriaMx Real-Time PCR machine (Agilent). Primers for RTqPCR are listed in Table S3.

QUANTIFICATION AND STATISTICAL ANALYSIS

All statistical analyses are indicated in the legends to the relevant figure and in the corresponding STAR Methods section.

Molecular Cell, Volume *82*

Supplemental information

Structure and regulation of the nuclear exosome targeting complex guides RNA substrates to the exosome

Piotr Gerlach, William Garland, Mahesh Lingaraju, Anna Salerno-Kochan, Fabien Bonneau, Jérôme Basquin, Torben Heick Jensen, and Elena Conti

Figure S1. NEXT^S and NEXT^L cryo-EM sample preparation and data processing (related to Figure 1)

A) Size exclusion chromatography and SDS-PAGE of the NEXT^S sample used for cryo-EM structure determination

B) Representative cryo-EM micrograph collected on a FEI Titan Krios operated at 300 kV, equipped with a K3 Summit camera.

C) Representative 2D class averages of the selected NEXT^S particles. Box size (B) = 384 pix × 1.09 Å = 420 Å

D) NEXT^S Cryo-EM data processing scheme. The classification strategy on the bottom left resulted in a 3D reconstruction of the overall NEXT^S homodimer at 4.5 Å while the strategy on the bottom right resulted in a 4.0 Å focused reconstruction of the ZCCHC8 dimerization module interacting with two MTR4 KOW domains.

E) Size exclusion chromatography and SDS-PAGE of the NEXT^L sample used for cryo-EM structure determination

F) Representative cryo-EM micrograph collected on a FEI Titan Krios operated at 300 kV, equipped with a K3 Summit camera.

G) Representative 2D class averages of the selected NEXT^L complete homodimer particles (processing scheme not shown). Box size (B) = 300 pix × 0.8512 Å = 255 Å

H) Cryo-EM data processing scheme. All pre-processing and processing steps were done in Relion. The selection strategy depicted on the bottom left of the scheme led to the 6.8 Å reconstruction of a single NEXT protomer, revealing features like the ZCCHC8 zinc-finger domain and RBM7-binding module. The strategy depicted on the bottom right of the scheme led to the 3.4 Å reconstruction of a single MTR4.

I) Origin of structural elements used to build composite model of the NEXT homodimer. MTR4 DExH-box together with the ZCCHC8 CTD (Puno and Lima, 2018), were rigid-body fitted and refined within the 3.4 Å density of the focused MTR4 density. The MTR4 KOW and the closer pair of the stalk α-helices were rigid-body fitted and refined within the 4.0 Å density of the focused NEXT dimerization module. The ZCCHC8 dimerization module, both AIM and AIM+, and the C-terminal portion of the coiled-coil α1 helices were built de novo in the 4.0 Å focused density. The MTR4 N-terminus (residues 75-97), the entire coiled-coil α1 helices (residues 45-77), the ZCCHC8 *zinc*-finger followed by α3 helix (residues 220-260), and two

short ZCCHC8 fragments tethering the RBM7-binding module to the MTR4 RecA2 domain, were modelled with the AlphaFold and rigid body fitted in either the overall 4.5 Å NEXT^S density, or the 6.8 Å resolution density of the single NEXT^L protomer. The RBM7-binding module (Falk et al., 2016) was rigid-body fitted in the 6.8 Å resolution density of the single NEXT^L protomer. The gray, graduated triangle below depicts model confidence in area of the composite model – higher for the rigid-body fitted MTR4 fragments and de novo built ZCCHC8 dimerization module, and lower for the AlphaFold predictions and RBM7-binding module rigid-body fitted in low resolution map.

Figure S2. NEXT^S cryo-EM data quality and structural characteristics of the ZCCHC8 dimerization module (related to Figure 2)

A) Comparison of the MTR4 arch domain conformation. The inward arch conformation is revealed by the cryo-EM structure of the apo human MTR4 from the NEXT^S protomer (blue), the crystal structure of the apo human MTR4 (green) (PDB:6IEG) (Wang et al., 2019), and the crystal structure of the apo yeast Mtr4 in complex with RNA but without ATP (grey) (PDB:2XGJ) (Weir et al., 2010). The yeast MTR4 from the exosome-Mtr4-pre60S assembly (yellow) (PDB:6FSZ) (Schuller et al., 2018) displays the outward arch conformation.

B) and C) Local resolution analysis of the overall (B) and focused (C) NEXT^S complex reconstruction. Distribution of local resolution was estimated with RELION and colored accordingly.

D) and E) Spherical angular distribution of the NEXT^S complex particles used in the final 3D auto-refinement yielding the overall (D) and focused (E) reconstruction.

F) and G) The 3D Fourier Shell Correlation plots generated with the Salk Institute software (Tan et al., 2017). The red line represents the estimated global masked half-map FSC curve indicating an overall resolution for the overall NEXT^S reconstruction (F) and for the focused NEXT^S reconstruction (G), according to the gold standard FSC cut off of 0.143 (Rosenthal and Henderson, 2003).

H) and I) Model vs. map FSC plots for the real space refined model of the overall NEXT^S reconstruction (H) and for the focused NEXT^S reconstruction (I).

J) AlphaFold prediction of the ZCCHC8 N-terminal homodimerization region 1-260 shows overall structural similarity to the cryo-EM map interpretation performed in this study. The first ~40 residues in each protomer remain unstructured, as predicted by Falk et al., 2016, and are followed by the coiled-coil helices α1. In line with our findings, AlphaFold predicted a ZCCHC8 dimerization module composed of the central β-sheet with swapped β1 strands. Regions colored in red and orange correspond to the ZCCHC8 dimerization module built *de novo* in the 4.0 Å focused cryo-EM reconstruction of the NEXT^{S}. In the absence of the MTR4 KOW, ZCCHC8 residues 185-220 immediately following the ZCCHC8 AIM domain (highlighted with dotted circles), are predicted to extend the dimerization module through addition of two short anti-parallel β-strands. This is the major difference between the Alpha Fold model of ZCCHC8 in isolation and the experimentally derived cryo-EM model of MTR4-bound ZCCHC8. However, it is possible to envision that this region in ZCCHC8 may indeed fold against the ZCCHC8 dimerization module in the absence of MTR4 (as predicted by AlphaFold) and undergo a conformational change upon MTR4 binding (as visualized in the cryo-EM map for the complex).

K) Structural comparison of ZCCHC8 residues 177-220 (red) as shown by our experimental model (top panel) with the predicted AlphaFold model (bottom panel). In our model, the region assumes an extended conformation that wraps around the MTR4 KOW (indicated as a transparent blue surface). In the AlphaFold model, the two short anti-parallel β-strands (encircled residues 210-220) extend the ZCCHC8 dimerization module β-sheet in the absence of the MTR4 KOW.

L) AlphaFold prediction of the ZCCHC8 region 1-260, represented as ribbon and color-coded according to per-residue measure of local confidence called predicted local distance difference test (pLDDT). High confidence residues with pLDDT > 80, on a scale 0-100, are colored blue. Residues with pLDDT in the range 60-80 are colored cyan. Low confidence residues with pLDDT < 60 are colored orange.

M) Plot on the left shows per-residue measure of local confidence (pLDDT) for the AlphaFold prediction of the ZCCHC8 region 1-260, and corresponds with the residue colors in panel L. Plot on the right shows predicted aligned error (PAE). Dark green areas of the plot correspond with well folded fragments of the protein. Light green areas of the plot highlight low accuracy in predicted relative positions of folded domains.

N) Comparison of the AIMs domains from human ZCCHC8 (this study), NRDE2 (Wang et al., 2019), and nuclear VCP-like (NVL) (Lingaraju et al., 2019a) along with *S. cerevisiae* Nop53 (Falk et al., 2017b) and Air2 (Falk et al., 2014). Sequence alignment reveals the consensus sequence of the AIM to be X-F/W-X-L/I/V/T-D-X-X-G/P. The C-terminal glycine or proline residue allows the ZCCHC8 chain to bend away from the AIM-binding site.

O) Comparison of the KOW-interacting regions from ZCCHC8 (177-221) and NRDE2 (162-207), extending beyond the AIM. ZCCHC8 (red) and NRDE2 (green) share several conserved residues (depicted as sticks) that enable them to follow a similar binding pattern on the MTR4 KOW surface (grey). AIM (ZCCHC8 residues 177-184) and AIM+ (ZCCHC8 residues 185-220) are highlighted with black lines. ZCCHC8 and NRDE2 sequence alignment, at the bottom of the panel, shows conserved residues within their KOW-interacting regions.

Figure S3. Monomerized NEXT is sufficient to target some RNA substrates for degradation (related to Figure 3)

A) Analytical gel filtration comparison of the ZCCHC8(41-337)/RBM7(RRM) unit and the ZCCHC8(177-337)/RBM7(RRM) unit, resolved on the ÄKTAmicro equipped with Superdex 200 Increase 3.2/300 column. Retention volumes of molecular weight standards are highlighted for reference with thin dotted lines: ferritin (440 kDa), aldolase (158 kDa), conalbumin (75 kDa), ovalbumin (44 kDa), carbonic anhydrase (29 kDa), ribonuclease A (13.7 kDa).

B) RT-qPCR analysis of representative NEXT complex target transcripts (*proDNAJB4, proKLF6, proRBM39, proTTC32, 3'ext U11, 3'ext SNORD83a*). RNA was isolated from *ZCCHC8-3F-mAID* cell lines stably integrated with full length MYC-ZCCHC8$^{1-707}$, truncated MYC-ZCCHC8$^{177-707}$ or an empty vector (ev) as a negative control. Cells were either mock or treated with auxin (IAA) for 12 hours to deplete the endogenous ZCCHC8-3F-mAID protein. Results were normalized to *GAPDH* mRNA levels and plotted relative to *ZCCHC8-3F-mAID + ev - IAA control* samples. Columns represent the average values of technical triplicates per sample with error bars denoting the standard deviation. Individual data values from replicates are indicated as points.

Figure S4. ZCCHC8 linkers tether the ZCCHC8 ZnF and the RBM7 RRM domain at the RNA entry site within the MTR4 RecA2 domain (related to Figure 4)

A) Zoom-in onto the ZCCHC8 zinc-finger (ZnF) predicted with AlphaFold and rigid body fit in the 6.8 Å map of the single NEXT^{L} protomer. Conserved ZnF residues C229, C232, H237, and C242 are represented as sticks. The zinc atom was placed after aligning the structure of N-terminal zinc-finger domain from the HIV-1 nucleocapsid protein (PDB:1HVN) (South and Summers, 1993), found with the PDBeFOLD as the most similar to the ZCCHC8 zinc-finger. The AlphaFold-predicted α3 helix (residues 248-260) stabilizes the zinc-finger from the bottom. Linker connecting the AIM+ region with the zinc-finger is marked with a black arrowhead. Continuous density for the RNA spanning from the MTR4 channel, next to the zinc-finger, and beyond towards the RBM7 RRM is highlighted as a black dotted line. At the current resolution it was not possible to build in the nucleotides.

B) Zoom-in view of the tubular density connecting the ZCCHC8 α3 helix with the conserved motif located within the RecA2 cavity. The density is marked with a black arrowhead and the protein linker is depicted as red dashed line. The density is shown at lower map threshold (Chimera) compared to panels A and C, in order to emphasize better the linker connection.

C) Zoom-in view of the tubular density connecting the C-terminus of the ZCCHC8 Pro-rich region with the short β-strand aligned on the RecA2 domain β-sheet. The density is marked with a black arrowhead and the protein linker is depicted as red dashed line.

D) Zoom-in views comparison of ZCCHC8, NRDE2, and Air2 motifs bound within the MTR4 RecA2 domain pocket. A conserved acidic pocket on the RecA2 domain surface had previously been shown to bind positively-charged residues of human NRDE2 (Wang et al., 2019) and yeast Air2 (Falk et al., 2014). We speculate that a small additional density we observe within that pocket corresponds to a similar conserved motif Arg-Tyr-His (residues 273-275), preceding the Pro-rich domain of ZCCHC8.

E) Zoom-in views comparison of ZCCHC8, NRDE2, and Air2 motifs aligning as a β-strand on the existing MTR4 RecA2 domain β-sheet. A conserved edge of the

RecA2 β-sheet had previously been shown to interact with a β-strand of human NRDE2 (Wang et al., 2019) and yeast Trf4 (Falk et al., 2014). We speculate that a density we observe at that RecA2 β-sheet edge can be occupied by a predicted β-strand of ZCCHC8 (residues 334-337), following the Pro-rich domain of ZCCHC8.

F) Sequence alignment of ZCCHC8 showing the conserved motifs interacting with the MTR4 RecA2 domain.

Figure S5. ZCCHC8 CTD occludes the MTR4 RNA exit channel

(related to Figure 5)

A) Local resolution analysis of the focused MTR4 reconstruction from the $NEXT^L$ dataset. Distribution of local resolution was estimated with RELION and colored accordingly.

B) Spherical angular distribution of the $NEXT^L$ particles used in the final focused 3D auto-refinement of the MTR4

C) The 3D FSC plot generated with the Salk Institute software (Tan et al., 2017). The red line represents the estimated global masked half-map FSC curve indicating the resolution for MTR4 reconstruction, according to the gold standard FSC cut off of 0.143 (Rosenthal and Henderson, 2003).

D) Model vs. map FSC plot for the real space refined model of the focused MTR4 reconstruction.

E) Superposition of the MTR4-ZCCHC8 CTD X-ray structure (PDB:6C90)(Puno and Lima, 2018) with the MTR4-exosome structure (PDB:6D6R)(Weick et al., 2018)

F) The very C-terminal residues of the ZCCHC8 CTD (683-700) occupy the same surface cavity near the MTR4 ATPase site as the MPP6 N-terminal α-helix (5-21).

G) The N-terminal region of the ZCCHC8 CTD (residues 659-670) occupies the MTR4 surface used by the exosome cap protein RRP4 during MTR4-exosome interaction.

H) The interface between the ZCCHC8 CTD and the MTR4 RNA exit channel. Residues F673, E674, and E676 resemble RNA structure keeping the CTD locked in a position and blocking the RNA exit channel.

Figure S6. NEXT^{S}-EXO cryo-EM sample preparation and data processing (related to Figure 6)

A) Size exclusion chromatography and 15% SDS-PAGE of the NEXT^{S}-EXO sample used for cryo-EM structure determination. NEXT^{S} components are labeled in red.

B) Representative cryo-EM micrograph collected on a FEI Titan Krios operated at 300 kV, equipped with a K2 Summit camera.

C) Representative 2D classes from the initial selection of the NEXT^{S}-EXO particles. Box size (B) = 400 pix × 1.35 Å = 540 Å

D) Cryo-EM data processing scheme. 3D classification of particles showed one class (190,644 particles) corresponding to only the exosome core complex while another class (94,214 particles) showed additional density at the top of the exosome core. Particles from the latter class were re-extracted with a larger box size and re-centered on the NEXT^{S} ZCCHC8 dimerization module. Upon further rounds of 2D classification, we obtained a small class of particles corresponding to a NEXT^{S} homodimer bound to two exosomes (5,633 particles) and a larger class corresponding to a NEXT^{S} homodimer bound to a single EXO13 (11,273 particles). 3D classification of all particles used in the last 2D classification step led to a 9.5 Å 3D reconstruction that could be interpreted by fitting the known atomic models. Further 3D auto-refinement of that reconstruction, focused on MTR4-EXO9 reached 8.0 Å resolution.

E) NEXT-EXO model fitted in the 8.0 Å 3D auto-refinement. Region boxed with the black line is shown on the right of the panel, upon zooming-in and slicing the density in order to highlight the RNA substrate (red density and cartoon) – the RNA molecule from the superposed MTR4-exosome structure (PDB:6D6R) (Weick et al., 2018).

F) Gallery of the single representative extracted particles corresponding with a 2D class of the EXO-NEXT-EXO assembly (top row) and a 2D class of the NEXT-EXO assembly (bottom row). White “ruler” lines are highlighting positions of the NEXT and exosome complexes.

G) Mean pixel intensities quantified with ImageJ for the regions corresponding with the NEXT and exosome complexes within 2D classes from panel F. Similar amount of signal for both exosomes, as compared with the NEXT complex (top graph),

confirms that the EXO-NEXT-EXO 2D classes indeed represent an assembly with two exosomes, and do not originate from misclassification of the NEXT-EXO assembly. Mean pixel intensity of the background surrounding the particles was subtracted from each value before plotting.

H) Analytical gel filtration analysis of competitive binding between the ZCCHC8 CTD and the EXO9-MPP6-RRP6^{N}-RRP47 to the NEXT^{S} complex. 500 pmols equimolar amounts of the indicated recombinant protein samples were resolved on the Superdex 200 Increase 3.2/300 column and the peak fractions analyzed on a Coomassie stained 15% SDS PAGE. EXO9-MPP6-RRP6^{N}-RRP47 competes out the ZCCHC8 CTD for binding the NEXT^{S} complex (compare peaks 3.1 and 3.2 with peaks 5.1 and 5.2). Peaks 3.2 and 5.2 represent ZCCHC8 CTD not bound to the NEXT^{S}.

Figure S1

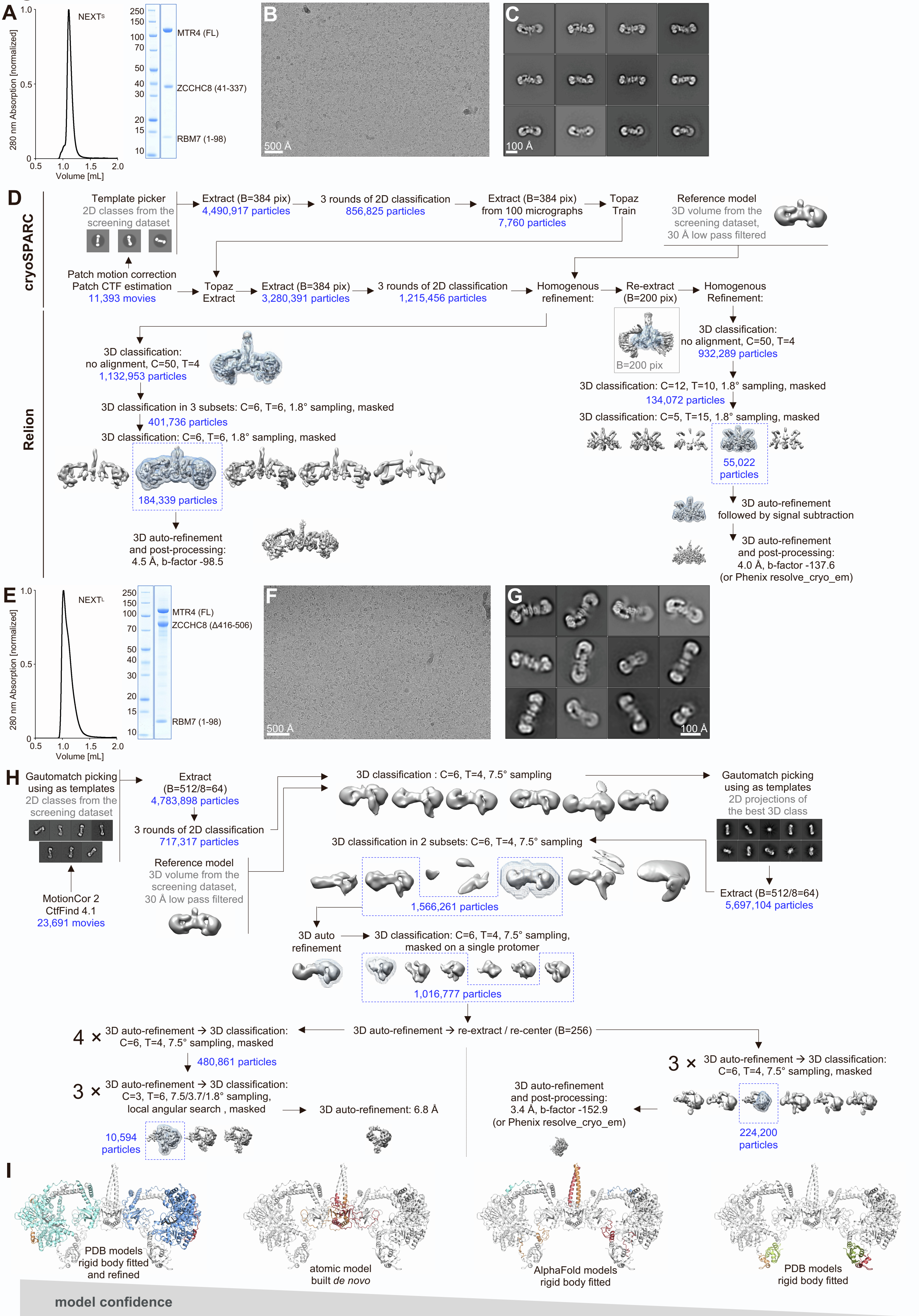
A
NEXTs
280 nm Absorption [normalized]
Volume [mL]
MTR4 (FL)
ZCCHC8 (41-337)
RBM7 (1-98)
B
500 Å
C
100 Å
D
cryoSPARC
Template picker
2D classes from the screening dataset
Extract (B=384 pix)
4,490,917 particles
3 rounds of 2D classification
856,825 particles
Extract (B=384 pix)
from 100 micrographs
7,760 particles
Topaz
Train
Reference model
3D volume from the screening dataset,
30 Å low pass filtered
Patch motion correction
Patch CTF estimation
11,393 movies
Topaz
Extract
Extract (B=384 pix)
3,280,391 particles
3 rounds of 2D classification
1,215,456 particles
Homogenous
refinement:
Re-extract
(B=200 pix)
Homogenous
Refinement:
Relion
3D classification:
no alignment, C=50, T=4
1,132,953 particles
3D classification in 3 subsets: C=6, T=6, 1.8° sampling, masked
401,736 particles
3D classification: C=6, T=6, 1.8° sampling, masked
184,339 particles
3D auto-refinement
and post-processing:
4.5 Å, b-factor -98.5
B=200 pix
3D classification:
no alignment, C=50, T=4
932,289 particles
3D classification: C=12, T=10, 1.8° sampling, masked
134,072 particles
3D classification: C=5, T=15, 1.8° sampling, masked
55,022
particles
3D auto-refinement
followed by signal subtraction
3D auto-refinement
and post-processing:
4.0 Å, b-factor -137.6
(or Phenix resolve_cryo_em)
E
NEXTL
280 nm Absorption [normalized]
Volume [mL]
MTR4 (FL)
ZCCHC8 (Δ416-506)
RBM7 (1-98)
F
500 Å
G
100 Å
H
Gautomatch picking
using as templates
2D classes from the screening dataset
MotionCor 2
CtfFind 4.1
23,691 movies
Extract
(B=512/8=64)
4,783,898 particles
3 rounds of 2D classification
717,317 particles
Reference model
3D volume from the screening dataset,
30 Å low pass filtered
3D classification : C=6, T=4, 7.5° sampling
Gautomatch picking
using as templates
2D projections of
the best 3D class
3D classification in 2 subsets: C=6, T=4, 7.5° sampling
1,566,261 particles
Extract (B=512/8=64)
5,697,104 particles
3D auto
refinement
3D classification: C=6, T=4, 7.5° sampling,
masked on a single protomer
1,016,777 particles
4 × 3D auto-refinement → 3D classification:
C=6, T=4, 7.5° sampling, masked
3D auto-refinement → re-extract / re-center (B=256)
480,861 particles
3 × 3D auto-refinement → 3D classification:
C=3, T=6, 7.5/3.7/1.8° sampling,
local angular search , masked
3D auto-refinement: 6.8 Å
10,594
particles
3 × 3D auto-refinement → 3D classification:
C=6, T=4, 7.5° sampling, masked
3D auto-refinement
and post-processing:
3.4 Å, b-factor -152.9
(or Phenix resolve_cryo_em)
224,200
particles
I
PDB models
rigid body fitted
and refined
atomic model
built de novo
AlphaFold models
rigid body fitted
PDB models
rigid body fitted
model confidence

Figure S2

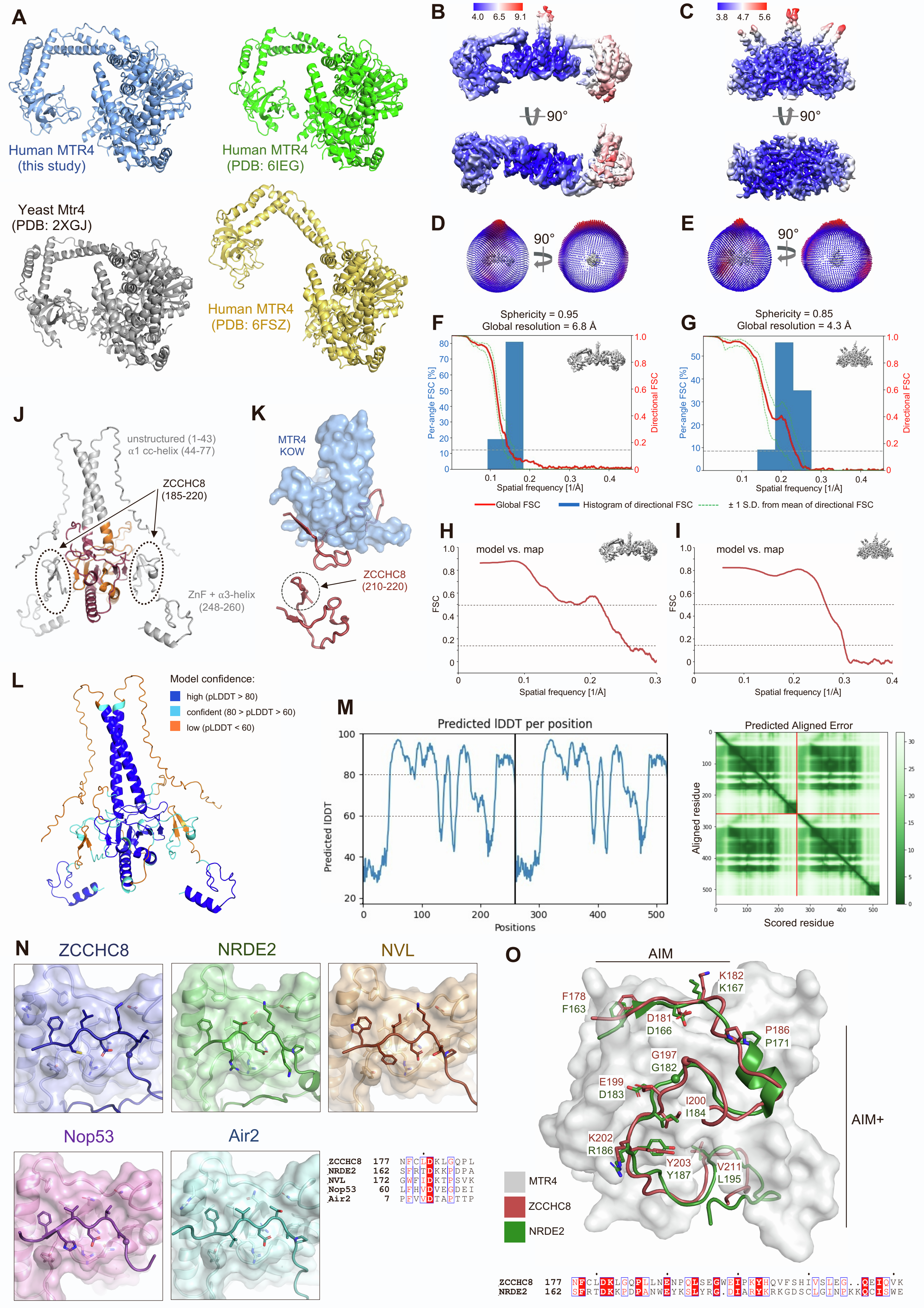

A
Human MTR4
(this study)
Human MTR4
(PDB: 6IEG)
Yeast Mtr4
(PDB: 2XGJ)
Human MTR4
(PDB: 6FSZ)
B
4.0 6.5 9.1
90°
C
3.8 4.7 5.6
90°
D
90°
E
90°
F
Sphericity = 0.95
Global resolution = 6.8 Å
Per-angle FSC [%]
Directional FSC
Spatial frequency [1/Å]
G
Sphericity = 0.85
Global resolution = 4.3 Å
Per-angle FSC [%]
Directional FSC
Spatial frequency [1/Å]
Global FSC
Histogram of directional FSC
± 1 S.D. from mean of directional FSC
H
model vs. map
FSC
Spatial frequency [1/Å]
I
model vs. map
FSC
Spatial frequency [1/Å]
J
unstructured (1-43)
α1 cc-helix (44-77)
ZCCHC8
(185-220)
ZnF + α3-helix
(248-260)
K
MTR4
KOW
ZCCHC8
(210-220)
L
Model confidence:
high (pLDDT > 80)
confident (80 > pLDDT > 60)
low (pLDDT < 60)
M
Predicted lDDT per position
Predicted lDDT
Positions
Predicted Aligned Error
Aligned residue
Scored residue
N
ZCCHC8
NRDE2
NVL
Nop53
Air2
ZCCHC8 177 NFCLDKLGQPL
NRDE2 162 SFRTDKKPDPA
NVL 172 GWFIDKTPSVK
Nop53 60 LFHVDVEGDEI
Air2 7 PFVVDTAPTTP
O
AIM
AIM+
K182
K167
F178
F163
D181
D166
P186
P171
G197
G182
E199
D183
I200
I184
K202
R186
Y203
Y187
V211
L195
MTR4
ZCCHC8
NRDE2
ZCCHC8 177 NFCLDKLGQPLNENPQLSEGWEIPKYHQVFSHIVSLEG..QEIQVK
NRDE2 162 SFRTDKKPDPANWEYKSLYRG.DIARYKRKGDSCLGINPKKQCISWE

Figure S3

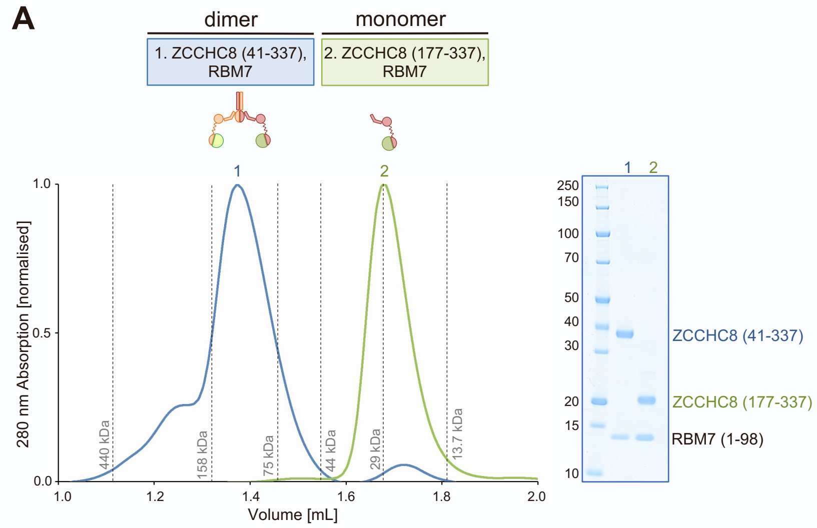

A
dimer
monomer
1. ZCCHC8 (41-337), RBM7
2. ZCCHC8 (177-337), RBM7
1
2
280 nm Absorption [normalised]
Volume [mL]
440 kDa
158 kDa
75 kDa
44 kDa
29 kDa
13.7 kDa
250
150
100
70
50
40
30
20
15
10
ZCCHC8 (41-337)
ZCCHC8 (177-337)
RBM7 (1-98)

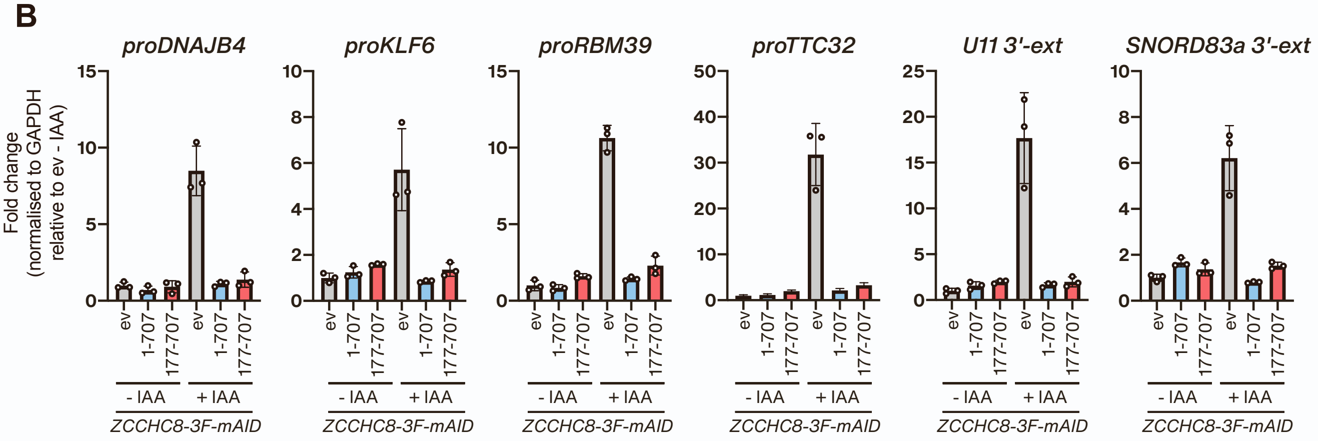

B
proDNAJB4
proKLF6
proRBM39
proTTC32
U11 3'-ext
SNORD83a 3'-ext
Fold change (normalised to GAPDH relative to ev - IAA)
ev
1-707
177-707
- IAA
+ IAA
ZCCHC8-3F-mAID

Figure S4

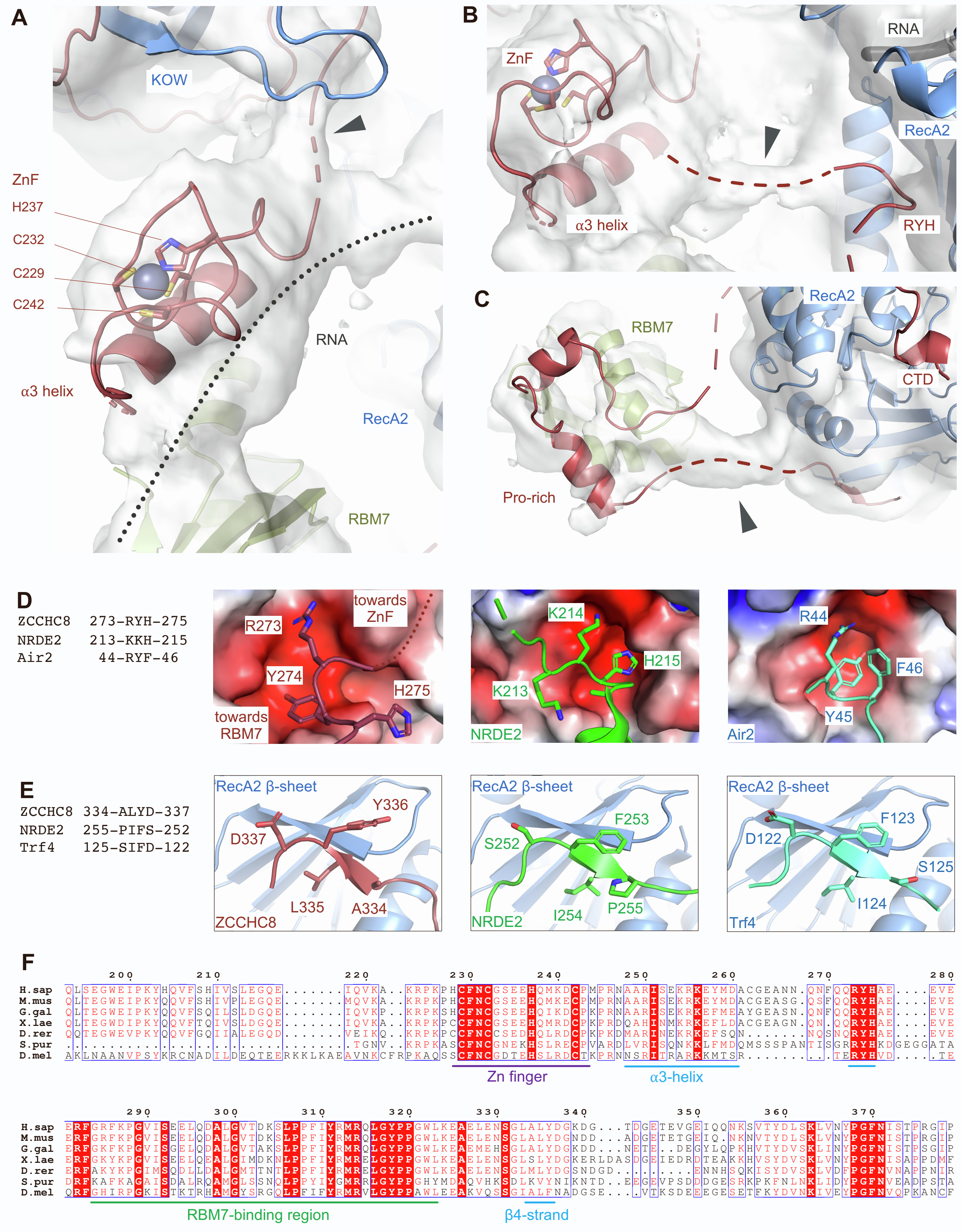
A
KOW
ZnF
H237
C232
C229
C242
α3 helix
RNA
RecA2
RBM7
B
ZnF
RNA
RecA2
α3 helix
RYH
C
RecA2
RBM7
CTD
Pro-rich
D
ZCCHC8 273-RYH-275
NRDE2 213-KKH-215
Air2 44-RYF-46
towards ZnF
R273
Y274
H275
towards RBM7
K214
H215
K213
NRDE2
R44
F46
Y45
Air2
E
ZCCHC8 334-ALYD-337
NRDE2 255-PIFS-252
Trf4 125-SIFD-122
RecA2 β-sheet
Y336
D337
L335
A334
ZCCHC8
F253
S252
I254
P255
NRDE2
F123
D122
S125
I124
Trf4
F
H.sap
M.mus
G.gal
X.lae
D.rer
S.pur
D.mel
Zn finger
α3-helix
RBM7-binding region
β4-strand

Figure S5

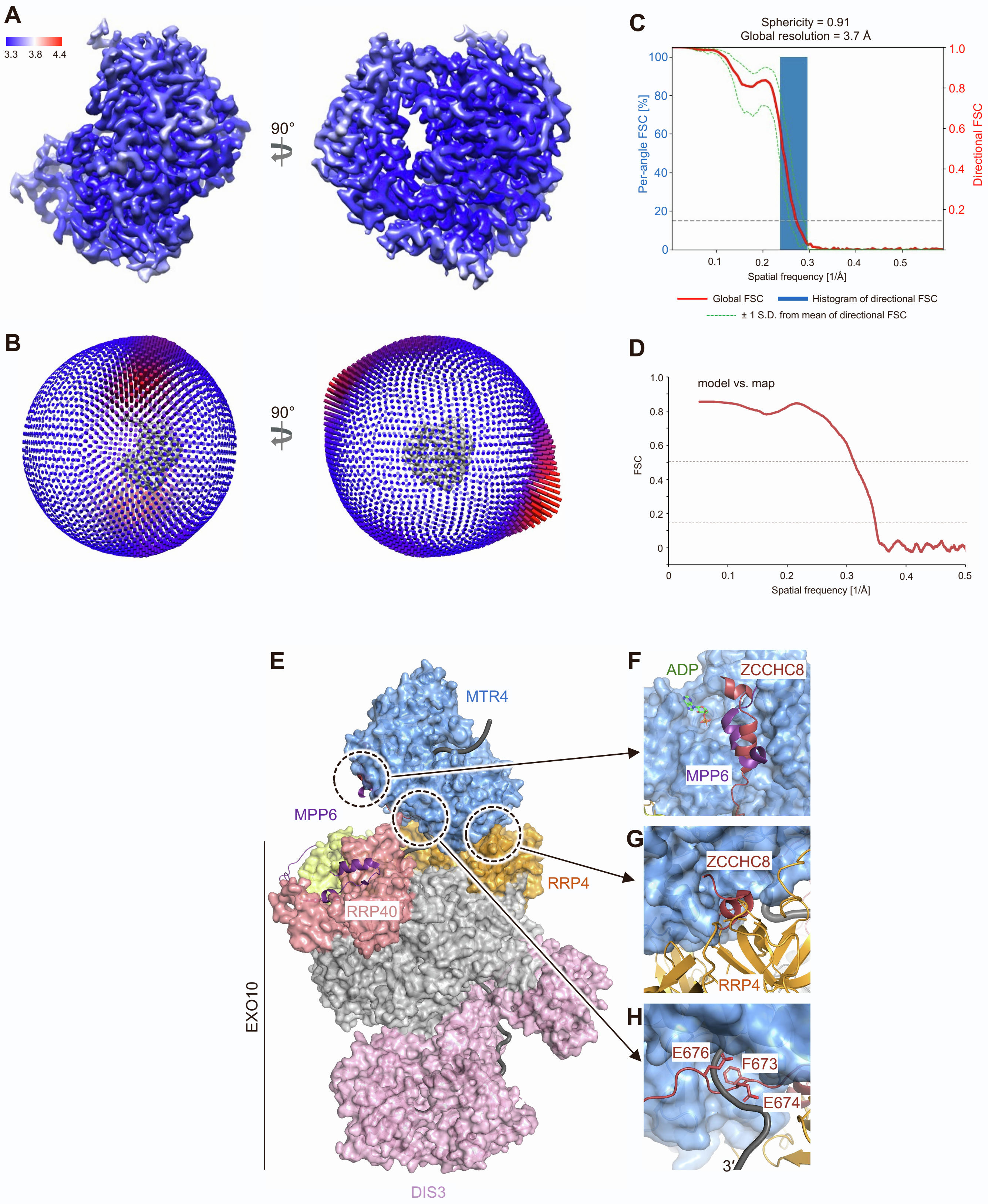
A
3.3 3.8 4.4
90°
B
90°
C
Sphericity = 0.91
Global resolution = 3.7 Å
Per-angle FSC [%]
Directional FSC
Spatial frequency [1/Å]
Global FSC
Histogram of directional FSC
± 1 S.D. from mean of directional FSC
D
model vs. map
FSC
Spatial frequency [1/Å]
E
MTR4
MPP6
RRP40
RRP4
EXO10
DIS3
F
ADP
ZCCHC8
MPP6
G
ZCCHC8
RRP4
H
E676
F673
E674
3'

Figure S6

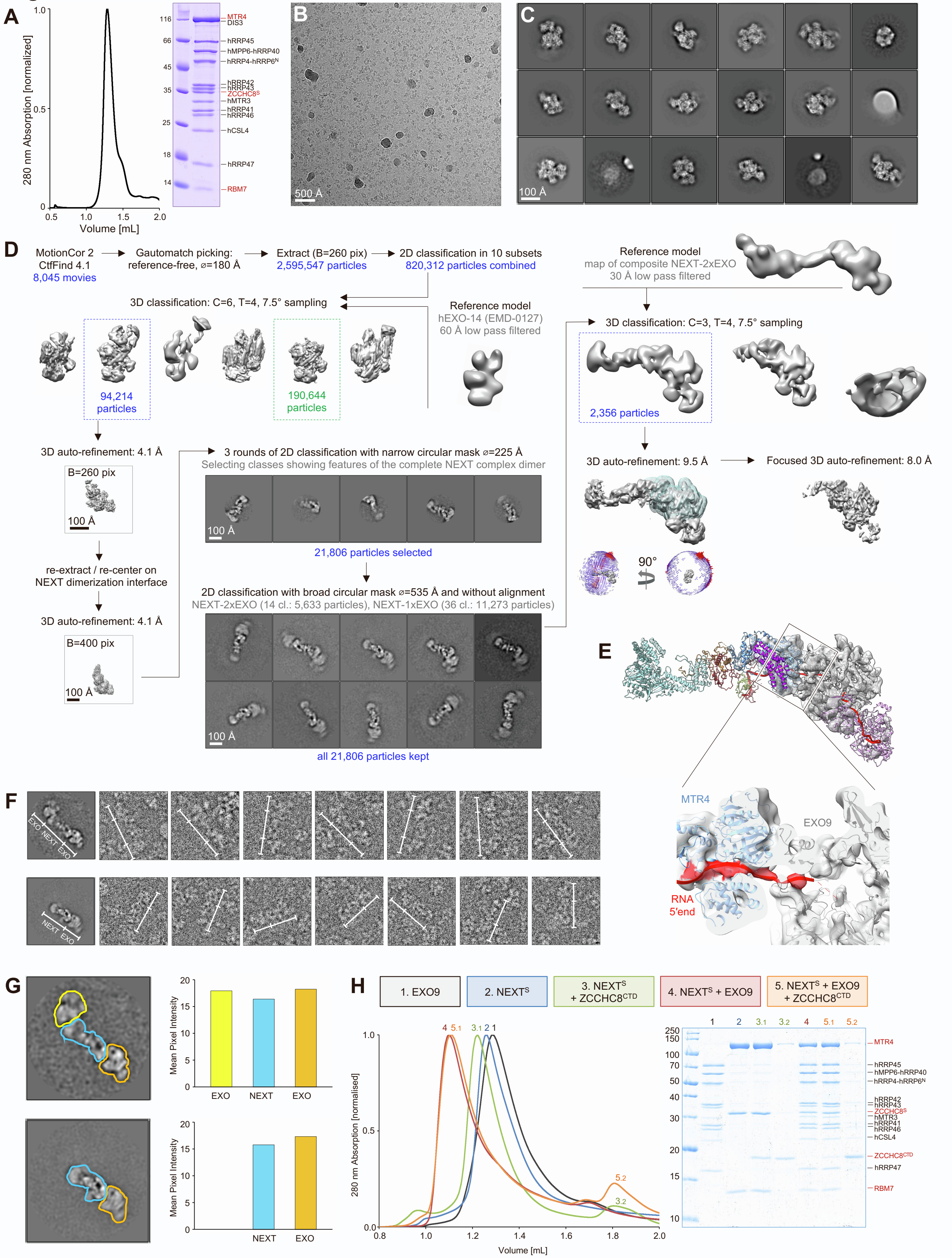

A
280 nm Absorption [normalized]
Volume [mL]
116
66
45
35
25
18
14
MTR4
DIS3
hRRP45
hMPP6-hRRP40
hRRP4-hRRP6N
hRRP42
hRRP43
ZCCHC8S
hMTR3
hRRP41
hRRP46
hCSL4
hRRP47
RBM7
B
500 Å
C
100 Å
D
MotionCor 2
CtfFind 4.1
8,045 movies
Gautomatch picking:
reference-free, ⌀=180 Å
Extract (B=260 pix)
2,595,547 particles
2D classification in 10 subsets
820,312 particles combined
3D classification: C=6, T=4, 7.5° sampling
Reference model
hEXO-14 (EMD-0127)
60 Å low pass filtered
94,214
particles
190,644
particles
3D auto-refinement: 4.1 Å
B=260 pix
100 Å
re-extract / re-center on
NEXT dimerization interface
3D auto-refinement: 4.1 Å
B=400 pix
100 Å
3 rounds of 2D classification with narrow circular mask ⌀=225 Å
Selecting classes showing features of the complete NEXT complex dimer
100 Å
21,806 particles selected
2D classification with broad circular mask ⌀=535 Å and without alignment
NEXT-2xEXO (14 cl.: 5,633 particles), NEXT-1xEXO (36 cl.: 11,273 particles)
100 Å
100 Å
all 21,806 particles kept
Reference model
map of composite NEXT-2xEXO
30 Å low pass filtered
3D classification: C=3, T=4, 7.5° sampling
2,356 particles
3D auto-refinement: 9.5 Å
Focused 3D auto-refinement: 8.0 Å
90°
E
MTR4
EXO9
RNA
5'end
F
EXO
NEXT
EXO
NEXT
EXO
G
Mean Pixel Intensity
EXO
NEXT
EXO
Mean Pixel Intensity
NEXT
EXO
H
1. EXO9
2. NEXTS
3. NEXTS + ZCCHC8CTD
4. NEXTS + EXO9
5. NEXTS + EXO9 + ZCCHC8CTD
280 nm Absorption [normalised]
Volume [mL]
4 5.1 3.1 2 1
5.2
3.2
1 2 3.1 3.2 4 5.1 5.2
250
150
100
70
50
40
30
20
15
10
MTR4
hRRP45
hMPP6-hRRP40
hRRP4-hRRP6N
hRRP42
hRRP43
ZCCHC8S
hMTR3
hRRP41
hRRP46
hCSL4
ZCCHC8CTD
hRRP47
RBM7

Table S1. Cryo electron microscopy data collection summary, processing statistics and model quality indicators (related to Figures 1, 2 and 6)

**Data collection**	**NEXT^{S}**		**NEXT^{L}**		**NEXT^{S} - EXO**
Microscope	FEI Titan Krios GII				
Voltage (kV)	300				
Camera	Gatan K3				Gatan K2
Energy Filter	Gatan Quantum-LS (GIF)				
Pixel size (Å/pix)	1.094		0.851		1.350
Defocus range (µm)	0.5 – 2.3				
**3D reconstruction**	**NEXT^{S} overall reconstruction**	**NEXT^{S} dimerization module**	**NEXT^{L} single protomer**	**NEXT^{L} focused on MTR4**	**NEXT^{S} with nuclear exosome**
Number of movies	11,393		23,691		8,245
Number of frames/movie	31		40		40
Exposure time (sec)	4.65		6		10
Total exposure (e^{-}/Å^{2})	64.85		68.20		46.86
Picked particle candidates	3,280,391		5,697,104		2,595,547
Final number of particles	184,339	55,022	10,594	224,200	2,356
Resolution (Å)[a]	4.5	4.0	6.8	3.4	9.5
Local resolution range (Å)	4.0 – 9.1	3.8 – 5.6	-	3.3 – 4.4	-
Sharpening B-factor	-98.5	-137.6	-210	-152.9	-
EMDB code	EMD-14510	EMD-14511	EMD-14514	EMD-14513	EMD-14515
**Refinement**					
PDB code	7Z4Y	7Z4Z		7Z52	
No atoms	16,762	4,828		5,911	
Residues (protein)	2,197	673		746	
Residues (RNA)	0	0		5	
CC_{box}, CC_{mask}, CC_{volume}[b]	0.69, 0.68, 0.68	0.57, 0.72, 0.69		0.65, 0.81, 0.77	
Resolution (Å)[b] FSC model vs. map (0 / 0.143 / 0.5)	3.4 / 3.9 / 5.6	3.2 / 3.3 / 3.8		2.5 / 2.9 / 3.2	
Bond lengths RMSD (Å)	0.003	0.002		0.004	
Bond angles RMSD (°)	0.761	0.700		0.601	
Ramachandran favored (%)	98.02	97.88		98.24	
Ramachandran allowed (%)	1.75	1.97		1.63	
Ramachandran outliers (%)	0.23	0.15		0.14	
MolProbity score	1.66	1.53		1.46	
Clash score	14.38	9.40		8.49	

[a]according to the Fourier Shell Correlation (FSC) cut-off criterion of 0.143 defined in (Rosenthal and Henderson, 2003)
[b]according to the model vs. map Correlation Coefficient definitions in (Afonine et al., 2018)

Table S2: sgRNA oligonucleotides (related to Figure 3)

NAME	FORWARD	REVERSE
ZCCHC8_gRNA	CACCGTAAGTCAAGCCATTATTCAG	AAACCTGAATAATGGCTTGACTTAC

Table S3: RTqPCR oligonucleotides (related to Figure 3)

NAME	FORWARD	REVERSE
proDNAJB4	TTTCTGGCGTTTCTGATTGA	ACCAAAACGCAGGTTGTTTA
proKLF6	AAGTTTTAGAGGGTCCGGCA	CTCTGCATAACCTTCACCG
proRBM39	AATAGATTTCCCTGTCATTTGGAGC	TTTCCAAGGTTGTTTCAAAGCTCG
proTTC32	GTCTGTTCCACGGTCCAAAC	ACAGCAGGCATGTAGGGTAG
U11 3'-ext	ACGCGTTTGGAGTAAGTGGT	GGTCACCTGCGGTTCATACA
SNORD83a 3'-ext	AGGGGAGACCTGTGGGTAAT	TGACCCCTTCCTGCTACTCA
GAPDH	GTCAGCCGCATCTTCTT	GCGCCCAATACGACCAAATC
